# A Phase 3, Multicenter, Randomized, Controlled Trial to Evaluate Immune Equivalence and Safety of Multidose and Single-dose Formulations of Vi-DT Typhoid Conjugate Vaccine in Healthy Filipino Individuals 6 Months to 45 Years of Age

**DOI:** 10.1016/j.lanwpc.2022.100484

**Published:** 2022-05-30

**Authors:** Josefina Cadorna Carlos, Birkneh Tilahun Tadesse, Charissa Borja-Tabora, Edison Alberto, Michelle C. Ylade, Arijit Sil, Deok Ryun Kim, Hyeon Seon Ahn, Jae Seung Yang, Ji Yeon Lee, Min Soo Kim, Jiwook Park, Soo-Young Kwon, Hun Kim, Seon-Young Yang, Ji-hwa Ryu, Hokeun Park, Jong-hoon Shin, Yoonyeong Lee, Jerome H. Kim, Zenaida Reynoso Mojares, T. Anh Wartel, Sushant Sahastrabuddhe

**Affiliations:** a University of the East-Ramon Magsaysay Memorial Medical Center Inc., Quezon City, Philippines; b International Vaccine Institute, Seoul, Republic of Korea; c Asian Hospital and Medical Center, Muntinlupa, Metro Manila, Philippines; d Medical Research Unit, Tropical Disease Foundation, Inc., Makati City, Metro Manila, Philippines; e University of the Philippines Manila-National Institutes of Health, Ermita, Manila, Philippines; f SK bioscience, Seongmam-si, Seoul, Republic of Korea

**Keywords:** Typhoid fever, Vi-DT vaccine, Immune equivalence, Single dose formulation, Multidose formulation

## Abstract

**Trial Design:**

Phase 3, randomized, controlled, multicenter, equivalence trial.

**Methods:**

Recruitment of participants occurred between 04Februray2020 and 15July2020 at four centers in the Philippines: University of the East – Ramon Magsaysay Memorial Medical Center Inc., Quezon City; University of Philippines Manila – National Institute of Health, Ermita Manila; Asian Hospital and Medical Center, Metro Manila, Philippines Study; and Medical Research Unit, Tropical Disease Foundation, Makati City, Metro Manila, Philippines.

**Participants:**

1800 adults and children 6-months to 45-years of age.

**Interventions:**

Participants received a single injection of multidose (MD) or single dose (SD) Vi-DT as test vaccines or meningococcal conjugate vaccine as a comparator.

**Objective:**

To evaluate immune equivalence of SD and MD formulations of Vi-DT, and to assess the safety of both formulations compared with comparator vaccine.

**Outcome Measurement:**

Blood draw for immunogenicity was performed at baseline prior to vaccine receipt and at four weeks after vaccination for a subset of participants to determine anti-Vi IgG geometric mean titers (GMT) and seroconversion rates. The primary outcome was comparison of anti Vi-IgG seroconversion and GMT between the two formulations of Vi-DT at 4 weeks following vaccine administration. Immune equivalence of MD and SD formulations was confirmed when the two-tailed 95% confidence interval (CI) of the GMT ratio is within [0.67, 1.5] at a two-sided significance level of 0.05. All participants were followed for safety events for six months after vaccine administration.

**Randomization:**

Participants were randomized to receive SD Vi-DT, MD Vi-DT, or meningococcal conjugate vaccines in 2.5:2.5:1 allocation ratio.

**Blinding:**

Study participants and observers were blinded to treatment assignment.

**Findings:**

Immune equivalence of SD (n=252) and MD (n=247) formulations was confirmed by anti-Vi IgG GMT ratio of 1.14 (95%CI: 0.91, 1.43) with respective GMTs in the MD and SD groups of 640.62 IU/mL (95%CI: 546.39, 751.11) and 562.57 IU/mL (95%CI: 478.80, 661.00) (*p*=0.259). Similarly, anti-Vi IgG seroconversion rate difference between the two formulations of ‒0.43% (95%CI: –4.42, 3.56) confirmed immune equivalence with corresponding seroconversion rates of 98.38% (95%CI: 95.91, 99.37) and 98.81% (95%CI: 96.56, 99.59) in MD and SD Vi-DT formulations, respectively (*p*=0.722). Both formulations of Vi-DT had a satisfactory safety profile – all five serious adverse events reported during the study were unrelated to the investigational product.

**Interpretation:**

The MD and SD formulations of Vi-DT elicited robust and equivalent immune responses following one dose vaccination, and both formulations demonstrated a favorable safety profile.

**Trial Registration:**

ClinicalTrials.gov: NCT04204096.

**Funding:**

This study was funded by the Bill & Melinda Gates Foundation (OPP 1115556).

**Research in context**

**Evidence before this study**

Typhoid conjugate vaccines play a critical role in preventing typhoid fever in endemic countries. The Vi diphtheria toxoid vaccine (Vi-DT) is a new generation of typhoid conjugate vaccine with robust immunogenicity and promising safety profile in a single dose formulation. Introduction of the vaccine in endemic settings requires suitable delivery methods including a multidose (MD) formulation. However, there is scarcity of evidence on the immune equivalence of a single dose (SD) formulation with a MD formulation of Vi-DT. We searched PubMed for reports of studies investigating or reviewing the equivalence of the single dose and multidose formulations of typhoid vaccines using iterations of the search terms: “typhoid vaccine”, “Vi polysaccharide”, “Ty21a”, “typhoid conjugate vaccine”, “multidose”, “single dose”, “formulations” and “equivalence” in the title and abstract of articles without restriction to language or publication date. We did not identify any relevant articles that attempted to evaluate the equivalence of the two formulations for any typhoid conjugate vaccine.

**Added value of this study**

Our findings present the first investigation of the safety and immune equivalence of SD and MD formulations of the Vi-DT typhoid conjugate vaccine. The findings confirmed immune equivalence of SD and MD formulations based on anti-Vi IgG geometric mean titers (GMT) ratio of 1.14 (95% Confidence Interval, CI: 0.91, 1.43) and the difference in anti-Vi IgG seroconversion rates between the two formulations of –0.43% (95%CI: −4.42, 3.56). The SD and MD formulations of Vi-DT typhoid conjugate vaccine demonstrated a satisfactory safety profile during the six month follow up period.

**Implications of all the available evidence**

The licensure and prequalification of the Vi-DT conjugate vaccine as SD and MD formulations will enhance the delivery options of the Vi-DT vaccine in public health programs including the expanded program of immunization (EPI). A MD formulation of Vi-DT will support the successful execution of vaccination campaigns in outbreak settings and hyperendemic settings while a SD formulation will support inclusion of the vaccine in EPI programs. There is a need for planning and implementation of real-world studies evaluating vaccine effectiveness in programmatic implementation settings and cost-effectiveness evaluation of both formulations.

Alt-text: Unlabelled box

## Introduction

Typhoid fever, caused by *Salmonella enterica* serovar Typhi (S. Typhi) is a major health problem in several low- and middle-income countries (LMIC) and an important cause of morbidity and mortality.^1^, ^2^, ^3^ Typhoid fever is more common in children and young adults than in older people and is most prevalent in impoverished areas that are overcrowded with poor access to sanitation. Surveillance has revealed a higher burden amongst pre-school children.^4^^,^^5^ Although typhoid fever can be effectively treated with antibiotics, growing rates of antibiotic resistance in many countries are making this treatment option increasingly more difficult and costly.^6^^,^^7^ Early intervention with vaccination, especially in the age group younger than 2 years, is essential.^8^^,^^9^

The WHO recommends that countries consider the use of typhoid vaccines for high-risk groups and populations, and for outbreak control.^10^ In endemic countries, control of typhoid requires implementing immunization for young children and incorporating typhoid vaccines in the expanded program of immunization (EPI). Typhoid vaccines that can help overcome the burden of disease have been licensed.^9^^,^^11^ First generation, heat inactivated whole cell vaccines have a high frequency of reactogenicity and led to withdrawal of these vaccines from routine immunization programs. Two types of second- generation vaccines – oral live attenuated vaccine and a subunit Vi-capsular polysaccharide vaccine – are efficacious but have several limitations especially inability to use in children under 2 years of age.^9^^,^^11^, ^12^, ^13^, ^14^

Most of the vaccines developed for the prevention of typhoid fever target the Vi capsular polysaccharide antigen, which is an important *S.* Typhi virulence factor responsible for invasive disease in humans.^15^ Although multiple Vi polysaccharide vaccines are licensed, only one produced by Sanofi Pasteur is pre-qualified by the WHO for individuals 2 years and older due to poor immunogenicity.^10^ However, the quality and quantity of these antibodies required to protect from clinical disease and/or infection, also referred to as the correlate of protection (CoP), has not been defined to date.

Currently, there are four commercially available TCVs all in India – Typbar TCV®, Vi polysaccharide conjugated to tetanus toxoid – Vi-TT (Bharat Biotech India Ltd, Hyderabad), PedaTyph, Vi polysaccharide conjugated to tetanus toxoid (Bio-Med Ltd, India), ZYVAC, Vi polysaccharide conjugated to tetanus toxoid (Zydus Cadila, India), and the recently licensed TYPHBEV – Vi polysaccharide conjugated to recombinant mutant *Pseudomonas aeruginosa* exoprotein  (Biological E Ltd, India).^16^, ^17^, ^18^, ^19^ The Vi-DT TCV – a Vi polysaccharide vaccine conjugated to diphtheria toxoid has undergone preclinical and clinical evaluation and is expected to be WHO prequalified and licensed in early 2022.^20^, ^21^, ^22^ After licensure, delivery of these vaccines can be facilitated in various ways including licensure of alternative dosing presentations. Presentation in multidose (MD) and single dose (SD) vaccine vial preparations overcomes critical supply and program implementation issues and improves uptake.^23^ However, little is known about the immune equivalence and safety of multidose formulations as these formulations are not often used in studies during the early clinical development programs.

Here, we present evidence from a multicenter, randomized, blinded, phase 3 trial evaluating the immune equivalence of the MD formulation against SD formulation of Vi-DT at 4 weeks after a single dose vaccination. We also describe the safety profile up to 24 weeks after vaccination with one dose of MD and SD formulations of Vi-DT in adults and children compared to a control group receiving the meningococcal conjugate vaccine (MCV-A).

## Methods

### Trial Design

A multicenter, phase 3, randomized, controlled, blinded trial among healthy Filipino adults and children between the ages of 6 months to 45 years to assess immune equivalence and safety of MD and SD Vi-DT formulations. The clinical trial has been registered at ClinicalTrials.gov registration: NCT04204096

#### Participants

Recruitment of participants occurred between 04Februray2020 and 15July2020 at four centers in the Philippines: University of the East – Ramon Magsaysay Memorial Medical Center Inc., Quezon City; University of Philippines Manila – National Institute of Health, Ermita Manila; Asian Hospital and Medical Center, Metro Manila, Philippines Study; and Medical Research Unit, Tropical Disease Foundation, Makati City, Metro Manila, Philippines. The trial enrolled healthy children and adults who voluntarily consented and/or assented to participate in the study, or whose parents or legal authorized guardians (LAR) provided consent to participate in the study and follow the study procedures during the entire period of the study. Participants with any of the conditions listed in the Supplementary Table S1 were excluded.

##### Discontinuation, Withdrawal and Loss-to-Follow up

Participants were discontinued from participation if an acute reaction (allergy, hypersensitivity reaction) to the investigational product was observed; in case of occurrence of an illness or serious adverse event or adverse event that in the judgment of the investigator might be detrimental for the participant's safety; if a study participant or parent or LAR withdraws informed consent; and in case of study participant's no-show for a scheduled visit without notice and becoming untraceable. A participant was considered lost to follow-up if he or she failed to show up for a scheduled visit and remains unreachable to study site staff for at least 3 documented attempts.

#### Vaccines and Vaccination Schedule

##### Investigational Product

The investigational product for the study was the Vi-DT typhoid conjugate vaccine developed and manufactured by SK bioscience Co., Ltd., Republic of Korea. The vaccine was prepared in MD and SD preparations. Both MD and SD formulations had similar labelling, packaging, and storage requirements (Supplementary Table S2). The dose of Vi-DT was confirmed in previous clinical trials of the vaccine.^20^, ^21^, ^22^

###### Vi-DT MD Formulation

The Vi-DT MD formulation is a 3mL formulation (code name: NBP618-MD) which is a colorless liquid and contains a purified Vi-polysaccharide conjugated to diphtheria toxoid with the preservative 2-PE. Each vial includes 25µg of Vi polysaccharide/0.5 mL with 5 doses per each multidose vial presented in Type I glass vial. The remainder of the 3mL vial was discarded. Each participant assigned to the Vi-DT MD group received 0.5 mL by intramuscular injection in the left anterolateral thigh or left arm deltoid region if below 2 years of age, and in the less dominant arm deltoid region in the 2-45 years age group.

###### Vi-DT SD Formulation

The Vi-DT SD formulation (code: NBP618-SD) is a 0.5mL formulation presented as a colorless liquid in a Type I glass vial. Each vial included 25 µg of Vi polysaccharide with no preservatives.

###### Vi-DT Vaccine Packaging, Labelling and Storage

Both formulations of the Vi-DT vaccine were similarly manufactured and packaged by SK bioscience and supplied to the study pharmacy nurse at the clinical trial site. The vaccine was labelled by SK bioscience as Test Vaccine (Vi-DT). Each vial was labelled and packaged following the local health authority guidelines and the study design. Vaccine vials were protected from light and stored at +2°C to 8°C.

##### Control Vaccine

The control vaccine for the study was the meningococcal conjugate vaccine (Nimenrix® Pfizer) administered through intramuscular route in a 0.5 mL volume as a single dose vial. A booster dose of Nimenrix® was administered for infants from 6 weeks to less than 1 year of age after study completion.

###### Control Vaccine Packaging, Labelling and Storage

Supplied as single dose glass vial as a powder and separate solvent solution for injection. The original label as registered in the Philippines was maintained. Vials were protected from light and stored at +2°C to 8°C.

#### Study Procedures and Follow up

##### Assessments

Each participant had five follow up visits: pre-screening (visit 1), vaccination (visit 2), safety assessment at 1 week (visit 3), immunogenicity and safety assessment at 4 weeks (visit 4) and safety assessment at 6 months (visit 5).

##### Safety

Participants were monitored for 30 minutes to assess immediate reactogenicity at the time of vaccination, solicited adverse events (AEs) within the first week of vaccination, immunogenicity and unsolicited adverse event evaluation at four weeks, and serious adverse event (SAE) assessment until 6 months post vaccination. A diary card was used to record solicited and unsolicited adverse events. Furthermore, relevant medical events were listed to assist in the classification of adverse events. All AEs were classified using the System Organ Class (SOC) and preferred term (PT) using MedDRA (version 22.1 and version 23.1 for COVID-19 terms).^24^

##### Immunogenicity

Venous blood was collected from participants for immunogenicity assessment from the adult age group before vaccine administration and at four weeks post vaccination. Whole blood was centrifuged, and serum aliquoted and stored at below -20°C until shipment to the central laboratory at the International Vaccine Institute (IVI) for testing. Aliquoted serum samples for immunogenicity assessment were shipped from the clinical trial sites to the IVI, where they were stored at -70°C until analysis. Anti-Vi IgG was determined using enzyme-linked immunosorbent assay (ELISA), which was validated in previous studies.^20^, ^21^, ^22^^,^^25^ Briefly, poly-L-lysine pre-coated and purified with Vi polysaccharide antigen at a concentration of 2 μg/mL was absorbed onto 96-well microtiter plates. Nonspecific binding sites were blocked with Bovine Serum Albumin (BSA) in Phosphate Buffered Saline (PBS); diluted human sera were then added to the first wells in the plate followed by serial dilution across the plates. Next, the anti-Vi IgG level was quantified using alkaline phosphatase labelled goat anti-human IgG where addition of 4-nitrophenyl phosphate substrate resulted in color change proportional to the amount of human anti-Vi IgG antibody present in the serum. Finally, optical densities of the wells were measured at 405 nm. The level of the specific anti-Vi IgG in international unit per mL for each serum sample was determined by comparison to an international standard serum, NIBSC 16/138. Seroconversion was defined as a greater than four-fold increase in the ant-Vi IgG titers from baseline.

#### Sample size

The sample size of 1800 participants was calculated to power the study for assessing equivalence of immunogenicity between SD and MD formulations of Vi-DT, and assessment of safety across age strata. The immunogenicity subset included 250 participants per treatment group providing 94% power to demonstrate immune equivalence based on GMT of anti-Vi IgG at four weeks (28 days) after vaccination using MD Vi-DT and SD Vi-DT, with an equivalence margin for the GMT ratio of 0.67 to 1.5 based on the WHO recommendation (WHO TRS 924). Coefficient of variation (CV) of immunogenicity titer was conservatively assumed as 2.0 based on data from previous Vi-DT trials,^20^ type 1 error rate of 0.05 and 10% drop out rate. Similarly, a sample size of 250 participants per treatment group provided 95% power to demonstrate immune equivalence based on seroconversion rates between the two formulations of Vi-DT with equivalence margin of [–10%, 10%], assuming a seroconversion rate of 90% in those who received Vi-DT according to data from previous studies,^20^, ^21^, ^22^ type 1 error rate of 0.05. A control group of 300 participants receiving the MCV-A was estimated assuming at least one severe adverse event assuming incidence of 1% according to “rule of three”.

#### Randomization and masking

A randomization list was generated by an independent statistician at IVI who was not directly involved in the study conduct. Eligible participants were assigned to receive one of MD Vi-DT, SD Vi-DT, or control vaccine in 2.5:2.5: 1 allocation ratio. A block randomization process ensured an effective balance between the interventions. Participants were randomized into three treatment groups within the age strata: Age Stratum 1: 18 to 45 years; Age Stratum 2: 2 to less than 18 years; and Age Stratum 3: 6 months to less than 2 years. The randomization information was only available to the study nurse/pharmacist and independent statistician.

The study was participant and observer blind with respect to assignment to test or control vaccines. Trial staff other than the unblinded study staff remained blinded to vaccine administration. The unblinded study nurse and the unblinded pharmacist were not involved in the evaluation of vaccine safety and were not allowed to discuss with the investigator and clinical trial staff about the vaccines administered. Unblinding was considered on a case-by-case basis and only in the case of a life-threatening condition or serious medical emergency when the vaccine allocation is judged relevant for the safety of the participants. There was no case requiring unblinding during the study period. An internal, independent Safety Monitoring Committee (SMC) and DSMB also reviewed SAEs in an unblinded manner and made recommendations as applicable. Reporting of safety events was performed in compliance with applicable regulatory requirements.

#### Statistical Analysis

##### Baseline Demographics

Demographic characteristics and other baseline data of enrolled participants were summarized by treatment group. Continuous variables such as age, height and weight were summarized by number of participants, mean, standard deviation, median, minimum, and maximum. Categorical variables such as sex were summarized by frequency and percentage by treatment assignment. Differences in difference in baseline characteristics were assessed using ANOVA or Kruskal-Wallis test for continuous variables, and Chi square test or Fisher's exact test for categorical variables.

##### Primary Endpoint Comparison

The primary endpoint was to demonstrate equivalence of the two Vi-DT formulations (MD vs. SD) using GMT ratio (MD/SD). Equivalence of the two formulations was confirmed if the two-tailed 95% confidence interval of the GMT ratio was within [0.67, 1.5] at a two-sided significance level of 0.05.

##### Secondary Endpoint Comparison

The secondary endpoint was to demonstrate equivalence of the two Vi-DT formulations (MD vs. SD) using the difference (MD *minus* SD) in seroconversion rate. Equivalence was demonstrated if the two-tailed 95% confidence interval of the estimate of difference of seroconversion at four weeks (Day 28) was located within [–10%, 10%] at a significance level of 0.05.

Safety endpoints were descriptively summarized by stratification of each formulation and within each age stratum. Number of AEs and proportion of participants with safety events following vaccination were summarized including 95% confidence interval of the proportion and p-value for group comparison within each age stratum. SAEs and AEs that led to participant early drop out were recorded in a line list.

All statistical analyses were performed using SAS 9.4 (SAS Institute, Cary NC) according to the protocol and the approved Study Reporting Statistical Analysis Plan, provided as a supplement.

##### Analysis Sets

The Full Analysis Set (FAS) included all 1800 randomized participants who received one dose of the investigational product in a modified intention to treat (m-ITT) analysis to assess demographic information and safety evaluation. As a subset of the FAS, the Immunogenicity Analysis Set (IAS) included participants in the adult age stratum who received the assigned treatment and at least one post-vaccination immunogenicity data. The Per-Protocol (PP) analysis set was a subset of the IAS including participants with no major protocol deviations defined as those compromising participant safety and scientific integrity of the data. Protocol deviations were defined based on adherence to inclusion-exclusion criteria, compliance with study procedures, completion of all visits as scheduled and receipt of correct vaccination.

#### Ethical Considerations

This clinical trial was conducted in accordance with ICH-GCP E6 (R2) Guidelines, the Declaration of Helsinki, Council for International Organizations of Medical Science (CIOMS) and local country's ethical requirements. The protocol was approved by the Single Joint Research Ethics Board (SJREB), Philippines Food and Drug Administration (PFDA), the respective Institutional Review Boards (IRB) of the four sites and the IVI IRB. All participants, parents or legal guardians participated voluntarily and signed an informed consent/assent following a test of understanding administered to the participants, parents, or legal guardians. A copy of the informed consent document was provided to the participants, parent, or legal guardian. Confidentiality of all participants was maintained throughout the study.

#### Role of the Funding Source

The Funder, Bill and Melinda Gates Foundation, did not play active role in the design, data collection, data analysis, interpretation, and writing of the manuscript. The authors, however, did discuss the design and overall clinical development strategy with the funder on a regular basis as part of the grant agreement.

### Results

#### Study Participants

**Figure 1:**
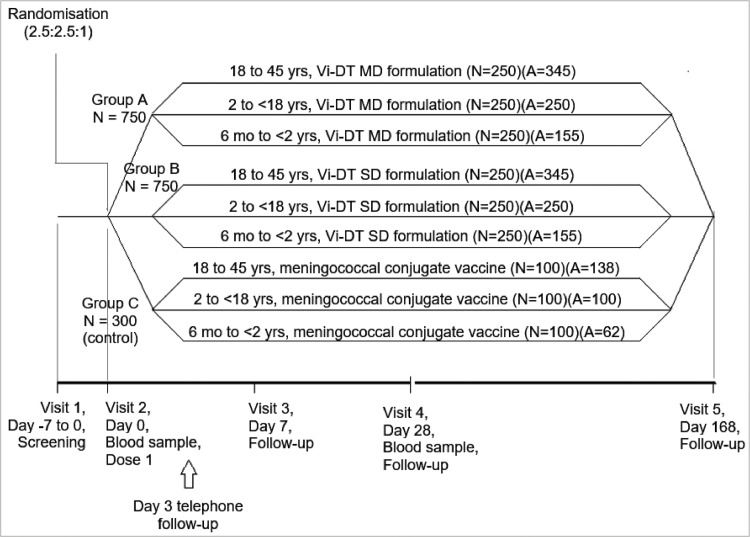
Participant allocation, randomization and visit schedule. N= planned number; A=actual enrollment. The planned and actual enrolment numbers are different across groups due to the revised.

The attrition of study participants is presented as a consort flow diagram in Figure 1. A total of 1825 participants aged 6 months to 45 years were screened. Of these 1800 participants were randomized in a 2.5:2.5:1 allocation ratio to one of the three treatment groups: 750 received MD Vi-DT (Group A), 750 received SD Vi-DT (Group B), and 300 received MCV-A (Group C). Participants were enrolled across three age strata: Age Stratum 1 (n=828), Age Stratum 2 (n=600), and Age Stratum 3 (n=372). The immunogenicity subset included participants from Age Stratum 1 (n=828; 345 participants each from the SD Vi-DT and MD Vi-DT groups and 138 participants from the control group). However, 226 participants were excluded from the IAS (98 from group A, 93 from Group B, and 35 in Group C) as follow up for immunogenicity blood draw could not be performed due to the COVID-19 outbreak related lockdown in the Philippines. All the participants were included in the safety analysis set. One participant was excluded from the PP analysis set since the four-week post-vaccination immunogenicity visit could not be completed within the allowable window (two weeks).

**Figure 2:**
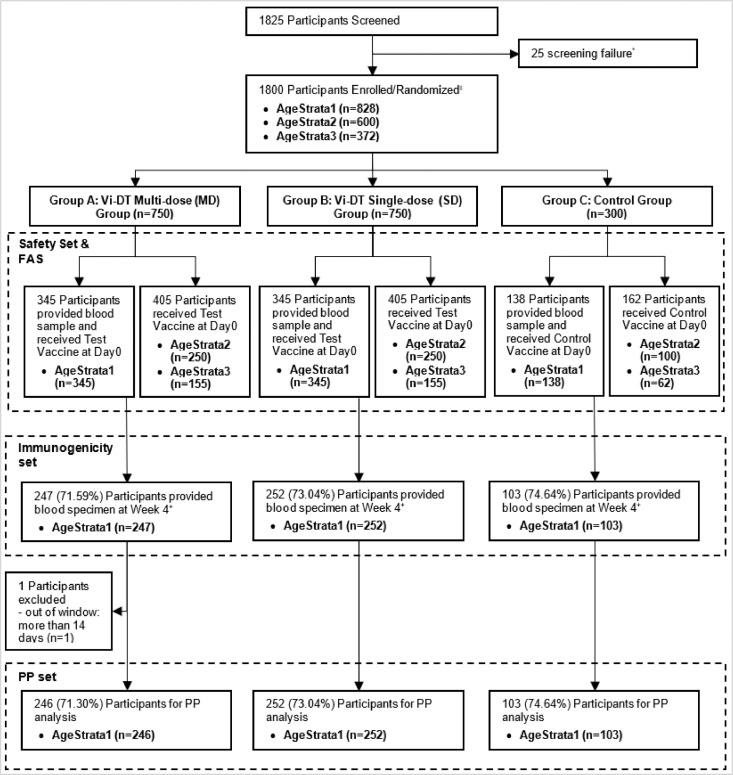
Consort diagram showing disposition of study participants.

#### Baseline Sociodemographic Characteristics

Baseline sociodemographic characteristics of the participants are presented in Table 1. Participants randomized in the three treatments groups had similar characteristics. There were no differences in the proportion of participants with medical history or history of prior or concomitant medications in the three treatment groups for all ages and within each age stratum.

**Table 1:** Baseline sociodemographic characteristics of the included study participants.

Characteristics		Vi-DT (MD)	Vi-DT (SD)	Control	Total	P-value
Overall		N=750	N=750	N=300	N=1800	
Gender	Male (%)	345 (46.00)	328 (43.73)	121 (40.33)	794 (44.11)	0.2386[1]
	Female (%)	405 (54.00)	422 (56.27)	179 (59.67)	1006 (55.89)	
Age (years)	Mean (Std Dev)	17.45 (12.91)	17.33 (12.99)	17.77 (13.41)	17.45 (13.02)	0.8872[2]
	Median (min, max)	16.12 (0.50, 44.97)	15.63 (0.51, 44.72)	16.05 (0.55, 44.47)	15.90 (0.50, 44.97)	0.9281[3]
18 to 45 years		N=345	N=345	N=138	N=828	
Gender	Male (%)	137 (39.71)	129 (37.39)	47 (34.06)	313 (37.80)	0.5011[1]
	Female (%)	208 (60.29)	216 (62.61)	91 (65.94)	515 (62.20)	
Age (years)	Mean (Std Dev)	29.32 (7.71)	29.47 (7.43)	30.34 (7.56)	29.55 (7.57)	0.3936[2]
	Median (min, max)	28.12 (18.02, 44.97)	27.85 (18.15, 44.72)	29.73 (18.28, 44.47)	28.28 (18.02, 44.97)	0.3453[3]
2 to less than 18 years		N=250	N=250	N=100	N=600	
Gender	Male (%)	130 (52.00)	115 (46.00)	41 (41.00)	286 (47.67)	0.1393[1]
	Female (%)	120 (48.00)	135 (54.00)	59 (59.00)	314 (52.33)	
Age (years)	Mean (Std Dev)	11.12 (4.50)	10.51 (4.58)	10.64 (4.63)	10.79 (4.56)	0.3052[2]
	Median (min, max)	12.37 (2.05, 17.95)	11.97 (2.16, 17.89)	11.70 (2.03, 17.82)	12.16 (2.03, 17.95)	0.3190[3]
6 months to less than 2 years		N=155	N=155	N=62	N=372	
Gender	Male (%)	78 (50.32)	84 (54.19)	33 (53.23)	195 (52.42)	0.7847[1]
	Female (%)	77 (49.68)	71 (45.81)	29 (46.77)	177 (47.58)	
Age (years)	Mean (Std Dev)	1.25 (0.43)	1.31 (0.42)	1.28 (0.46)	1.28 (0.43)	0.3801[2]
	Median (min, max)	1.25 (0.50, 1.98)	1.38 (0.51, 1.99)	1.28 (0.55, 1.97)	1.33 (0.50, 1.99)	0.4303[3]

^†^P-values for gender comparison and age (Mean, Median) comparison have been derived using [1] Pearson's Chi-square test, [2] one-way ANOVA and [3] Kruskal-Wallis test. (Vi-DT (MD) vs. Vi-DT (SD) vs. Control).

#### Immune Equivalence of MD and SD Formulations of Vi-DT Based on GMT

**Figure 3:**
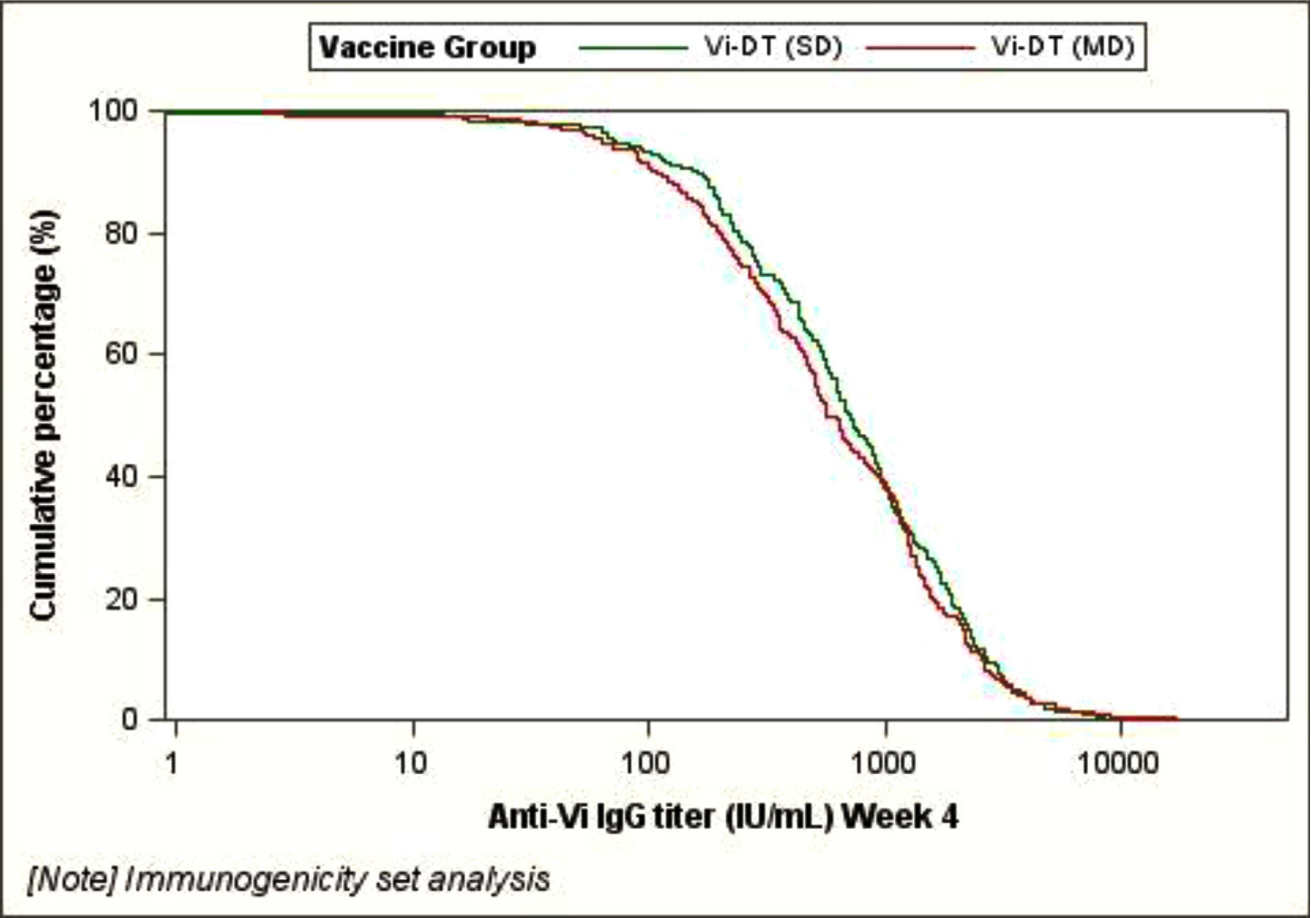
Reverse Cumulative Distribution Curve of Anti-Vi IgG Response using the Immunogenicity Anlysis Set. The X-axis represents the anti-Vi IgG geometric mean titers (GMT) in IU/mL while the y-axis represents the cumulative percentage of participants with the specific measured GMT on the X-axis.

Using the immunogenicity analysis set, immune equivalence of MD and SD formulations of Vi-DT was demonstrated using an anti-Vi IgG GMT MD/SD ratio of 1.14 (95%CI: 0.91, 1.43); anti-Vi IgG GMT was 640.62 IU/mL (95% CI: 546.39, 751.11) and 562.57 IU/mL (95% CI: 478.80, 661.00) in the groups assigned to MD and SD formulations of Vi-DT, respectively (*p* =0.259). Figure 3 shows the reverse cumulative distribution curves (RCDC) for anti-Vi IgG antibody titer at Week 4 in the groups receiving MD and SD formulations of Vi-DT, and Table 2 presents the comparison of GMTs of anti-Vi IgG response between the MD and SD formulations of Vi-DT and the control group at Day 0 and week 4 using the IAS.

**Table 2:** Geometric mean titers of anti-Vi IgG using the Immunogenicity Analysis Set in the adult age stratum.

Time point	Vi-DT (MD)		Vi-DT (SD)		Control		Vi-DT(MD) / Vi-DT(SD)	P-value
	N	GMT* (95% CI)	N	GMT* (95% CI)	N	GMT* (95% CI)	Ratio (95% CI)†	
Day 0	247	2.18 (1.81, 2.63)	252	1.77 (1.48, 2.11)	103	2.16 (1.61, 2.91)	1.24 (0.95, 1.60)	0.1080[1] 0.2303[2] 0.9976[3] 0.3608[4]
Week 4	247	640.62 (546.39, 751.11)	252	562.57 (478.80, 661.00)	103	2.51 (1.77, 3.58)	1.14 (0.91, 1.43)	0.2592[1] <0.0001[2] <0.0001[3] <0.0001[4]

[Note] N: number of total participants;

⁎ Geometric Mean Titers (unit: IU/ml); CI: Confidence Interval.

† The equivalence of a multidose (MD) formulation compared to a single dose (SD) was confirmed if both limits of two-tailed 95% confidence interval of the fold difference of GMT between two formulations of Vi-DT was within the equivalence margin of [0.67, 1.5]. [1] The P-value was derived from two sample t-test after log transformation between Vi-DT MD vs. SD. [2] The P-value was derived from ANOVA among 3 groups (Vi-DT MD vs. SD vs. Control). [3] The P-value was derived from Dunnett's procedure that was followed to compare the Vi-DT MD vs. Control. [4] The P-value was derived from Dunnett's procedure that was followed to compare the Vi-DT SD vs. Control.

The PP analysis set of GMT ratio and GMTs within the MD Vi-DT, SD Vi-DT and control groups mirrored the findings in the IAS (Supplementary Table S3).

#### Immune Equivalence of MD and SD Formulation of Vi-DT Based on Seroconversion

Using the immunogenicity analysis set, immune equivalence of the anti-Vi IgG seroconversion rates in MD and SD formulations of Vi-DT was demonstrated using the difference in anti-Vi IgG seroconversion rates between the two formulations of –0.43 (95%CI: –4.42, 3.56). The MD and SD formulations of Vi-DT elicited comparable seroconversion rates: 98.38% (95%CI: 95.91, 99.37) in MD and 98.81% (95%CI: 96.56, 99.59) in SD formulations of Vi-DT (*p*=0.722). Table 3 presents the seroconversion rates of anti-Vi IgG ELISA response in groups assigned to MD and SD formulations of Vi-DT and in those assigned to the control group at week 4 (28 days), using the IAS, demonstrating immune equivalence of MD and SD formulations of Vi-DT.

**Table 3:** Seroconversion rates of anti-Vi IgG in the single dose, multidose and control groups using the Immunogenicity Analysis Set in the adult age stratum.

Time point	Vi-DT (MD)		Vi-DT (SD)		Control		Vi-DT (MD) – Vi-DT (SD)	P-value
	n/N	Seroconversion rate* (95% CI)	n/N	Seroconversion rate* (95% CI)	n/N	Seroconversion rate* (95% CI)	Difference (95% CI)†	
Week 4	243/247	98.38 (95.91, 99.37)	249/252	98.81 (96.56, 99.59)	6/103	5.83 (2.70, 12.13)	–0.43 (–4.42, 3.56)	0.7222[1] <0.0001[2] <0.0001[3] <0.0001[4]

[Note] MD: Multidose; SD: Single Dose; CI: Confidence Interval; N: number of total participants; n: number of participants seroconverted.

⁎ Proportion of participants who had at least 4-fold rise anti-Vi IgG ELISA antibody titers at 4 weeks after vaccination compared to baseline (Day 0; prior to vaccination).

† The equivalence of two formulations was confirmed if the both limits of two-tailed 95% confidence interval of the difference of seroconversion rate between two formulations of Vi-DT was within the equivalence margin of [-10%, 10%]. [1] The P-value was derived from Fisher's exact test between Vi-DT MD vs. SD. [2] The P-value was derived from Chi-square test among 3 groups (Vi-DT MD vs. SD vs. Control). [3] The P-value was derived from Chi-square test between Vi-DT MD vs. Control. [4] The P-value was derived from Chi-square test between Vi-DT SD vs. Control.

Immune equivalence was similarly demonstrated using the PP analysis set with comparable differences and seroconversion rates (Supplementary Table S4).

#### Safety of Vi-DT in Children and Adults

The pooled safety events across all the sites are presented here; the frequency of AEs was few limiting analyses by site.

##### Immediate Reactogenicity

A total of 24 immediate reactions, mostly mild to moderate, occurred across all age groups in 23 participants: 13(1.73%), 7(0.93%) and 3(1.00%) participants, respectively, from the MD Vi- DT, SD Vi-DT, and the control groups (*p*=0.346). There was no difference in the occurrence of immediate reactions by age strata (Table 4; Supplement Table S5 – S7). The most common local immediate reaction observed in all groups was pain/tenderness at the site of injection occurring in 13(1.73%) participants. There were 5(0.67%) participants in the MD Vi-DT group who experienced erythema/redness at site of injection and 1(0.33%) in the control group. Three participants (0.40%) in the MD Vi-DT group experienced swelling/induration. One participant (0.33%) in control group experienced fever and one participant (0.17%) in the MD Vi-DT group reported a headache. Only one severe immediate reaction (erythema/redness at site of injection) was reported for age stratum 3 in the MD Vi-DT group (Supplement Table S5).

**Table 4:** Summary of adverse events observed during the study period.

Entire study period	Vi-DT (MD) (N=750)		Vi-DT (SD) (N=750)		Control (N=300)		P-value
	m	n (%)	m	n (%)	m	n (%)	
Immediate Reaction (for 30 minutes after vaccination)	14	13 (1.73%)	7	7 (0.93%)	3	3 (1.00%)	0.3460[1]
Severity:							
Mild	13	12 (1.60%)	7	7 (0.93%)	2	2 (0.67%)	0.3286[1]
Moderate	0	0 (0.00%)	0	0 (0.00%)	1	1 (0.33%)	0.1667[2]
Severe	1	1 (0.13%)	0	0 (0.00%)	0	0 (0.00%)	1.0000[2]
Potentially life threatening	0	0 (0.00%)	0	0 (0.00%)	0	0 (0.00%)	NA
Relatedness:							
Definitely Related	12	11 (1.47%)	6	6 (0.80%)	2	2 (0.67%)	0.3470[1]
Probably Related	2	2 (0.27%)	1	1 (0.13%)	1	1 (0.33%)	0.8190[2]
Possibly related	0	0 (0.00%)	0	0 (0.00%)	0	0 (0.00%)	NA
Unlikely related	0	0 (0.00%)	0	0 (0.00%)	0	0 (0.00%)	NA
Not Related	0	0 (0.00%)	0	0 (0.00%)	0	0 (0.00%)	NA
Solicited AE (During 7 days after vaccination)	413	151 (20.13%)	367	144 (19.20%)	212	75 (25.00%)	0.1026[1]
Severity:							
Mild	330	139 (18.53%)	311	135 (18.00%)	176	68 (22.67%)	0.2005[1]
Moderate	77	37 (4.93%)	51	25 (3.33%)	34	16 (5.33%)	0.2035[1]
Severe	6	4 (0.53%)	5	4 (0.53%)	2	2 (0.67%)	1.0000[2]
Potentially life threatening	0	0 (0.00%)	0	0 (0.00%)	0	0 (0.00%)	NA
Relatedness:							
Definitely Related	199	89 (11.87%)	217	86 (11.47%)	105	44 (14.67%)	0.3393[1]
Probably Related	138	63 (8.40%)	98	54 (7.20%)	66	21 (7.00%)	0.6099[1]
Possibly related	62	24 (3.20%)	47	23 (3.07%)	34	19 (6.33%)	0.0264[1]
Unlikely related	8	4 (0.53%)	2	2 (0.27%)	3	2 (0.67%)	0.6505[2]
Not Related	6	3 (0.40%)	3	3 (0.40%)	4	2 (0.67%)	0.7958[2]
Solicited AEs related to vaccine:	407	148 (19.73%)	364	142 (18.93%)	NA	NA	0.6948[1]
Severity:							
Mild	327	137 (18.27%)	308	133 (17.73%)	NA	NA	0.7881[1]
Moderate	74	36 (4.80%)	51	25 (3.33%)	NA	NA	0.1504[1]
Severe	6	4 (0.53%)	5	4 (0.53%)	NA	NA	1.0000[2]
Potentially life threatening	0	0 (0.00%)	0	0 (0.00%)	NA	NA	NA
Unsolicited AE (During the 4 weeks (28 days) after vaccination)	119	105 (14.00%)	119	97 (12.93%)	50	41 (13.67%)	0.8295[1]
Severity:							
Mild	112	99 (13.20%)	113	93 (12.40%)	47	40 (13.33%)	0.8706[1]
Moderate	7	7 (0.93%)	5	5 (0.67%)	2	2 (0.67%)	0.8174[1]
Severe	0	0 (0.00%)	1	1 (0.13%)	1	1 (0.33%)	0.3056[2]
Potentially life threatening	0	0 (0.00%)	0	0 (0.00%)	0	0 (0.00%)	NA
Relatedness:							
Definitely Related	0	0 (0.00%)	0	0 (0.00%)	1	1 (0.33%)	0.1667[2]
Probably Related	2	2 (0.27%)	2	2 (0.27%)	0	0 (0.00%)	1.0000[2]
Possibly related	1	1 (0.13%)	0	0 (0.00%)	0	0 (0.00%)	1.0000[2]
Unlikely related	7	7 (0.93%)	11	9 (1.20%)	8	6 (2.00%)	0.3634[1]
Not Related	109	96 (12.80%)	106	89 (11.87%)	41	35 (11.67%)	0.8154[1]
Unsolicited AEs related to vaccine:	10	10 (1.33%)	13	11 (1.47%)	NA	NA	0.8261[1]
Severity:							
Mild	8	8 (1.07%)	10	9 (1.20%)	NA	NA	0.8073[1]
Moderate	2	2 (0.27%)	3	3 (0.40%)	NA	NA	1.0000[2]
Severe	0	0 (0.00%)	0	0 (0.00%)	NA	NA	NA
Potentially life threatening	0	0 (0.00%)	0	0 (0.00%)	NA	NA	NA
SAE	3	3 (0.40%)	2	2 (0.27%)	0	0 (0.00%)	0.8489[2]

[Note] MD: Multidose; SD: Single Dose; AE: Adverse Event; SAE; Serious Adverse Event; N: number of total participants; n: number of participants who reported events; m: number of events; %: percentages (100*n/N). NA: Not Applicable.

The p-value was derived from [1] Chi-square test or [2] Fisher's exact test compared among Vi-DT (MD), Vi-DT (SD), and Control.

##### Solicited Adverse Events

A total of 992 solicited AEs were reported in 370 participants within the seven days post-vaccination: 151(20.13%) in MD Vi-DT group, 144 (19.20%) in SD Vi-DT group and 75 (25.00%) of control group (*p*=0.103). The distribution of AEs across age groups was comparable (Supplement Table S5 – S7). The most frequent local solicited AE reported was pain/tenderness at site of injection, which was reported for 89 (8.93%) in the MD Vi-DT group, 88 (8.80%) in the SD Vi-DT group and 110 (11.0%) in the control group. The majority of AEs were mild to moderate and resolved within a few days; 13 (13.1%) solicited AEs reported in 10 participants were classified as severe. There was significantly higher number of participants in the control vaccine group compared to the MD Vi-DT and SD Vi-DT groups who reported swelling or induration (*p*=0.0038) and arthralgia (*p*=0.0107). All severe solicited AEs in all groups resolved within 1-3 days.

##### Unsolicited Adverse Events

A total of 288 unsolicited adverse events were reported from 243 participants within the first month of vaccination: 105(14.00%) in MD Vi-DT group, 97(12.93%) in SD Vi-DT group and 41(13.67%) of control group (*p*=0.829). There was no statistically significant difference in the proportion of participants with unsolicited AEs among the different vaccine groups across the three age strata. Upper respiratory tract infection was the most common unsolicited reaction reported for all age groups occurring in 29(3.87%) of MD Vi-DT group, 38(5.07%) in the SD Vi-DT group and 10 (3.33%) in the control group. Most unsolicited AEs were classified as mild to moderate, two unsolicited AEs – one case of fever each in the SD Vi-DT group and the control group – were classified as severe. Five unsolicited AEs were classified as possibly or probably related in the MD and SD Vi-DT formulations vaccine groups and one was classified as definitely related in the control vaccine group. All unsolicited AEs resolved with no sequelae.

##### Serious Adverse Events

Five SAEs were reported during the 24-week follow-up period, all were judged unrelated to vaccine. The first SAE was a case of pneumonia with suspected COVID-19 which required hospitalization in an 11-month-old infant. Also reported were a case of premature birth at 34 weeks of gestation, seizure disorder in a 1year and 4 months toddler, and dengue fever with suspected COVID-19 in a 1 year and 3 months toddler. There was one death – a case of pulmonary TB confirmed by GeneXpert and pneumonia in a 30 years and 4 months old male adult.

## Discussion

Our findings show that multidose and single dose formulations of Vi-DT typhoid conjugate vaccine are immunologically equivalent at four weeks post vaccination. We also showed that these formulations are safe for use in children and adults for potential application in mass vaccination campaign scenarios or in routine immunization programs like the expanded program of immunization (EPI). The findings further complement existing preclinical and clinical data generated towards licensure and/or prequalification of this promising conjugate vaccine to support WHO's strategy to scale up typhoid vaccination in endemic regions.

There is a need to investigate the impact of different components of a vaccine formulation, for example preservatives in multidose formulations, on safety and immunogenicity of typhoid conjugate vaccines. Such evidence is critical to support the use of alternative dosing formulations to enhance vaccine roll out in endemic and epidemic settings. Multidose vials, in general, sell at a lower per-dose price and occupy less cold-chain capacity than single dose formulations but with potentially higher wastage rates especially for more expensive vaccines. Additionally, Vi-DT multidose formulations would offer a vital opportunity to enhance mass vaccination campaigns in cases of typhoid outbreaks or in high-risk settings of vulnerable communities where larger numbers of people need to be vaccinated in a short period of time. Single dose formulations similarly offer several programmatic benefits including reducing wastage.^23^ Additionally, reducing vaccine wastage could potentially lead to a reduction in other costs related to waste disposal which has likely grown as a significant cost driver over the past two decades. Confirming immune equivalence and safety of the two Vi-DT formulations and availability in alterative formulations would allow for a flexible vaccine roll out informed by the status of typhoid infections and transmission in the specific setting notwithstanding the preference of most manufacturers to produce multidose vials to support mass vaccination campaigns in endemic settings.

There are some limitations that should be considered while interpreting our results. First, due to the Covid-19 pandemic, significant changes had to be made to the enrollment of participants in the different groups, for example adult participants had to be re-assigned to the only safety group. However, this change did not affect our power to address the primary endpoint as more adult participants were enrolled in the immunogenicity group. Secondly, to date, there is no clearly defined correlate of protection of anti-Vi antibody levels against *Salmonella* infection and/or clinical typhoid disease. Serum bactericidal antibody (SBA) levels were shown to poorly correlate with disease and anti-Vi IgG and IgM titers.^26^ Similarly, no significant correlation was observed between the fold change in anti-Vi IgG and IgA and typhoid fever.^27^ Third, the immune equivalence of the two formulations was evaluated only in the adult age group. Even though the immune response of the Vi-DT vaccine has been shown to be robust in children,^20^, ^21^, ^22^^,^^25^ the lack of immune equivalence data for children limits our generalization to all age groups.

The COVID-19 pandemic which peaked in the Philippines during the middle of the enrollment for this trial was a significant challenge that disrupted enrollment and follow up of participants. However, the mitigation strategies that we implemented including enrolment and follow up procedures that comply with the Philippines Government recommendations and that ensured protection of study staff and participants from SARS-CoV-2 infection enabled completion of the study within timelines and adhering to the approved protocol, tenets of the ICH-GCP E2 recommendations and all ethical requirements. Data quality and integrity were confirmed following the stringent quality control and monitoring procedures.

The satisfactory safety profile in this study complements the available evidence on the safety of typhoid conjugate vaccines in persons older than 2 years of age.^12^^,^^28^, ^29^, ^30^ In addition, our findings showed that Vi-DT is safe in infants as young as 6 months, adding to the available evidence reported from India for Vi-TT,^31^ from Philippines and Nepal for Vi-DT^20^, ^21^, ^22^ and from India for Vi-CRM _197_.^32^

Taken together, Vi-DT MD and SD formulations are equivalent in eliciting anti-Vi IgG immune responses in adults and are safe in all age groups including children 6-23 months of age, a group bearing significant typhoid morbidity and mortality. Prequalification of Vi-DT in both formulations will enable inclusion of the vaccine in the routine EPI schedule for children as a single dose formulation while the MD formulation can be used during outbreak settings or vulnerable communities, for example refugee camps and humanitarian settings. We recommend implementation of real-world studies evaluating vaccine effectiveness in programmatic implementation settings and cost-effectiveness evaluation of both Vi-DT formulations.

## Contributors

BTT, TAW, JSY, ZRM, DRK, JHK and SS contributed during the study design. JCC, BTT, CBT, EA, MCY, DRK, HSA, MK, SYY, JP, JSY, JHK, JYL, TAW and SS participated in study implementation; DRK and HYA performed data analysis. BTT and SS drafted the Manuscript. MRC, SC, JP, SKJ and EA took part in study implementation in the field. SYY, JHR, HP, JHS, YL and HK made substantial revisions to the last version of the Manuscript.

## Declaration of interests

JHR, HKP, JHS, YL, and HK are employees of SK Bioscience. JHK is a scientific consultant to SK Bioscience for COVID 19 vaccine research. All remaining authors declare no competing interests.

## Appendix Supplementary materials

Image, application 1

Image, application 2
