# Supplementary material for: A Phase 3, Multicenter, Randomized, Controlled Trial to Evaluate Immune Equivalence and Safety of Multidose and Single-dose Formulations of Vi-DT Typhoid Conjugate Vaccine in Healthy Filipino Individuals 6 Months to 45 Years of Age
Source: Lancet Reg Health West Pac. 2022 May 30;24:100484. doi: 10.1016/j.lanwpc.2022.100484 (PMC9160840; doi:10.1016/j.lanwpc.2022.100484)
Supplement: Supplementary file 1 [file mmc1.pdf]

A phase III, multicenter, observer blind, randomized, controlled study to evaluate immune equivalence of multi-dose formulation against single-dose formulation of Vi-DT Typhoid conjugate vaccine and safety in healthy Filipino participants aged 6 months to 45 years.

| | |
|---|---|
| **Protocol Number:** | **IVI T004** |
| **Sponsor:** | International Vaccine Institute<br>SNU Research Park, 1 Gwanak-ro,<br>Gwanak-gu, Seoul, 08826<br>Republic of Korea |
| **Co-sponsor** | SK bioscience<br>310 Pangyo-ro, Bundang-gu,<br>Seongnam-si, Gyeonggi-do, 13494,<br>Republic of Korea |
| **Funding Agency:** | Bill and Melinda Gates Foundation<br>Seattle, WA, USA |
| **Version Number, Date:** | Version 5.0, 12JUN2020 |

**FOR OFFICIAL USE ONLY**
Information and data included in this document contain privileged and/or proprietary information, which is the property of the International Vaccine Institute, SK bioscience, and participating clinical trial sites in Philippines, and may not be reproduced, published or disclosed to others without written authorization. These restrictions on disclosure will apply equally to all future information, which is indicated as privileged or proprietary.

## TABLE OF CONTENTS


Key Roles ........ **Error! Bookmark not defined.**
List of Abbreviations ........ 5
1 SYNOPSIS ........ 7
2 INTRODUCTION ........ 15
2.1 Background ........ 15
3 Vi-DT CONJUGATE VACCINE ........ 20
3.1 Preclinical Data ........ 20
3.2 Clinical Data ........ 21
3.3 Study Rationale ........ 22
3.4 Potential Risks and Benefits ........ 24
3.4.1 Known Potential Risks ........ 24
3.4.2 Known Potential Benefits ........ 24
4 OBJECTIVES ........ 24
5 STUDY DESIGN ........ 25
5.1 Study Endpoints ........ 25
5.2 Methodology ........ 26
5.2.1 Justification for dose of vi-dt ........ 27
5.3 Measures to Minimize Bias ........ 30
5.3.1 Randomization/Masking Procedures ........ 30
5.3.2 BLINDING ........ 31
5.3.3 Unblinding of Participants ........ 31
6 STUDY AGENT ........ 32
6.1 Study Agent(s) and Control Description ........ 32
6.1.1 Acquisition ........ 32
6.1.2 Formulation, Appearance, Packaging, and Labeling ........ 32
6.1.3 Preparation ........ 36
6.1.4 Dosing and Route of Administration ........ 36
6.1.5 Dose Adjustments/Modifications/Delays ........ 36
6.1.6 Tracking of Dose ........ 36
6.2 Study Agent Accountability Procedures ........ 36
6.3 Standard of Care ........ 37
6.4 Concomitant Medications, Treatments, and Procedures ........ 37
7 STUDY POPULATION ........ 37
7.1 Strategies for Recruitment and Retention ........ 37
7.2 Consent and Assent Procedures and Documentation ........ 38
7.3 Compensation for Participation ........ 39
7.4 Participant Inclusion Criteria ........ 40
7.5 Participant Exclusion Criteria ........ 40
7.6 Study Procedures ........ 41
7.6.1 Screening ........ 41
7.6.2 Enrollment ........ 42
7.6.3 Follow-up Procedures and Visits ........ 43
7.7 Participant Withdrawal or Termination ........ 44
7.7.1 Reasons for Withdrawal or Termination ........ 44
7.7.2 Handling of Participant Discontinuation or Termination ........ 45

7.8 Lost to Follow-Up ....46
7.9 Protocol Deviations ....46
7.10 Protocol Amendments ....47
7.11 Premature Termination or Suspension of Study ....47
7.12 End of Study ....48
8 LABORATORY PROCEDURES/EVALUATIONS ....48
8.1 Specimen Processing, Handling, and Storage ....48
8.2 Specimen Shipment ....48
8.3 Assessment of Immunogenicity ....48
9 ASSESSMENT OF SAFETY ....49
9.1 Safety Assessment ....49
9.1.1 Definition of Adverse Events (AE) ....49
9.1.2 Definition of Serious Adverse Events (SAE) ....50
9.1.3 Definition of Suspected unexpected serious adverse event (SUSAR) ....51
9.2 Classification of an Adverse Event ....51
9.2.1 Severity of Event ....51
9.2.2 Relationship to Investigational Product ....54
9.2.3 Expectedness ....55
9.3 Time Period and Frequency for Event Assessment and Follow-Up ....55
9.4 Reporting Procedures ....56
9.4.1 Adverse Event Recording and Reporting ....56
9.4.2 Serious Adverse Event Reporting ....57
9.4.3 Safety Oversight ....58
10 STUDY MONITORING ....59
11 STATISTICAL CONSIDERATIONS ....59
11.1 Sample Size ....59
11.2 Statistical Analysis Plan ....60
11.3 Statistical Hypotheses ....60
11.4 Analysis Datasets ....61
11.5 Description of Statistical Methods ....62
11.5.1 General Approach ....62
11.5.2 Baseline Descriptive Statistics ....62
11.5.3 Safety Analysis ....62
11.5.4 Analysis of the Primary Immunogenicity Endpoint(s) ....63
11.5.5 Analysis of the Secondary Immunogenicity Endpoint(s) ....63
11.5.6 Adherence and Retention Analyses ....64
11.5.7 Planned Interim Analysis ....64
11.5.8 Additional Sub-Group Analysis ....64
11.5.9 Multiple Comparison/Multiplicity ....64
12 SOURCE DOCUMENTS AND ACCESS TO SOURCE DOCUMENTS ....65
13 DATA HANDLING AND RECORD KEEPING ....66
13.1 Data Collection and Management Responsibilities ....66
13.2 Study Records Retention ....66
13.3 Publication and Data Sharing Policy ....67
14 QUALITY ASSURANCE AND QUALITY CONTROL ....67
15 ETHICS/PROTECTION OF HUMAN PARTICIPANTS ....68
15.1 Regulatory and Ethical Compliance ....68

15.2 Participant and Data Confidentiality ........ 68
15.3. Research Use of Stored Human Samples ........ 69
15.4 Future Use of Stored Specimens ........ 70
16 REFERENCES ........ 71
17 APPENDICES ........ 73
Statement of Compliance ........ 74
Vi-DT Phase II PRIMARY clinical study Report SUMMaRY ........ 78

## LIST OF ABBREVIATIONS

| | |
|---|---|
| AE | Adverse Event |
| ANOVA | Analysis of Variance |
| BMGF | Bill and Melinda Gates Foundation |
| BSA | Bovine Serum Albumin |
| °C | Degree Celsius |
| CI | Confidence Interval |
| CIOMS | Council for International Organizations of Medical Sciences |
| CMP | Clinical monitoring plan |
| CRF | Case Report Form |
| CSR | Clinical Study Report |
| DOB | Date of Birth |
| DSMB | Data Safety Monitoring Board |
| DT | Diphtheria Toxoid |
| EC | Ethics Committee |
| eCRF | Electronic Case Report Form |
| ELISA | Enzyme Linked Immunosorbent Assay |
| EPI | Expanded Program on Immunization |
| FAS | Full Analysis Set |
| GCP | Good Clinical Practices |
| GMT | Geometric Mean Titer |
| HIV | Human Immunodeficiency Virus |
| IB | Investigator's Brochure |
| ICF | Informed Consent Form |
| ICH | International Council for Harmonization |
| IgG | Immunoglobulin G |
| IL | Interleukin |
| IRB | Institutional Review Board |
| IVI | International Vaccine Institute |
| MD | Multi Dose |
| LAR | Legally Acceptable Representative |
| M-ITT | Modified Intention-To-Treat |
| MOP | Manual of Procedures |
| MR | Measles Rubella |
| N | Number |
| PBS | Phosphate Buffered Saline |
| PE | Phenoxy ethanol |
| PFDA | Food and Drug Administration Philippines |
| PHL/PH | Philippines |
| PI | Principal Investigator |
| PP | Per Protocol |
| QA | Quality Assurance |
| QC | Quality Control |

| | |
|---|---|
| SAE | Serious Adverse Event |
| SAP | Statistical Analysis Plan |
| SD | Single Dose |
| SI | Site investigator |
| SOE | Schedule of Events |
| SOP | Standard Operating Procedure |
| SUSAR | Suspected Unexpected Serious Adverse Reaction |
| UPT | Urine Pregnancy test |
| Vi-DT | Diphtheria Toxoid Conjugated Vi-Polysaccharide Vaccine |
| Vi-PS | *Salmonella* Typhi Capsular Polysaccharide Vaccine |
| Vi-rEPA | *Pseudomonas aeruginosa* exotoxin A Conjugated Vi-Polysaccharide Vaccine |
| Vi-TT | Tetanus Toxoid Conjugated Vi-Polysaccharide Vaccine |
| WHO | World Health Organization |

# 1 SYNOPSIS

| | |
|---|---|
| **Name of the Sponsor:** International Vaccine Institute (IVI) | |
| **Name of Investigational Product:** Typhoid Conjugate Vaccine (Vi-DT) Multi dose (MD), Single dose (SD) | |
| **Name of Active Ingredients:** Diphtheria Toxoid Conjugated Vi-Polysaccharide Typhoid Vaccine | |
| **Title of Study:**<br><br>A phase III, multicenter, observer blind, randomized, controlled study to evaluate immune equivalence of multi-dose formulation against single-dose formulation of Vi-DT Typhoid conjugate vaccine and safety in healthy Filipino participants aged 6 months to 45 years. | |
| **Protocol Number:** IVI T004 | |
| **Study Sites:** Study participants may be enrolled from the following sites;<br><br>• University of the Philippines Manila - National Institutes of Health, Ermita Manila<br>• Lingga Health Research Center Calamba, Laguna<br>• Putatan Research Center, Putatan, Muntinlupa City<br>• Magcase Health Center, San Pablo City, Laguna | |
| **Study Period (years/months)**<br><br>Estimated date first participant enrolled: Jan 2020<br>Estimated date last participant enrolled: May 2020<br>Estimated duration of the trial: 18 months | **Phase of development:** III |
| **Study Hypothesis**<br><br>The scientific rationale described in this protocol, seeks to establish immune equivalence of a multi-dose formulation of Vi-DT (MD) compared to single dose formulation of Vi-DT (SD) in a subset of study participants of 18 to 45 years of age. The clinical research study is intended to assess immune equivalence for adult age stratum at 4 weeks post vaccination. The sample size calculation is based on the hypothesis of the immunogenicity endpoint which has been further increased to include safety data base from 6 months to less than 18 years age strata. Safety assessment of a single and multi-dose formulations of Vi-DT and control (Meningococcal | |

conjugate vaccine) in age 6 months to 45 years will be evaluated by analyzing safety data descriptively at 4 and 24 weeks post injection.

In summary, safety population includes study participants from 6 months to 45 years old while the immunogenicity subpopulation is only in adults from 18 to 45 years of age.

**Objectives**
**Primary**

- Demonstrate the immune equivalence as measured by anti-Vi IgG Geometric Mean Titer (GMT) of multi dose formulation against single dose formulation of Vi-DT (18-45 year age stratum), at 4 weeks after a single dose.

**Secondary**

- Demonstrate the immune equivalence as measured by seroconversion rates of anti-Vi IgG antibody titres of multi dose formulation against single dose formulation of Vi-DT vaccine (18-45 year age stratum) at 4 weeks after a single dose.
- Describe safety profile in all age strata combined (age 6 months - 45 years old) and in each age stratum, at 4 weeks after a single dose of SD/MD formulation/control (Meningococcal Conjugate Vaccine).

**Methodology**

This is a multicenter, randomized, observer-blinded, controlled, immune equivalence study of a multi-dose (MD) formulation with 2PE preservative of SK bioscience Vi-DT compared to single dose (SD) formulation without preservative of SK bioscience Vi-DT in participant (6 months - 45 years) including safety population.The vaccines will be administered to 1,500 healthy participants of 6 months to 45 years of age and followed up for 24 weeks after the injection for safety. Adult participants* (N=500) will be followed up for immunogenicity at 4 weeks and all participants till 24 weeks for safety post single dose of either MD & SD formulations. Three hundred (300) healthy participants will be given control vaccine (locally available licensed Meningococcal conjugate vaccine) to check the background safety events. The primary objective is to demonstrate the equivalence of immunogenicity as measured by anti-Vi IgG GMT titer at 4 weeks after a single dose of MD/SD formulation in adults. The secondary objective is to demonstrate the equivalence of immunogenicity in terms of seroconversion rates as measured by anti-Vi IgG ELISA antibody titers, at 4 weeks after a single dose of MD/SD formulation in adults. A descriptive evaluation of safety at 4 and 24 weeks post single dose of (SD/MD/Meningococcal vaccine), will be performed. The Vi-DT vaccine from both MD & SD formulations will be administered as a single dose of 25 µg/0.5 mL.

Eligible participants enrolled into the study will be randomized into one of the three study groups within each age stratum of 6 months to less than 2 years, 2 to less than 18 years, and 18 to 45 years. Participants will be observed at the study site for 30 minutes after vaccination for safety

assessment. Solicited adverse events will be recorded on a diary card during 7 days after vaccination. Unsolicited adverse events will be recorded during the 4 weeks after vaccination. Serious adverse events will be recorded during the entire study period. With the exception of designated study site personnel responsible for vaccine administration, site investigators, study nurse, and those assessing clinical outcomes, and data analysts will be blinded to vaccine allocation until data base lock for the final analysis.

Blood samples will be collected at baseline prior to vaccination and at 4 weeks post vaccination from adults (18-45 years) for immunogenicity assessment

*Due to city lock down those subjects whose blood sample could not be collected on V4, their post- vaccination blood sample would not be collected when lock down is lifted. This will be consider as deviation and will be informed to respective ethics committee.

**Estimated Number of participants to Enroll**

A total of 1800 participants aged 6 months to 45 years will be enrolled in this study. 1500 participants will be randomized equally into the first 2 groups of 750 participants each and third group with 300 participants. Each group shall be divided into age stratum of 6 months to less than 2 years, 2 to less than 18 years, and 18 to 45 years. Participants in the first group will receive a single dose from multi-dose formulation, while the second group will receive the single dose from single dose formulation of Vi-DT and third group will receive single dose of locally available Meningococcal conjugate vaccine .

**Criteria for Inclusion/Exclusion**

**Inclusion Criteria**

In order to be eligible to participate in this study, any individual must meet the following criteria:

1. Healthy participants 6 months to 45 years of age at enrollment
2. Participants/Parent(s)/LAR who have voluntarily given informed consent/assent
3. Participants/Parent(s)/LAR willing to follow the study procedures of the study and available for the entire duration of the study

**Exclusion Criteria**

An individual who meets any of the following criteria will be excluded from participation in this study:

1. Child with a congenital abnormality
2. Participant who has already received meningococcal conjugate vaccine
3. Participants concomitantly enrolled or scheduled to be enrolled in another trial
4. Known history of immune function disorders including immunodeficiency diseases (Known HIV infection or other immune function disorders)

5. Chronic use of systemic steroids (>2 mg/kg/day or >20 mg/day prednisone equivalent for periods exceeding 10 days), cytotoxic or other immunosuppressive drugs
6. Receipt of blood or blood-derived products in the past 3 months
7. Participant with a previously ascertained or suspected disease caused by *S. Typhi* (confirmed either clinically, serologically or microbiologically)
8. Participant who has had household contact with and/or intimate exposure to an individual with laboratory-confirmed *S. Typhi*
9. Individual who has previously received a typhoid vaccine
10. Participant who has received other vaccines from 1 month prior to test vaccination or planned to receive any vaccine within 1 month (except a measles containing vaccine as per government vaccination campaign)
11. Known history or allergy to vaccines or other medications
12. History of uncontrolled coagulopathy or blood disorders
13. Any abnormality or chronic disease which in the opinion of the investigator might be detrimental for the safety of the participant and interfere with the assessment of the study objectives
14. Any female participant who is lactating, pregnant* or planning for pregnancy during the course of study period
15. Participants/Parent(s)/LAR planning to move from the study area before the end of study period
16. As per Investigator’s medical judgement individual could be excluded from the study in spite of meeting all inclusion/exclusion criteria mentioned above

**Temporary Contraindication**

1. Acute illness, in particular infectious disease or fever (axillary temperature ≥37.5°C), within three days prior to enrollment and vaccination
   a. These individual could be rescreened upon resolution of the above said conditions

*UPT is necessary for all female participants of childbearing age from menarche.

**Investigational Product, Dosage and Mode of Administration:**

**Test Vaccine 1**

Vi polysaccharide typhoid vaccine conjugated with Diphtheria toxoid protein (Vi-DT), manufactured by SK bioscience (Republic of Korea).

- Dose formulation: 25 µg Vi polysaccharide /0.5 mL, presented in Type I glass vial (multi dose Vi-DT with preservative 2 PE)
- Mode of Administration: 0.5 mL by intramuscular injection in the left anterolateral thigh or left arm deltoid region in participants below 2 years of age, less dominant arm deltoid region in age group 2 to 45 years
- Storage Conditions: +2 to +8°C

**Test Vaccine 2**

Vi polysaccharide typhoid vaccine conjugated with Diphtheria toxoid protein (Vi-DT), manufactured by SK bioscience (Republic of Korea).

- Dose formulation: 25 µg Vi polysaccharide /0.5 mL, presented in Type I glass vial (single dose Vi-DT without any preservative)
- Mode of Administration: 0.5 mL by intramuscular injection in the left anterolateral thigh or left arm deltoid region in participants below 2 years of age, less dominant arm deltoid region in age group 2-45 years
- Storage Conditions: +2 to +8°C

**Control Vaccine**

Locally available Meningococcal conjugate vaccine

- For participant ≥ 1 year one dose of locally licensed Meningococcal conjugate vaccine will be administered
- For participants 6 months to 1 year one dose of locally licensed Meningococcal conjugate vaccine will be administered during the study and the next dose will be provided after the study unblinding at the completion of 6 months follow up of last subject.

**Group Allocation**

| Group of Vaccinees and age group | Age Strata | N* | D0 |
|---|---|---|---|
| **Group A**<br>750 participants<br>(6 mo - 45 yrs) | 18-45 yrs | 250 | (25 µg 0.5 mL)<br>(Vi-DT)<br>Multi dose formulation |
| | 2- <18 yrs | 250 | |
| | 6 mo-<2 yrs | 250 | |
| **Group B**<br>750 participants<br>(6 mo - 45 yrs) | 18-45 yrs | 250 | (25 µg 0.5 mL)<br>(Vi-DT)<br>Single dose formulation |
| | 2- <18 yrs | 250 | |
| | 6 mo-<2 yrs | 250 | |
| **Group C**<br>300 participants<br>(6 mo - 45 yrs) | 18-45 yrs | 100 | Control<br>(Meningococcal Conjugate vaccine) |
| | 2- <18 yrs | 100 | |
| | 6 mo-<2 yrs | 100 | |

* Age Strata wise number of enrolled subjects may be adjusted during the actual enrollment.

**Age Strata reassignment (Stratawise Enrollment number of subject)**

The initial enrollment plan required an equal number of subjects enrolled from each age stratum and the current plan allows for the adjustment of the required number of subjects in each stratum during the enrollment period.

The post-dose blood sample could not be collected from the enrolled adults stratum due to COVID-19 lock down situation in place since 13MAR2020 in the Philippines leading to the decision to redistribute more subjects to the adults stratum from the two other age strata (Strata 2 and Strata 3). The reason of the current plan is to meet the required power for immune equivalence among adults.

## Criteria for Evaluation

### Primary Endpoints

- Geometric Mean Titers (GMT) of anti-Vi IgG at 4 weeks (28 days) after vaccination of Vi-DT(MD) vs Vi-DT (SD) [18-45 year age stratum]

### Secondary Endpoints

- Seroconversion rates (defined as a 4-fold increase of serum anti-Vi IgG antibody titer from baseline) of anti-Vi IgG antibody titers at 4 weeks (28 days) after vaccination with Vi-DT(MD) vs Vi-DT (SD) compared to baseline (D0) [18-45 year age stratum]

- **Comparison of safety of V-DT compared to control:** Vi-DT MD and SD will be compared separately to control vaccine and will be compared by age strata at 4 weeks after vaccination.
  - Local and systemic solicited adverse events during the 7 days after vaccination
    - Solicited local reactions at the site of injection: pain, tenderness, erythema/redness, swelling/ induration, pruritus
    - Solicited Systemic reactions (adapted to each age group): fever, lethargy, irritability, nausea/vomiting, arthralgia, diarrhea, drowsiness, loss of appetite, chills, headache, fatigue, myalgia and persistent crying
    - Unsolicited adverse events during 4 weeks (28 days) after vaccination
    - Serious Adverse Events during the entire study period

**Statistical Considerations**

The sample size of two Vi-DT groups is decided based on the immunogenicity equivalence and safety data requirement. The immunogenicity subset in adult participants, N=250 per group, will provide 94% power to show equivalence of geometric mean titres (GMT) of anti-Vi IgG at 4 weeks (28 days) after vaccination of Vi-DT(MD) and Vi-DT (SD), with the equivalence margin of 0.67 to 1.5 following WHO recommendation per (WHO TRS 924). Coefficient of variation (CV) of immunogenicity titre is conservatively assumed as 2.0 based on data from IVI T001 (i.e., Vi-DT phase 1 in children, adolescents, and adults in the Philippines) and IVI T002 (i.e., Vi-DT Phase 2 in infants/toddlers in the Philippines) studies with type 1 error rate of 0.05 and 10% drop out rate are also assumed. This sample size of N=250 will provide 95% power, for equivalence tests of seroconversion rate between two formulations of Vi-DT with equivalence margin of [-10%, 10%]. In this calculation, 90% of sero-conversion rate in Vi-DT is assumed (based on IVI T001) with type 1 error rate of 0.05. The sample size of N=300 control (Meningococcal vaccine) is calculated to observe at least one events with 1% of the upper limit of 95% CI for any adverse event of incidence according to rule of three.

**<u>Primary Comparison</u>**

To demonstrate equivalence of two Vi-DT formulations (MD vs. SD)

- Anti-Vi IgG GMT at 4 weeks (28 days) post of Vi-DT (MD) is equivalent to GMT of Vi-DT(SD) in adults using equivalence margin of GMT ratio of [0.67, 1.5]

If the 95% confidence interval of the ratio of GMT estimate of Vi-DT(MD) over GMT of Vi-DT(SD) is located within the bounds of 0.67to 1.5, then Vi-DT (MD) is equivalent to Vi-DT (SD) in terms of GMT of anti-Vi IgG with significance level of 0.05.

**<u>Secondary Comparisons</u>**

- Seroconversion rates of anti-Vi IgG ELISA antibody titres at 4 weeks (28 days) from baseline(D0) of Vi-DT(MD) is equivalent to seroconversion rate at 4 weeks of Vi-DT(SD) using equivalence margin of 10%

If the 95% confidence interval of the estimate of difference of seroconversion rate between Vi-DT (MD) and Vi-DT (SD) at 4 weeks (Day 28) is located within the bounds -10% to 10%, then Vi-DT (MD) is equivalent to Vi-DT (SD) in terms of sero-conversion rate, which is defined as 4 fold increase of anti Vi IgG from baseline with significance level of 0.05.

The following safety endpoints will be summarized by each formulation and overall and within each age stratum.

- Frequency of local and systemic solicited adverse events during the 7 days after each dose
  - Solicited local reactions at the site of injection: pain, tenderness, erythema/redness, swelling/ induration, pruritus
  - Solicited Systemic reactions (adapted to each age group): fever, lethargy, irritability, nausea/vomiting, arthralgia, diarrhea, drowsiness, loss of appetite, chills, headache, fatigue, myalgia and persistent crying

- Frequency of unsolicited adverse events during 4 weeks (28 days) after vaccination

- Frequency of Serious Adverse Events during the entire study period

# 2 INTRODUCTION

## 2.1 BACKGROUND

Typhoid fever is one of the most common causes of bacteremia in several low-and-middle-income countries (LMIC) and has been estimated to cause 11- 21 million cases and 145,000-161,000 deaths per year [1]. Typhoid fever is more common in children and young adults than in older people [2]. Worldwide, typhoid fever is most prevalent in impoverished areas that are overcrowded with poor access to sanitation. Incidence estimates suggest that south-central Asia, Southeast Asia, and southern Africa are regions with high incidence of *S*. Typhi infection (more than 100 cases per 100,000 person years) [3-5]. Other regions of Asia and Africa, Latin America, the Caribbean, and Oceania have a medium incidence of 10 to 100 cases per 100,000 person years. These estimates, however, are limited by lack of consistent reporting from all areas of the world and are based on extrapolation of data across regions and age groups. Recent data from Africa have revealed that several countries in Eastern and West Africa have rates >100 per 100,000 [6].

*Etiological agent*

Typhoid fever is caused by *Salmonella enterica* serovar Typhi (*S*. Typhi). It is a rod-shaped gram-negative facultative anaerobe bacterium belonging to the Enterobacteriaceae family. Among more than 2,300 closely-related Salmonella serovars recognized, *Salmonella enteritica* serotype Typhi and *Salmonella enterica* serotype Paratyphi A, B & C are pathogenic exclusively for humans. Infection therefore implies contact with infected person or use of contaminated food or water. Non typhoidal salmonella (such as *Salmonella enteritidis* and *Salmonella typhimurium* may also cause sever illness consistent with typhoid fever [7]. Salmonella possesses a flagellar antigen (H), somatic (O) and a surface antigen (Vi). The Vi capsular antigen is a superficial overlying antigen. It is present in a few serovars, the most important of which is *Salmonella enteritica* serotype Typhi but is also present in *Salmonella enteritica* serotype Paratyphi C and *Salmonella dublin*.

*Clinical presentation*

Typhoid fever is one of the most common causes of bacteremia in many developing countries [4]. The clinical feature of typhoid fever is that of a sub-acute systemic infection. Classic reports describe the characteristic stages of typhoid fever in untreated individuals with rising fever and

bacteremia. However presentation is variable ranging from mild fever to more severe forms such as toxic shock. General symptoms include high grade fever (40°C) lasting for more than 3 days, profuse sweating, chills, abdominal pain, altered bowel functions, malaise, myalgia, anorexia, intestinal bleeding and perforation [8]. In a small percentage of cases, the bacteria may also colonize the gall bladder, leading to a chronic carrier state. Although the disease is known widely, typhoid fever is still often confused with other acute febrile illnesses such as malaria, typhus and dengue fever, even with the use of laboratory diagnosis.

*Pathogenesis*

Susceptible human hosts are infected upon consumption of food or water contaminated with *S*. Typhi. Inside the small intestine, *S.* Typhi attach to epithelial cells, penetrates the mucosal epithelium to reach the lamina propria through enterocytes and M cells, the domelike epithelial cells that cover Peyer's patches. In the lamina propria, *S*. Typhi triggers an influx of macrophages and dendritic cells that ingest the bacilli but do not generally kill them. Some remain within macrophages of the small intestinal lymphoid tissue. Other typhoid bacilli are drained into mesenteric lymph nodes where there is further multiplication and ingestion by the macrophages. Eventually, there is a release of tumor necrosis factor-alpha, interleukin-2 (IL-2), IL-6, and other inflammatory cytokines by the mononuclear cells. After reaching the blood circulation via the thoracic duct, the bacteria are filtered from the circulation and sequestered inside the phagocytic cells of the liver, spleen, and bone marrow [9]. Replication within the endothelial system is the hallmark of typhoid fever and is responsible for the clinical finding of prostration, generalized sepsis and hepato-splenomegaly. Some individuals will contain the orgamnism within the gastrointestinal system and do not become systematically ill but have persistent *S.* Typhi carriage [10].

*Typhoid control and prevention*

Most of the typhoid cases are effectively treated with antibiotics, although the case fatality rate remains at about 1%. Improvement in sanitary infrastructures and implementation of hygienic practices can reduce the typhoid disease burden as seen in most developed countries. However, the development of adequate infrastructures for improved water and sanitation requires large and long-term investments, and is therefore a distant goal for impoverished populations. Instead, increased population and limited opportunities in rural areas has resulted in urbanization and increased population density, the major risk factor for typhoid transmission. Basic health education such as hand washing and proper food handling is also known to be

effective in reducing typhoid fever. Although typhoid fever can be effectively treated with antibiotics, growing rates of antibiotic resistance in many countries are making this treatment option increasingly more difficult and costly.

Though a vaccine against typhoid fever was developed and used in the early 20th century, typhoid vaccine development received attention in the early 1960s, when *S.* Typhi strains resistant to chloramphenicol were isolated. As a result, among many candidates, two vaccines, one oral, and one injectable, were licensed in 1990. Today, there are enough evidences that typhoid fever vaccines are efficacious, effective under public health conditions, and have an impact on the incidence for the benefit of larger population. WHO has recommended that countries consider the use of typhoid vaccines for high-risk groups and populations, and for outbreak control [11]. It is therefore essential to consider a comprehensive approach that combines targeted vaccination of high-risk populations as a short- to medium-term prevention measure, along with longer term solutions of water and sanitation improvements and improved living standards [12]. In endemic countries, control of typhoid would require implementing immunization for young children and incorporating typhoid vaccine in the Expanded Program on Immunization (EPI).

*Live, Oral Ty21a Vaccine*

The Ty21a vaccine consists of a mutant strain of Salmonella Typhi Ty2 that was isolated after chemical mutagenesis and has a galE- and Vi-negative phenotype. It is supplied in enteric -coated capsules, or Liquid suspension (lyophilized vaccine + buffer mixed with water upon use). Immune response to the vaccine starts 14 days after vaccination, which is mediated by mucosal (IgA), serum (IgG), and cell-mediated antibodies. The vaccine has showed no booster effect. It has shelf life of 14 days at +25°C.

The overall protective efficacy for a three-dose regimen ranged between 67% and 80% in large-scale efficacy trials, conducted in 1980s in Chile [13]. The most common adverse events reported with Ty21a were mild and transient gastrointestinal disturbances, followed by general symptoms such as fever. This vaccine is licensed for use in persons 2 years and above for the liquid formulation and 5 years and older for the capsule formulation.

*Parenteral Vi Polysaccharide Vaccine*

The parenteral subunit Vi polysaccharide vaccine (ViPS) was developed from wild type *S.* Typhi

strain Ty2 on the basis of non-denatured purification of the Vi polysaccharide at the National Institute of Health (US). The ViPS vaccine is given as a single dose and was found to confer, overall, 64–72% protection for 17–21 months and 55% over 3 years [14]. The ViPS vaccine is well tolerated and safe. The most common side effects are pain, redness and induration at injection site, and fever. The Vi capsular polysaccharide synthesized by *S*. Typhi is an important virulence determinant and the ability to produce antibodies against Vi is a critical component in the host's defense against infection by *S*. Typhi. ViPS vaccine was found to be poorly immunogenic in children 2-5 years and not immunogenic in children < 2 years of age. This vaccine continues to be the most common vaccine in use in high endemic countries and was systematically used in routine public health programs in China, Vietnam and Nepal, The vaccine is widely available in the private market in China, India, Pakistan and many other endemic countries. Few counties such as Sri Lanka use this vaccine in their public health program through targeted approach; otherwise, no other country adopted the vaccine in their immunization program.

*Typhoid conjugate vaccines*

The scientists at the US National Institute of Child Health and Disease (NICHD) have developed the method that used the heterobifunctional cross-linking reagent, N-succinimidyl-3-(2-pyridyldithio)-propionate (SPDP) or adipic acid dihydrazide (ADH) as linkers to bind Vi to proteins. Using a nontoxic recombinant protein that is antigenically identical to *Pseudomonas aeruginosa* exotoxin A as a carrier protein, the resultant conjugates (Vi-rEPA) were more immunogenic in mice and juvenile Rhesus monkeys than the Vi alone. In contrast to the T-independent properties of the Vi alone, conjugates of this polysaccharide with several medically relevant proteins induced booster responses in mice and in juvenile Rhesus monkeys. This synthetic scheme was reproducible, provided high yields of Vi-protein conjugates, and was applicable to several medically relevant proteins such as diphtheria and tetanus toxoids [15]. In a randomized, vaccine-controlled study of infants in Vietnam, Vi-rEPA was safe, elicited protective levels of IgG anti-Vi, and was compatible with EPI vaccines. These data show that Vi-rEPA can be added to the routine immunization of infants in countries where typhoid fever is prevalent [16].

The Novartis Vaccines Institute for Global Health, Siena, Italy, is developing a typhoid conjugate vaccine (Vi-CRM197) using Vi from *Citrobacter freundii* WR7011 conjugated to the, CRM197, a non-toxic mutant of the diphtheria toxin [17]. Phase I and II clinical trials were conducted in

European adults. In the phase I trial, single dose of Vi-CRM197 was compared with Typherix® in 50 European volunteers between 18 to 40 years of age. Phase II trial was a dose-ranging design (12.5, 5.0 or 1.25 µg) with 88 European participants between 18 to 40 years of age in which all Vi-CRM197 doses were at least as immunogenic as unconjugated Vi [18]. Recently, phase II studies have been completed in India, Pakistan, and The Philippines in four different age-groups: 18 to 45 years; 24 to 59 months; 9 to 12 months; and infants aged 6 weeks with each group having 40 participants. Novartis Vaccines Institute for Global Health (NVGH, now Scalvo Behring Vaccines Institute for Global Health, GSK company) since then has transferred the technology to Biological E, Hyderabad, India.

With the technology initially transferred from US NIH, Biomed Pvt Ltd. in India developed a conjugate vaccine using Tetanus Toxoid as the carrier protein. This product was tested in a clinical trial in 169 participants > 12 weeks with a comparison group (Vi) of 37 children > 2 years [19]. The results from this study were compared with the NIH study in Vietnam and it was reported that there was four fold or greater rise in antibody titer of each group on ELISA which was statistically equivalent to Vi-rEPA. Based on the results of this study, this product was submitted for licensure and was licensed for more than 3 months of age in 2008 in India.

Similarly, Bharat Biotech in Hyderabad, India also developed typhoid conjugate vaccine using Tetanus Toxoid as the carrier protein with Vi polysaccharide. This vaccine was tested in children (2 to 17 years) for safety, immunogenicity and dose ranging (15 µg versus 25 µg/0.5 mL). There was no significant difference between two doses of 25 µg/ 0.5 mL and two doses of 15 µg/0.5 mL. In the next clinical trial, comparative assessment of the immunogenicity of Vi-TT versus the polysaccharide vaccine was done in 981 participants (6 months to 45 years old). The investigators found 4-fold seroconversion rates in each treatment arm at 6 weeks post vaccination [20]. After 2 years of follow-up, the anti-Vi titers were maintained seroprotective titers in the study arm as compared to comparator arm. Based on these results, Bharat Biotech received marketing authorization for Typbar-TCV in India in 2013 as a single dose indication for all aged 6 months and above. WHO pre-qualification was awarded to Bharat Biotech for Typbar-TCV in January 2018.

Currently 3 types of typhoid vaccines are licensed for use: (i) typhoid conjugate vaccine (TCV); (ii) unconjugated Vi polysaccharide (ViPS); and (iii) live attenuated Ty21a vaccines. The second and third types have been recommended by WHO since 2008 for the control of typhoid in

endemic and epidemic settings. The recommendation for TCV came through the new WHO position paper in March, 2018 [11].

# 3 VI-DT CONJUGATE VACCINE

The Vi-DT Vaccine is a conjugate typhoid vaccine in which purified Vi polysaccharide derived from Salmonella Typhi C6524 is conjugated to Diphtheria Toxoid (DT) as the carrier protein. The Vi-DT vaccine to be used in this trial contains 25 µg/0.5mL of Typhoid antigen (Vi) in the form of Vi-Diphtheria Toxoid conjugate, presented in a Type I glass vial. Both single dose and multi dose formulation use the same type of vial. In the multi dose Vi-DT presentation, preservative 2 PE is added where the single dose Vi-DT does not have preservative 2 PE.

## 3.1 PRECLINICAL DATA

Pre-clinical immunogenicity and toxicity studies were conducted to assess immunogenicity and ensure safety of Vi-DT in animal models. A first immunogenicity study of Vi-DT was conducted in mice. Typhoid Kovax (Vi-polysaccharide typhoid vaccine) marketed by Korea Vaccine Co., Ltd. was used as a comparator. All animals that received Vi-DT showed higher immune responses than those elicited by the comparator. Serum antibody titers at 6 weeks were higher than at 2 weeks, demonstrating a booster effect. The antibody titers were maintained up to 10 weeks. SK bioscience also used guinea pigs and rabbits for immunogenicity testing. The result showed that Vi-DT elicited higher immune responses than those elicited by the comparator in both animal species.

Toxicology studies for Vi-DT (single dose formulation - SD) with single and repeat dose study in mice, respiratory system toxicity in mice and cardiovascular toxicity in female beagle dogs were done at MPI Research (Mattawan, MI, USA). Study results indicated that both single dose and repeated dose (intramuscular doses on days 1, 15, 29 and 43) of Vi-DT to mice did not result in mortality, clinical or macroscopic observations, or elicit any changes in body weight, food consumption, neurobehavioral measures, or respiratory function. Also respiratory system toxicity and cardiovascular toxicity studies in mice and beagle dogs did not indicate any toxicity with Vi-DT.

Toxicology studies (i.e., repeat dose IM toxicity study in mice, local tolerance study in rabbit,

and developmental toxicity study in rabbit) for Vi-DT (multi dose formulation - MD) have been performed. The multi-dose formulation of Vi-DT conjugate with 2-Phenoxyethanol (2-PE) to mice (conducted in Charles River, Mattawan, MI 49071, US) did not result in test article related deaths, clinical or macroscopic observations, or elicit any changes in body weight, food consumption, neurobehavioral measures, or respiratory function. Although there was degeneration of sciatic nerve axonal/myelin in test article and vehicle, it may represent a local irritant effect of vehicle (2-PE). Through a local tolerance of test article to rabbits (conducted in Nonclinical Research Institute, Chemon Inc.,Yongin-si, Gyeonggi-do, Republic of Korea) concluded that intramuscular dose of test article did not result any significant difference in irritation or inflammation at injection sites with negative control. Intramuscular administration of test article was well-tolerated in rabbits. Additionally, developmental toxicity study in rabbits (Study ongoing in Charles River, Mattawan, MI 49071, US) will be further added in early 2020.

## 3.2 CLINICAL DATA

A first-in-human Phase I trial was conducted in the Philippines to assess the safety and immunogenicity of Vi-DT Conjugate Vaccine compared to Vi-Polysaccharide (Typhim Vi®, Sanofi Pasteur) Typhoid Vaccine in healthy Filipino adults and children. The protocol was approved by the Research Institute of Tropical Medicine (RITM) and IVI IRBs and by the Philippines FDA. Informed consent was obtained from all participants.

A total of 144 participants were recruited in Manila, Philippines, and randomized equally (N=72 in each group) to Test (Vi-DT) and Comparator (Typhim Vi®) group within each stratum of adults, adolescents and young children (N=48 in each stratum). There was no significant difference in age and gender among test and comparator group. Male and female participants were 66% and 34%, respectively. The median age was 26 years (18-45) in adults, 11 years (6-16 years) in adolescents and 4 years (2-5 years) in children [21].

No SAE was reported in either group. No participant was discontinued from the study due to AE. All solicited and unsolicited AEs were mild or moderate in both arms with the exception of a 4-year old girl, in Test group with grade 3 fever that resolved without sequel. The proportions of participants with solicited AEs in Test and Comparator groups were respectively 54.17% and 50% in adults, 37.5% and 45.8% in adolescents and 25% and 25% in children. The proportions of participants with unsolicited AEs in Test and Comparator groups were respectively 50% and

45.8% in adults, 37.5% and 45.8% in adolescents, and 79.17% and 70.83% in children. The majority of solicited AEs in adults were pain, tenderness and headache; in adolescents pain and tenderness; and in children pain and fever [21].

All participants in Test group showed seroconversion (defined as 4-fold increase of anti-Vi IgG titer) after the first and second doses while 97% of participants showed seroconversion in Comparator group. Test group showed about 4-fold higher GMT than in the Comparator group. SBA seroconversion rates were significantly higher in the Test group than in the Comparator group post first and second doses (71% vs. 52.17% and 70.4% vs. 51.39%, respectively). SBA GMT were also significantly higher in the Test group than in the Comparator group post first and second doses (526.56 vs. 271.26 and 586.5 vs. 222.97, respectively). Anti-DT responses were significantly higher in the Test group than in the Comparator group with a 26-fold rise post first dose compared to baseline values in the Test group while a 0.93-rise was observed in the Comparator group [21].

A Phase II clinical trial is being conducted in the Philippines to assess the safety and immunogenicity of Vi-DT Conjugate in healthy Filipino infants and toddlers 6-23 months of age at the time of 1 month following the first vaccine dose. The protocol was approved by the RITM and IVI IRBs and by the Philippines FDA. Informed consent was obtained from all participants. Results from the trial are available as Interim report in the the attached appendix  ii.

## 3.3 STUDY RATIONALE

The Vi capsular polysaccharide synthesized by *S*. Typhi is an important virulence determinant and the ability to produce antibodies against Vi is a critical component in the host's defense against infection by *S.* Typhi. Vaccination with Vi polysaccharide has been shown to protect individuals from typhoid fever but Vi vaccine has a number of limitations. Vi is poorly immunogenic and revaccination does not elicit an anamnestic response [22]. There is increasing evidence of S. typhi infection in younger children supporting the need for vaccinating children against typhoid in the first year of life [23]. However, the response to Vi polysaccharide in children under two years of age is poor and consequently Vi vaccines are not licensed for use in this at risk age group. The limitations of Vi vaccines can be overcome by conjugation of the Vi to a carrier protein. Immune responses to bacterial capsular polysaccharides are generally T-

cell independent and lack affinity maturation, poor antibody subclass switching and the inability to generate memory. Conjugation of the polysaccharide to a protein carrier converts the immune response to T-cell dependent, which is characterized by affinity maturation, subclass switching and induction of memory.

Since the major burden of typhoid fever is borne by pre-school, school and young children [5, 11] and increasing evidence of significant burden under the age of 2 years [24] suggests the urgent need for improved typhoid vaccines in terms of better efficacy and long term immune sustainability. Availability of the locally licensed TCV vaccines in few endemic countries and recent WHO pre-qualification of Typbar TCV is a major step forward in this direction. Recent availability of Gavi funds may help in overcoming funds crunchs and ensuring incorporation of TCV in vaccination programs for Gavi eligible countries. To meet the global demand of TCV and to make it more affordable there is a need for more TCV vaccine candidates in market. IVI and SK bioscience with funding support from BMGF are developing a typhoid conjugate vaccine composed of Vi polysaccharide conjugated to diphtheria toxoid (Vi-DT).

Most of typhoid conjugate vaccines tested in humans were administered at the dose of 25 µg. Vi-DT typhoid conjugate vaccine developed by SK bioscience was also tested at the dose of 25 µg in a Phase I and II clinical trials conducted in the Philippines.

Phase II trial in Filipino infants and toddlers 6-23 months of age is going on and post dose 1 results establishes the safety and immunogenicity of the Vi-DT in below two years age group (6-23 months). For the study results, refer to Appendix ii.

Phase I and II studies have demonstrated the safety and immunogenicity of Vi-DT test typhoid conjugate vaccine in limited number of participants aged 6 months to 45 years, and same need to be established in large statistically powered phase III study. One Phase III study in Nepal will satisfy three criteria such as demonstrating immune non-inferiority of Vi-DT to locally licensed typhoid conjugate vaccine (Vi-TT or Typbar TCV®, Bharat Biotech), equivalence of immunogenicity of three lots of Vi-DT and confirming its safety in those enrolled population which is alone insufficient as per WHO-PQ required safety sample size.

This study is an additional Phase III study which has been planned in Philippines will provide safety data of Vi-DT in study population along with Nepal Phase III study and will support its WHO PQ process, which requires at least 3000 subjects' safety database in a certain population as a prerequisite for a particular product. Through this study, bridging immunogenicity data from

the multidose formulation (MD) with that of the single dose formulation (SD) of Vi-DT will also be undertaken.

## 3.4 POTENTIAL RISKS AND BENEFITS

### 3.4.1 KNOWN POTENTIAL RISKS

The Vi-DT vaccine components, Vi Polysaccharide, DT and preservative 2-PE, are licensed and in use for a long time with established safety profile. In the course of the Phase I and II trial conducted in the Philippines at RITM, Vi-DT was not associated with any severe or serious adverse event. Any vaccine could cause an anaphylactic reaction, though such reactions are rare. Expected local and systemic reactions include injection site pain, tenderness, erythema/redness, swelling/induration, pruritus associated with injection and systemic reactions such as fever, lethargy, irritability, nausea/vomiting, arthralgia, diarrhea, drowsiness, loss of appetite, chills, headache, fatigue, myalgia and persistent crying. These side effects are expected to be mild or moderate in intensity and transient and resolve spontaneously without sequelae. There may be chance of tenderness or bruising at the spot where blood will be drawn. Fainting or dizziness can occur also after a blood draw, but this is uncommon. On very rare occasions, infection can occur where the needle is inserted to draw blood.

### 3.4.2 KNOWN POTENTIAL BENEFITS

Study participants may receive no direct benefit from study participation. They will however have access to their medical records. Findings of medical concern will be referred for appropriate care and treatment. Compared with polysaccharide vaccines, conjugate vaccines are usually more immunogenic and better at inducing long term memory responses, especially in young children < 2 years of age. The potential benefits to vaccinated participants are substantial, since typhoid is endemic in several parts of the world including in the Philippines. Typhoid affects infants, young children and adults which can lead to death if not treated promptly with appropriate medical care including antibiotics. The use of an effective vaccine in all age groups will contribute to the prevention and control of typhoid and prevent death from typhoid fever.

# 4 OBJECTIVES

**Primary**

- Demonstrate the immune equivalence as measured by anti-Vi IgG GMT of multi dose formulation against single dose formulation of Vi-DT (18-45 year age stratum), 4 weeks after single dose

**Secondary**

- Demonstrate the immune equivalence as measured by seroconversion rates of anti-Vi IgG ELISA antibody titres of multi dose formulation against single dose formulation of Vi-DT vaccine (18-45 year age stratum) 4 weeks after single dose
- Describe safety profile in all age strata combined (age 6 months - 45 yrs old) and in each age stratum[#], 4 weeks after single dose of MD/SD formulation/control (Meningococcal conjugate Vaccine )

[#] Age strata of 6 months to less than 2 years, 2 years to less than 18 years, and 18 years to 45 years.

# 5 STUDY DESIGN

## 5.1 STUDY ENDPOINTS

**Primary Endpoints**

- Geometric Mean Titers (GMT) of anti-Vi IgG at 4 weeks (28 days) after vaccination of Vi-DT(MD)/ Vi-DT (SD) [18-45 year age stratum]

**Secondary Endpoints**

- Seroconversion rates (defined as a 4-fold increase of serum anti-Vi IgG antibody titer from baseline) of anti-Vi IgG ELISA antibody titers at 4 weeks (28 days) after vaccination with Vi-DT(MD) / Vi-DT (SD) compared to baseline (D0) [18-45 year age stratum]
    - Comparison of safety of V-DT compared to control: Vi-DT MD and SD will be compared separately to control vaccine and will be compared by age strata at 4 weeks after vaccination
        - Local and systemic solicited adverse events during the 7 days after vaccination *(Refer to Table 5 and 6 for details of grading of AEs)*

        - Solicited local reactions at the site of injection: pain, tenderness, erythema/redness, swelling/ induration, pruritus
        - Solicited Systemic reactions (adapted to each age group): fever, lethargy, irritability, nausea/vomiting, arthralgia, diarrhea, drowsiness, loss of appetite, chills, headache, fatigue, myalgia and persistent crying
    - Unsolicited adverse events during 4 weeks (28 days) after vaccination
    - Serious Adverse Events during the entire study period

## 5.2 METHODOLOGY

This is a multicenter, randomized, observer-blinded, controlled, immune equivalence study of a multi-dose formulation with 2PE preservative of SK bioscience Vi-DT (MD) compared to single dose formulation without preservative of SK bioscience Vi-DT (SD) in participant (6 months - 45 years) including safety population. The Vi-DT vaccines will be administered to 1,500 healthy participants of 6 months to 45 years of age and followed up for 24 weeks after the injection for safety. Adult participants (N=500) will be followed up additionally for immunogenicity 4 weeks post vaccination and all participants till 24 weeks for safety post single dose of the MD & SD formulations. 300 healthy participants will be given control vaccine (Meningococcal conjugate Vaccine) to check the background safety events. The primary objective is to demonstrate the equivalence of immunogenicity as measured by anti-Vi IgG GMT titer 4 weeks after a single dose of MD/SD formulation in adults. The secondary objective is to demonstrate the equivalence of immunogenicity in terms of seroconversion rates as measured by anti-Vi IgG ELISA antibody titers, 4 weeks after a single dose of MD/SD formulations in adults. A descriptive evaluation of safety at 4 and 24 weeks post single dose of (MD/SD/ Meningococcal conjugate Vaccine ) will be performed. The Vi-DT vaccine from both MD & SD formulations will be administered as a single dose of 25 µg.
Eligible participants enrolled into the study will be randomized into one of the three study groups within each age stratum of 6 months to less than 2 years, 2 to less than 18 years, and 18 to 45 years. Participants will be observed at the study site for 30 minutes after vaccination for immediate safety assessment. Solicited adverse events will be recorded on a diary card during 7 days after vaccination. Unsolicited adverse events will be recorded during the 4 weeks after vaccination. Serious adverse events will be recorded during the entire study period. With the exception of designated study site personnel responsible for vaccine administration, study

investigators, study nurses, and those assessing clinical outcomes, and data analysts will be blinded to vaccine allocation until database lock for the final analysis.

Blood samples will be collected at baseline prior to vaccination and at 4 weeks post vaccination from adult participants (18-45 years) only for immunogenicity assessment.

The final analysis will be performed when all participants complete the 24 weeks visit. Safety data up to 24 weeks will be included in this analysis.

### 5.2.1 JUSTIFICATION FOR DOSE OF VI-DT

Previous studies conducted with other typhoid conjugate vaccines concur to use 25 µg/0.5 mL of Vi polysaccharide. Immune response in infants aged 6-8 weeks was less than older age groups when low dose (5 µg) of conjugated typhoid vaccine was used [25] . Single dose and two doses of 25 µg/0.5 mL, and two doses of 15 µg/0.5 mL were tested in a Phase IIb study in age group 2-17 years. Single dose of 25 µg/0.5 mL of Vi-TT conjugated vaccine showed excellent immune response (100% seroconversion) and was found to be safe in infants and young children aged 6-23 months. Based on these results Bharat Biotech carried out a Phase III clinical trial with single dose 25 µg/0.5 mL [20]. The Bharat Biotech's Vi-TT vaccine is now licensed in India and several other countries and is WHO-prequalified.

Similarly, in a dose ranging study, 25 µg, 12.5 µg and 5 µg of conjugate typhoid vaccine Vi-rEPA (Vi polysaccharide conjugated to exoprotein of *Pseuomonas aeruginosa*) conducted in children 2-5 years of age in Vietnam, 25 µg was found to be safe with higher immunogenicity [26]. Further 25 µg Vi-rEPA vaccine safety and immunogenicity study was conducted in infants aged 2, 4, 6 and 12 months along routine EPI vaccines and was found to be safe and immunogenic [16]. Dose of 25 µg was found to be safe and immunogenic in participants aged 2 years and above in a Phase I (IVI T001) conducted in the Philippines and same dose is being used in Phase II clinical trial (IVI T002) in the Philippines from 6 months to 2 years of age.

**Table 1. Immunization Schedule**

| Group of Vaccinees and age group | **Age Strata** | **N*** | **D0** |
|---|---|---|---|
| **Group A**<br>750 participants<br>(6 mo - 45 yrs) | 18-45 yrs | 250 | (25 µg 0.5 mL)<br>(Vi-DT)<br>Multi dose formulation |
| | 2- <18 yrs | 250 | |
| | 6 mo-<2 yrs | 250 | |

| | | | |
|---|---|---|---|
| **Group B**<br>750 participants<br>(6 mo - 45 yrs) | 18-45 yrs | 250 | (25 µg 0.5 mL)<br>(Vi-DT)<br>Single dose formulation |
| | 2- <18 yrs | 250 | |
| | 6 mo-<2 yrs | 250 | |
| **Group C**<br>300 participants<br>(6 mo - 45 yrs) | 18-45 yrs | 100 | Control<br>(Meningococcal Conjugate vaccine) |
| | 2- <18 yrs | 100 | |
| | 6 mo-<2 yrs | 100 | |

* Age Strata wise number of enrolled subjects may be adjusted during the actual enrollment.

**Table 2. Schedule of Events**

| Visit Number | V1 | V2 | Phone Call# | V3 © | V4 © | V5 |
|---|---|---|---|---|---|---|
| Visit Day | D (-7 to 0) | D0 | D3 | D7 | D28 | D168 |
| Visit Week | -1 | 0 | | 1 | 4 | 24 |
| Visit Window | | | | ±2D | ±3D | ±7D |
| Screening | X | | | | | |
| Informed Consent/Assent | X | | | | | |
| Inclusion & Exclusion Criteria | X | X | | | | |
| Urine pregnancy test (UPT) % | X | | | | | |
| Medical History | X | X | | X | X | X |
| Vital Signs$ | X | X | | X | X | X |
| Physical Examination | X | X | | X | X | X |
| Blood Sampling @ (approx.) | | 5 mL | | | 5 mL | |
| Cumulative Blood Volume (approx.) | | 5 mL | | | 10 mL | |
| Enrollment & Randomization | | X | | | | |
| Vaccination | | X | | | | |
| Post Vaccination 30 min F/U | | X | | | | |
| Solicited AE | | X | | X | | |

| Visit Number | V1 | V2 | Phone Call# | V3 © | V4 © | V5 |
|---|---|---|---|---|---|---|
| Visit Day | D (-7 to 0) | D0 | D3 | D7 | D28 | D168 |
| Visit Week | -1 | 0 | | 1 | 4 | 24 |
| Visit Window | | | | ±2D | ±3D | ±7D |
| Unsolicited AE | | X | | X | X | |
| SAE | | X | | X | X | X |
| Diary Card Provided | | X (DC1) | | X (DC2) | X (DC3) | |
| Diary Card Collected | | | | X (DC1) | X (DC2) | X (DC3) |
| Concomitant Medications | X | X | | X | X | X* |
| Termination Record | | | | | | X |
| SAE Reporting | | To be reported at any time during the trial | | | | |

# Phone calls on Day 3 will be arranged after vaccine administration to ensure completion of the Participant's DC and to check their health status. No deviation will be considered if the phone call could not be completed on the exact date.

@ Only from Adult subjects of 18 to 45 years

***** After visit 4, only concomittant medication related to SAE will be captured

$ Blood pressure collection is for adult population of 18 years to 45 years

% Urine pregnancy test (UPT) will be performed to all female participants of childbearing age from menarche.

© During city lock down only safety follow up has been done through phone call in V3 and V4. Even the post vaccination blood sample at V4 could not be collected.

**Age Strata reassignment  (Stratawise Enrollment number of subject)**

The initial enrollment plan required an equal number of subject enrollment from each age stratum and the current plan allows for the adjustment of the required number of subjects in each stratum during the enrollment period.

Post-dose blood sample could not be collected from the enrolled adults stratum due to COVID-19. As of 13MAR2020, the Philippines government has imposed a country-wide lock down (as a result of the COVID-19 pandemic) which has impacted the ongoing activities for the clinical trial. Subjects enrollment as well as planned visit 4 blood collection (the post-vaccination blood

sample from adults stratum) has been impacted because of the lockdown.  In this situation, there will be additional subjects enrollment into the adults stratum so as to keep the initial planned statistical power for immune equivalence test as planned initilly, while reducing the enrollment of subjects in the other two age strata.  This will ensure the total number of subjects at 1800 as per original plan.

Due to city lock down those subjects whose blood sample could not be collected on V4, their post- vaccination blood sample would not be collected when lock down is lifted. This will be consider as deviation and will be informed to respective ethics committee.

## 5.3 MEASURES TO MINIMIZE BIAS

### 5.3.1 RANDOMIZATION/MASKING PROCEDURES

The randomization list will be generated by an IVI independent statistician who is not directly involved in the study conduct. Eligible participants will be assigned to receive single dose of Vi-DT test vaccine(either from multidose or singledose formulation) or control vaccine in a (2.5) : (2.5) : 1 ratio (i.e. 750, 750, and 300 participants respectively). The randomization list will contain sequential numbers unique to each participant and the block randomization process will be employed to ensure an effective balance between the interventions.

Only the independent statistician will have a complete set of randomization lists. The individual site lists will be kept under lock and key by the pharmacy staff at all clinical sites.  At the end of the study after unblinding of the participants, the lists will be returned to the statistician.

Two types of randomization list, one with randomization number only, and the second with randomization number and vaccine allocation will be prepared. Participants in the study will be randomized into three treatment groups within each age stratum: 6 to less than 24 months, 2 to less than 18 years, and 18 to 45 years. Randomization list “without the vaccine allocation” with numbers will only be shared with the blinded trial staff, for enrolling the trial participants and assigning them the randomization number. The randomization list “with the vaccine allocation” will be shared with the unblinded vaccine administrator (study nurse/pharmacist). If feasible, a web based randomization system may be provided to each site and in that case paper based randomization list will not be used.

Upon enrollment, in order to receive the study vaccine, participants will be sent to the vaccine administrator with their randomization number. The unblinded study nurse/pharmacist located in

a different room will administer vaccine(s) to the participant according to the randomization list. The randomization number of the participant receiving the study vaccine will be written on the empty vaccine vial and on the vaccine accountability log for record and reconciliation.

Trial staff other than the unblinded study staff will remain blinded to vaccine administration. The unblinded study nurse/pharmasist will not be involved in the evaluation of vaccine safety and will not discuss with the investigator and clinical staff about vaccines administered.

### 5.3.2 BLINDING

The PI, study staff, and participants will be blinded as to receipt of study vaccine or comparator. The pharmacy staff preparing the vaccine syringes and the study nurse who is administering the vaccine will not be involved in the safety assessment of participants and will be instructed not to comment on the experimental agent to study staff. For all participants, the volume of injection will be consistent at 0.5mL (25ug). The site pharmacy staffs and the study nurse must sign a confidentiality agreement not to discuss randomization codes or participant assignments.
The enrollment/randomization number with its treatment assignment is generated in advance by the independent study statistician. The randomization information is only available to the study nurse/pharmacist and independent statistician. Enrollment/Randomization numbers will be assigned sequentially by the study nurse/pharmacist upon confirmation of eligibility and enrollment into the study according to their age strata by the Investigator. All CRFs and source documents will be labeled with the randomization number and study visit number. Personal identifying information linking the study number to an individual volunteer will not be captured on CRF as study data. This linkage if site requires will be maintained electronically in a secured, password protected computer on site for the purpose of volunteer scheduling and verification of identity.

### 5.3.3 UNBLINDING OF PARTICIPANTS

A request for unblinding, with its rationale, must be forwarded through the PI. The PI will evaluate the request and will notify the Study Medical Monitor (SMM). The SMM will evaluate the request and will advise the Sponsor regarding a course of action. The Sponsor will decide whether to approve the request for unblinding. In the case of the former, the Sponsor will authorize the independent statistician to provide this information to the PI. It should be noted that there are very few circumstances in which unblinding will be essential to the medical management of a vaccine (or comparator) recipient. In case of vaccine-related death or life

threatening, serious adverse events (SAEs), knowledge of whether a participant received vaccine or comparator can be critical for the interpretation of the significance of clinical findings and thus impact decisions regarding continuation of study participation. In such cases, the assignment of a participant may be unblinded.

Episodes of unblinding, whether accidental or intentional, will be reported by the site investigator (either by email or fax) with an explanation to the sponsor at IVI who will in its turn inform IVI IRBs. Apart from this, the Site PI is also responsible for informing their own IRB. Other participating IRBs will be informed through Sponsor. Follow-up of such participants will continue throughout the duration of the trial.

Study participants will be unblinded after the completion of the final analysis. A primary analysis will be undertaken after 4 weeks of vaccine administration where both safety and immunogenicity (in adults stratum only) will be assessed.The database will be locked for the final analysis after safety and immunogenicity data is cleaned and locked at 24 weeks. Two clinical study reports are planned.

# 6 STUDY AGENT

## 6.1 STUDY AGENT(S) AND CONTROL DESCRIPTION

### 6.1.1 ACQUISITION

Test vaccine (multidose and single dose pesentations) manufactured by SK bioscience in the Republic of Korea will be shipped to central vaccine depot in the Philippines. Study sites will receive the vaccine from central vaccine depot. Locally licensed  meningococcal conjugate vaccine will be purchased as Control vaccine through local distributor and will also be distributed through central vaccine depot. The pharmacist will receive the study products and will be responsible for accounting, storage, handling and administration of the vaccines. The detail working process of central vaccine depot will be narrated in the Manual of Procedure (MOP).

### 6.1.2 FORMULATION, APPEARANCE, PACKAGING, AND LABELING

**Test Vaccine**

**(Multi-dose formulation)**

1) Code name: NBP618-MD
2) Manufacturer: SK bioscience Co., Ltd.

3) Ingredient: Purified Vi-polysaccharide conjugated to diphtheria toxoid with preservative 2 PE
4) Appearance: Clear, colorless liquid
5) Dose: 25 µg of Vi polysaccharide/0.5 mL (5 doses per each multi-dose vial), presented in Type I glass vial

**(Single dose formulation)**

1) Code name: NBP618-SD
2) Manufacturer: SK bioscience Co., Ltd.
3) Ingredient: Purified Vi-polysaccharide conjugated to diphtheria toxoid without any preservative
4) Appearance: Clear, colorless liquid
5) Dose: 25 µg of Vi polysaccharide/0.5 mL, presented in Type I glass vial

**Table 4. Test Vaccine Content**

| Function | Component | | Reference | Quantity/ 0.5 mL |
|---|---|---|---|---|
| | Multi Dose (MD) | Single Dose (SD) | | |
| Active ingredient | Vi polysaccharide conjugated to diphtheria toxoid | Vi polysaccharide conjugated to diphtheria toxoid | In-house | 25 µg |
| Stabilizer | Di Sodium hydrogen phosphate | Di Sodium hydrogen phosphate | EP | 0.620 mg |
| Stabilizer | Sodium Dihydrogen Phosphate dihydrate | Sodium Dihydrogen Phosphate dihydrate | EP | 0.152 mg |
| Stabilizer | Sodium chloride | Sodium chloride | EP | 4.25 mg |
| Diluent | Water for injection | Water for injection | EP | q.s. |
| Preservative | 2-phenoxy ethanol | | EP | 5.0 mg |

6) Packaging: Test vaccine (Vi-DT Multi dose) and 'Vi-DT Single dose' will be manufactured and packaged by SK bioscience and supplied to the sponsor designated central vaccine

depot in the Philippines. Study pharmacy nurse at the clinical trial site will receive the vaccine from central vaccine depot. Those Vaccine will be labelled by SK bioscience as either Vi-DT Multi dose (MD) or Vi-DT Single dose (SD).

7) Labeling: will be done as per health science authority guidelines section 3.3 [27] Investigational Medicinal Product Dossier, the template of vial and carton label are as follows:
8) Storage condition: hermetic container (3 mL multi-dose glass vial), protected from light, +2 to +8°C. DO NOT FREEZE
9) Retest period: 18 months from date of manufacturing

Test Vaccine vial label**:** (Multi-dose formulation)

| |
|---|
| "For Clinical Trial Use Only"<br>**Protocol No. :** IVI T004<br>**Drug Code:** NBP618-MD<br>**Lot No. :** S121902<br>**Randomization No.:**O_OOOO<br>**Retest Date :** Up to 18 months from the date of manufacture (Mfd. Date: 2019.09.25 )<br>Storage Condition: hermetic container, protected from light, +2ºC to 8ºC without freezing<br>Manufacturer: SK bioscience Co., Ltd. |

Box Label: (Multi-dose formulation)

| |
|---|
| "For Clinical Trial Use Only"<br>**Protocol No. :** IVI T004<br>**Drug Code:** NBP618-MD<br>**Lot No. :** S121902<br>**Retest Date:** Up to 18 months from the date of manufacture (Mfd. Date: 2019.09.25 )<br>**Storage Condition:** hermetic container, protected from light, +2 °**C** to +8°**C** without freezing<br>**Manufacturer and Address:** SK bioscience Co., Ltd., 150, Saneopdanji-gil, Pungsan-eup, Andong-si, Gyeongsangbuk-do, Republic of Korea<br>**Dosage and administration:** 0.5mL/dose with intramuscular injection |

Test Vaccine vial label**:** (Single-dose formulation)

| |
|---|
| “For Clinical Trial Use Only”<br>**Protocol No. :** IVI T004<br>**Drug Code:** NBP618-SD<br>Lot No. : CTSAV104<br>**Randomization No.:** O_OOOO<br>**Retest Date :** Up to 24 months from the date of manufacture (Mfd. Date: 2019.11.20 )<br>**Storage Condition:** hermetic container, protected from light, +2°**C** to 8°**C** without freezing<br>Manufacturer SK bioscience Co., Ltd. |

Box Label: (Single-dose formulation)

| |
|---|
| “For Clinical Trial Use Only”<br>**Protocol No.** : IVI T004<br>**Drug Code:** NBP618-SD<br>**Lot No. :** CTSAV104<br>**Retest Date:** Up to 24 months from the date of manufacture (Mfd. Date: 2019.11.20 )<br>**Storage Condition:** hermetic container, protected from light, +2 °**C** to +8°**C** without freezing<br>**Manufacturer and Address:** SK bioscience Co., Ltd., 150, Saneopdanji-gil, Pungsan-eup, Andong-si, Gyeongsangbuk-do, Republic of Korea<br>**Dosage and administration:** 0.5mL/dose with intramuscular injection |

**Control vaccine**

**Meningococcal conjugate vaccine**

1) Ingredient: Tetravalent meningococcal polysaccharide conjugated vaccine consisting of Neisseria meningitidis capsular polysaccharides A, C, W-135 and Y each coupled to a carrier protein
2) Appearance: The diluent (sodium chloride and water for injections) is a sterile clear and colourless liquid supplied separately in a prefilled syringe or ampoule. After reconstitution, vaccine appears as a clear and colourless solution.
3) Dose: 0.5mL, intramuscular injection
4) Packaging: Supplied as a sterile lyophilized white powder or cake in a single dose vial.
5) Labeling: Keep original label as registered in the Philippines

6) Storage condition: protected from light, stored at +2°C to 8°C

#### 6.1.3 PREPARATION

The preparation and administration of the vaccines to participants enrolled into the study will only be done by the unblinded study nurse according to the procedures stipulated in this study protocol. The unblinded study nurse responsible for vaccine administration is qualified to perform this task and same will be documented by site investigator.

The licensed vaccines will be prepared before use according to the package insert. For further details please refer to the MOP.

#### 6.1.4 DOSING AND ROUTE OF ADMINISTRATION

Vi-DT manufactured by SK bioscience, Republic of Korea, is formulated (in both Multidose and Single dose vial) to contain purified Vi-polysaccharide 25 µg in 0.5 mL per dose. The vaccine will be administered by intramuscular injection in the left anterolateral thigh or left arm deltoid region in participants below 2 years of age, less dominant arm deltoid region in age group 2-45 years. Control vaccine will also be administered at a dose of 0.5 mL through intramuscular injection.

In case the left thigh cannot be used for vaccination due to infection, eczema or injury, the right thigh will be used for vaccination instead. If the left deltoid cannot be used for vaccine administration, the right deltoid will be used instead.

#### 6.1.5 DOSE ADJUSTMENTS/MODIFICATIONS/DELAYS

There is no plan of Vi-DT dose adjustment and modification during the course of study.

#### 6.1.6 TRACKING OF DOSE

All test vaccines , control vaccine (Meningococcal conjugate vaccine) and other EPI vaccines administered will be reported in the vaccine administration log by the Study Pharmacist/Nurse. The unblinded study monitor will ensure timeline adherence for administration of study product.

### 6.2 STUDY AGENT ACCOUNTABILITY PROCEDURES

Used and unused Vi-DT will be disposed at site or returned as per the guidance from the manufacturer. All Used and unused control vaccines will be disposed at the site following

reconciliation as per the site SOP. All expired vaccines will be disposed as per the site SOP (See details in the MOP).

### 6.3 STANDARD OF CARE

- Appropriate medical care and treatment to participants in need during the trial will be provided as per standard of care practice in the Philippines.
- The site investigator will conduct appropriate medical investigations deemed necessary to evaluate any medical conditions that might arise during the course of the study and ensure that participants receive appropriate care and are referred to appropriate health services as needed.

### 6.4 CONCOMITANT MEDICATIONS, TREATMENTS, AND PROCEDURES

A prescription medication is defined as a medication that can be prescribed only by an authorized/licensed physician. Medications to be reported in the Case Report form (CRF) are concomitant prescription medications, over-the-counter medications and supplements. Because all the subjects are blinded and they don't know what vaccine they are receiving, both test groups and comparator group would be restricted from receiving additional Meningococcal Vaccination as prohibited medication once they are participating in this study until the blind code is open (For test group, to keep blinding; For comparator group, to prohibit any possibility of overdose).

## 7 STUDY POPULATION

### 7.1 STRATEGIES FOR RECRUITMENT AND RETENTION

The targeted sample size is 1800, 750 each in the test arm and the rest 300 in the control arm. Study staffs will approach participants/parent(s)/LAR between Days -7 to 0 at the health facility. The enrollment venue is the clinical trial sites in the Philippines. Participants/Parent(s)/LAR with aged 6 months to 45 years visiting health centers for regular immunizations or medical check-up who may be interested in participating in the study, will be asked to go to clinical trial site during the recruitment period. The site will ensure that any advertisements used to recruit subjects (letters, pamphlets, posters, etc.) are submitted to IVI for review and approved through site IRBs

before use. Before performing any study related activity consent or assent will be collected. After vaccination, participants will be kept under observation at the study site for 30 minutes to check for any immediate adverse events. The participant/parent(s)/LAR will be given a diary card with instructions on how to fill it out.

## 7.2 CONSENT AND ASSENT PROCEDURES AND DOCUMENTATION

Informed consent is a continuing process that is initiated prior to the individual's agreeing to participate in the study and continues throughout the individual's study participation. Since as per the Philippines National ethical Guideline (For Health and Health related Research 2017) for 7 to <12 years participants verbal assent is required, The documentation of this verbal assent will be completed in source document in the form of a written description of the process and witnessed. Apart from this, parental/LAR consent form will also be completed on this 7 to <12 year old participants Assent is a process that will be initiated for individual's aged 12 to <18 years, agreeing to participate in the study and continues throughout the individual's study participation. Apart from this parental/LAR consent form will also be completed for this 12 to <18 years participants.. Participants in the age group 18 to 45 years will be asked for consent through ICF only. However in case of participants in age group 6 months to <7 years, only consent will be asked from their Parent(s)/LAR.

| **Subject's Age (years)** | **Under 7 Years** | **7 to <12 Years** | **12 to <18 Years** | **18 Years and above** |
|---|---|---|---|---|
| **Type of Consents** | ICF [Parent(s)/LAR] | ICF [Parent(s)/LAR] | ICF [Parent(s)/LAR] | ICF (Participant) |
| | - | Verbal assent (Participant) | IAF (Participant) | - |

Information about the risks and possible benefits of participation will be provided to the participants/parent(s)/LAR through extensive discussion. Consent/Assent forms will be EC/IRB-approved prior to their use and the participants/parent(s)/LAR will be asked to read the document for their better understanding about the study.

The investigator or designated study team member will explain the study to the participants/parent(s)/LAR and answer any questions that may arise. All participants will receive

a verbal explanation in terms suited to their comprehension of the purposes, procedures, and potential risks of the study and of their rights as research participants. The participants/parent(s)/LAR will have the opportunity to carefully go through the written consent/assent form and ask questions prior to signing. Participant/parent(s)/LAR may wish to discuss the study with family or friends before making any decision as to whether or not to participate in the study and come back later to inform the site Investigator or designee of his/her decision. For those individuals who express interest in continuing with the consent/assent process, the site investigator or designee will review the consent/assent form privately in detail with the participant/parent(s)/LAR and answer any questions.

Before signing the consent/assent, participants/parent(s)/LAR will be asked to undergo an informed consent process validation to ensure that they fully understand the purpose of the study, procedures, potential risks and their rights in this study.

If the participant's representative is illiterate (i.e. not able to read and sign the ICF), then it must be signed and dated by an impartial witness who is independent of the Investigator. A witness who signs and dates the consent form is certifying that the information in this form and any other written information had been accurately explained to and understood by the subject's parent(s)/LAR. If minor participants visit the facility with LAR then valid identity document will be asked from them showing their legal guardianship.

The participants/parent(s)/LAR will sign the informed consent/assent documents prior to any procedures being done specifically for the study. The participants/parent(s)/LAR may withdraw consent/assent at any time throughout the course of the trial. A copy of the informed consent/assent document will be handed over to the participants/parent(s)/LAR for their records. The rights, safety, and welbeing of the participants will be protected by emphasizing that the quality of their medical care will not be adversely affected even if they decline to participate in this study.

* For both Parental consent and Participant consent, the same consent form will be used.

## 7.3 COMPENSATION FOR PARTICIPATION

Participants will not be compensated for participation in the study. However a prorated and reasonable reimbursement will be given to the participants/parent(s)/LAR for travel expenses and time lost from work / time spent at the study site will be given as approved by IRBs.

## 7.4 PARTICIPANT INCLUSION CRITERIA

In order to be eligible to participate in this study, any individual must meet the following criteria:

1. Healthy participants 6 months to 45 years of age at enrollment
2. Participants/Parent(s)/LAR who have voluntarily given informed consent/assent
3. Participants/Parent(s)/LAR willing to follow the study procedures of the study and available for the entire duration of the study

## 7.5 PARTICIPANT EXCLUSION CRITERIA

An individual who meets any of the following criteria will be excluded from participation in this study:

1. Child with a congenital abnormality
2. Participant who has already received meningococcal conjugate vaccine.
3. Participant concomitantly enrolled or scheduled to be enrolled in another trial
4. Known history of immune function disorders including immunodeficiency diseases (Known HIV infection or other immune function disorders)
5. Chronic use of systemic steroids (>2 mg/kg/day or >20 mg/day prednisone equivalent for periods exceeding 10 days), cytotoxic or other immunosuppressive drugs
6. Receipt of blood or blood-derived products in the past 3 months
7. Participant with a previously ascertained or suspected disease caused by *S. Typhi* (confirmed either clinically, serologically or microbiologically)
8. Participant who has had household contact with and/or intimate exposure to an individual with laboratory-confirmed *S. Typhi*
9. Individual who has previously received a typhoid vaccine
10. Participant who has received other vaccines from 1 month prior to test vaccination or planned to receive any vaccine within 1 month (except a measles containing vaccine as per government vaccination campaign)
11. Known history or allergy to vaccines or other medications
12. History of uncontrolled coagulopathy or blood disorders
13. Any abnormality or chronic disease which in the opinion of the investigator might be detrimental for the safety of the participants and interfere with the assessment of the study objectives
14. Any female participant who is lactating, pregnant* or planning for pregnancy during the course of study period

15. Participant/Parent(s)/LAR planning to move from the study area before the end of study period
16. As per Investigator's medical judgement individuals could be excluded from the study in spite of meeting all inclusion/exclusion criteria mentioned above

**Temporary Contraindication**

1. Acute illness, in particular infectious disease or fever (axillary temperature ≥37.5°C), within three days prior to enrollment and vaccination
    a. This individual could be rescreened upon resolution of the above said conditions

*Urine pregnancy test (UPT) will be performed to all female participants of childbearing age from menarche.

## 7.6 STUDY PROCEDURES

### 7.6.1 SCREENING

**Informed Consent / Assent Process**

The informed consent/assent process will be conducted as described above. The date of signature of the informed consent/assent will be entered on the eCRF.

The screening process will take place during the week prior to enrollment (Days -7 to 0). After written consent/assent are obtained, screening procedures will be performed as described in the MOP. The study staff will request the screened eligible participants/parent(s)/LAR to visit trial site for vaccination which will be considered as Day 0. A participant will be enrolled if he/she meets all inclusion and none of the exclusion criteria and his/her health status is deemed acceptable as determined by medical history, physical examination, and medical judgement of the site investigator. If participant/parent(s)/LAR agrees, screening and enrollment could be done on the same day. A study ID card will be provided to each enrolled participant.

Details of visit procedures during screening are as follows:

**Visit 1 - Screening visit (Days -7 to 0)**

1) Explain study objectives and procedures, risk and benefits to the participant /parent(s) /LAR
2) Perform informed consent process validation

3) Obtain written informed consent/assent from the participant/parent(s)/LAR. Written Informed consent/assent/verbal assent will be obtained prior to performance of any study-specific screening or evaluations
4) Perform screening procedures within 0 to 7 days prior to study vaccination
5) Collect demographics, medical history, vital signs and check for inclusion and exclusion criteria
6) All females volunteers of child bearing age will be screened for pregnancy with UPT and whether using any contraceptive method or not
7) Schedule participants for enrollment and vaccination visit (V2) at the study center within 7 days after the screening visit. If an eligible participant does not come back for V2 within the 7 days after the screening visit, screening procedures must be repeated
8) Screening and enrollment could be done on the same day. In this case screening related information can be captured in Visit 2 page of eCRF
9) Participant who does not meet the criteria for participation in this trial because of fever or acute illness, may be rescreened when these conditions have resolved. Rescreened participant will be assigned the same screening number as for the initial screening

### 7.6.2 ENROLLMENT

Informed consent form will be verified by study staff and inclusion and exclusion criteria will be reviewed.

Participants will be randomized to test/control groups into three age strata of age 6 months to less than 2 years, 2 to less than 18 years, and 18 to 45 years. Participants will be attributed an enrollment number. Before randomization, medication history will be obtained, vital signs and results from physical examination, growth and development evaluations will be recorded. 5 mL of blood for the immunogenicity will be obtained prior to IP administration.

Details of visit procedures during enrollment are described below and in the MOP:

**Visit 2 - Enrollment and Study Vaccination (Day 0)**

1) Collect medical history, perform clinical examination and check concomitant therapies and record in the eCRF
2) Confirm eligibility of participant (include urine pregnancy test for female participants attend menarche)
3) Perform enrollment, randomization and attribute enrollment number to participant

4) Perform vaccination as per instructions of the MOP
5) Monitor participant for 30 min following vaccination as follows:
    - Local examination of injection site
    - Clinical examination including vital signs, general physical examination before leaving the study center 30 (±5) min post vaccination
    - Record solicited and unsolicited adverse reactions (if any occurrence is there)
6) Schedule next visit to the study center and remind participant(s)/parent/LAR to bring the diary card at the next visit
7) Participant/parent(s)/LAR will be instructed to evaluate local and systemic reactions at home for 7 days post immunization (from Day 0 to Day 6 post vaccination day). Diary card 1 will be issued to record adverse events and participant/parent(s)/LAR will be instructed how to fill in the diary card 1. A thermometer and ruler will be given along with diary card to record fever and measure local reactions (e.g. diameter of redness, swelling, etc). Next clinic visit after 7 days will be scheduled. Participant/parent(s)/LAR will also be instructed to contact investigator/ study staff if needed.

### 7.6.3 FOLLOW-UP PROCEDURES AND VISITS

**Days 0-6**

The study staff will contact the participant/parent(s)/LAR by telephone three days after vaccination (Day 3 Phone call) to ensure the completeness of Diary Card and to check the health status of their participants and remind the date of the next visit day.

**Visit 3 – Safety follow-up visit (Day 7 ± 2 day)**

1) Record solicited and unsolicited adverse events since the last visit in eCRF
2) Verify that the participant/parent(s)/LAR fill in the diary card 1correctly
3) Perform clinical examination (including vital signs and general physical exam) and record in eCRF
4) Collect interval medical history in order to capture any AEs and SAEs through interview
5) Hand over the diary card 2 to be filled till day 28 post vaccination
6) Schedule next visit, i.e. V4 and remind the participant parent(s) or legal guardian to contact investigator/ study staff if needed

**Visit 4 – Follow-up visit (Week 4, Day 28 ± 3 days)**

1) Check diary card 2 and confirm with participant/parent(s)/LAR before recording observations in the eCRF
2) Perform clinical examination including vital signs, general physical examination
3) Collect interval medical history in order to capture any AEs and SAEs through interview. Record any unsolicited AE, SAE, and concomitant medications in eCRF
4) Perform blood draw (from Adult stratum) for assessment of immune responses post vaccination
5) Schedule next visit i.e. V5 and hand over diary card 3 to participant/parent(s)/LAR to capture any SAEs till 24 weeks (168 days) post vaccination

**Visit 5 - Follow-up visit (Week 24, Day 168 ± 7 days)**

This is the final visit of the study.

1) Collect diary card 3 and check with participant/parent(s)/LAR before recording observations in the eCRF
2) Perform clinical examination including vital signs, general physical examination
3) Collect interval medical history in order to capture any SAEs through interview. Record any SAE and concomitant medications in eCRF
4) Ensure source documents are duly completed before the participant/parent(s)/LAR leave the study center
5) Participant will be continued to be monitored after study ends for unresolved AE

While in the study the female participant of child bearing age should not get pregnant. As such they should discuss with their study doctor if their current contraception can give them a reliable protection against getting pregnant. If any participant becomes pregnant during the conduct of the study, then the participant would be followed up until delivery and informations will be captured in pregnancy form. Detailed information regarding pregnancy such as method of delivery, duration of pregnancy and outcome to both mother and baby will be captured and mentioned in safety management plan.

## 7.7 PARTICIPANT WITHDRAWAL OR TERMINATION

### 7.7.1 REASONS FOR WITHDRAWAL OR TERMINATION

Participant/parent(s)/LAR are free to withdraw from participation in the study at any time upon their request, without justification and without any effect to the standard healthcare due to them.

The Site Investigator may also decide to discontinue participation of a participant from study interventions in the following cases:

1) An acute reaction (allergy, hypersensitivity reaction, etc.) to the investigational product
2) Occurrence of an illness or serious adverse event or adverse event that in the judgment of the investigator may be detrimental for the participant's safety
3) A study participant/parent(s)/LAR withdrawal of informed consent
4) A study participant's medical condition or use of medication that in the judgment of the investigator may compromise the participant's safety and/or the scientific integrity of the study
5) Violation of the inclusion/exclusion criteria by the participant
6) A study participant's no-show for a scheduled visit without notice, unable to contact/trace, and lost to follow-up
7) Any other reason of study discontinuation as per the judgment of the site Investigator

### 7.7.2 HANDLING OF PARTICIPANT DISCONTINUATION OR TERMINATION

Discontinuation from study intervention does not mean discontinuation from the study, and remaining study procedures will be completed as indicated in the study protocol. If a clinically significant finding is identified after enrollment, the PI will determine if any change in participant management is needed. Any new clinically relevant finding will be reported as an adverse event.

Study team will encourage withdrawn or terminated participant to continue in the study for safety follow-up. If participant/parent(s)/LAR decline, this will end the participant's/parent(s)'/LAR's interaction with the study team for this protocol. The study team will engage in no further communication with the volunteer except as directed by an IRB with regard to participant safety information. Protocol-specified safety follow-up procedures will be discussed with the participant/parent(s)/LAR to capture AEs, SAEs. The reason for participant discontinuation or withdrawal from the study will be recorded on the study follow-up eCRF. Only data and samples already collected will be analyzed according to protocol. Counseling about any issue will be provided if participant/parent(s)/LAR decide to discontinue participation in the study. Medical advice will also be provided in the best interest of the participant.

Participants who receive the study intervention and subsequently withdraw, or are withdrawn or discontinued from the study will not be replaced.

In the event of early termination of a participant:

1) Date and Reason for early termination of the participant will be recorded in the eCRF
2) Any unsolicited AE, SAE and concomitant medications up to that points will be recorded in eCRF
3) Clinical examination including vital signs, and general physical examination will be performed if participant/parent(s)/LAR is/are willing

## 7.8 LOST TO FOLLOW-UP

A participant will be considered lost to follow-up if he or she fails to return for any of scheduled visits and remains unreachable to study site staff.

The following actions will be taken if a participant fails to return to the site for a required study visit:

- The site staff will attempt to contact the participant/parent(s)/LAR and counsel on the importance of maintaining the assigned visit schedule and ascertain if the participant/parent(s)/LAR wishe to and/or should continue in the study
- Before a participant is deemed lost to follow-up, the investigator or designee will make every effort to regain contact with the participant/parent(s)/LAR (will make at least 3 telephone calls on separate days). These contact attempts should be documented in the participant's medical record or study file

Should the participant continue to be unreachable, he/she will be considered to have withdrawn from the study with a primary reason of lost to follow-up.

## 7.9 PROTOCOL DEVIATIONS

A protocol deviation is any non-compliance with the clinical trial protocol or GCP by the participant, the investigator, or the study site staff. It is the responsibility of the site investigator to use continuous vigilance to identify and report all major protocol deviations to site IRB and sponsor. Minor protocol deviations could be reported to IRBs along with periodic study report submission as per local IRBs requirements. The site investigator is responsible for knowing and

adhering to the site IRB requirements. The IVI Study Medical Monitor will report all protocol deviations to IVI IRB.

Major deviations are defined as those jeopardize the safety or rights of the participant or the scientific integrity of the study which may be applicable to cases listed below.

- Violation of inclusion and exclusion criteria
- Vaccination with wrong vaccine as defined in the protocol
- Visit outside window for the immunogenicity assessment after discussion with the study medical monitor
- Missed samples for immunogenicity

Major protocol deviations thought to affect the scientific integrity of the study will be reported and discussed with investigator, monitor, sponsor, and statistician for their exclusion from the per protocol analysis. For minor protocol deviations considered not to affect the scientific integrity of the study, the extent of deviation or delay as well as reason will be accurately documented.

## 7.10 PROTOCOL AMENDMENTS

Any amendment of the approved protocol shall be submitted to all IRBs (IVI and site IRBs) and to the National Regulatory Authority (PFDA) for information and approval before implementation.

## 7.11 PREMATURE TERMINATION OR SUSPENSION OF STUDY

This study may be temporarily suspended or prematurely terminated if there is sufficient reasonable cause as per PI recommendation after consultation with sponsor. PFDA, site IRBs independent Data Safety Monitoring Board (i.e., DSMB) and Safety Monitoring Committee (i.e., SMC) may also instruct for suspension or termination of the study. Written notification, documenting the reason for study suspension or termination, will be provided by the suspending or terminating party to investigator, the sponsor, the regulatory authorities, and IRBs.

Circumstances that may warrant termination or suspension are:

- Determination of unexpected, significant, or unacceptable risk to participant as recommended by the PI

- Poor protocol compliance

Study may resume once concerns about safety, protocol compliance, data quality are addressed and satisfy the sponsor, PFDA, Site IRBs or DSMB/SMC.

## 7.12 END OF STUDY

A participant is considered to have completed the study if he or she has completed all procedures of the study including the last visit or the last scheduled procedure shown in the Schedule of Events (SOE).

# 8 LABORATORY PROCEDURES/EVALUATIONS

## 8.1 SPECIMEN PROCESSING, HANDLING, AND STORAGE

Venous blood will be collected from participants for immunogenicity assessments from adult stratum only. Whole blood will be centrifuged, and sera will be aliquoted and stored at below -20°C until shipment to IVI and storage for future use. Pre-print study labels provided in advance will be attached on each of the serum aliquots.

## 8.2 SPECIMEN SHIPMENT

Aliquoted blood samples for immunogenicity assessment will be shipped from clinical trial site to the International Vaccine Institute, Seoul, Republic of Korea, where they will be stored at -70°C for analysis and storage for 10 years after completion of CSR or according to subject's decision about preservation period.

## 8.3 ASSESSMENT OF IMMUNOGENICITY

*Anti-Vi IgG ELISA*

The assay is used to measure anti-Vi specific antibodies of the IgG in human sera. Poly-L-lysine is pre-coated and purified Vi antigen at a concentration of 2 µg/mL is absorbed onto 96-well

microtiter plates. Non-specific binding sites are blocked with Bovine Serum Albumin (BSA) in Phosphate Buffered Saline (PBS), diluted human sera is then added to the first wells in the plate then serially diluted across the plate. Antibodies specific to Vi will bind to the Vi coated to the plate. The bound IgG is detected using alkaline phosphatase labelled goat anti-human IgG. Addition of 4-nitrophenyl phosphate substrate causes a color change proportional to the amount of human anti-Vi IgG antibody present in the serum. Optical Densities of the wells are measured at 405 nm. The level of the specific anti-Vi IgG in ELISA units for each serum sample is determined by comparison to a reference serum.

# 9 ASSESSMENT OF SAFETY

## 9.1 SAFETY ASSESSMENT

The following procedures will be performed to monitor safety as listed in the SOE:

- ***Demographic and medical history*** *(DOB, age, gender, baseline medical history of participants)*
- ***Physical examination*** *(height/length and weight, organ systems, growth and development and motor assessments for age eligible participants)*
- ***Vital signs*** *[temperature, pulse, respirations, blood pressure (only for 18-45 years)]*
- **Diary cards** will be used for participant/parent(s)/LAR reported outcomes

At each study visit, the investigator will inquire about the occurrence of AE/SAEs since the last visit.

### 9.1.1 DEFINITION OF ADVERSE EVENTS (AE)

**Adverse events (AE):** These are defined as any untoward medical occurrence which follows immunization and which does not necessarily have a causal relationship with the administration of the vaccine. An AE may be any unfavorable or unintended sign, symptom, abnormal laboratory finding or disease.

**Adverse Drug Reactions (ADR):** All noxious and unintended responses to a medicinal product related to any dose should be considered adverse reactions (AR). The phrase “responses to a medicinal product” means that a causal relationship between a medicinal product and an AE is exist with at least a reasonable possibility.

**Solicited AEs** are predetermined events, identified in the Investigator's Brochure (IB), which may reflect safety concerns related to the investigational product. AEs that will be solicited by the participant/ parents/LAR and recorded in the diary card and reviewed by a blinded observer during the 7 days after each dose for this study include:

- Local reactions at the site of injection: pain/tenderness, erythema/redness, induration/swelling, pruritus  associated with injection
- Systemic reactions (adapted by age group): fever, lethargy, irritability, nausea/vomiting, arthralgia, diarrhea, drowsiness, loss of appetite, chills, headache, fatigue, myalgia and persistent crying

Among the various systemic reactions headache, fatigue, myalgia will be captured only for 2 to 45 years  subjects while persistent crying will be captured only for 6 months to 2 years subjects . The rest of the systemic reactions will be captured in all age strata. This is also clearly mentioned in the diary cards.

**Unsolicited AEs** are all other adverse events (those that do not fall under the categories of solicited Adverse Reactions) that are identified by site staff, the site investigator and the Safety Medical Monitors. These unsolicited AEs will be documented in the participant's study records and entered in the study eCRFs.

Results will be expressed as frequency of the AEs and individual descriptions will be tabulated according to MedDRA organ class system.

### 9.1.2 DEFINITION OF SERIOUS ADVERSE EVENTS (SAE)

An AE or suspected adverse reaction is considered "serious" if, in the view of either the investigator or sponsor, it results in any of the following outcomes:

- Results in death
- Life-threatening event
- Requires in-patient hospitalization > 24 hours or prolongation of existing hospitalization
- Results in persistent or significant incapacity or substantial disruption of the ability to conduct normal life functions
- Congenital anomaly/birth defect
- Important medical events that may not result in death, be life-threatening, or require hospitalization may be considered serious when, based upon appropriate medical judgment, they may jeopardize the participant and may require medical or surgical intervention to prevent one of the outcomes listed in this definition.

#### 9.1.3 DEFINITION OF SUSPECTED UNEXPECTED SERIOUS ADVERSE EVENT (SUSAR)

**Suspected unexpected serious adverse event (SUSAR)** is defined as a serious adverse reaction whose nature or severity is not consistent with the applicable product information, VI-DT Investigator's Brochure or the summary of product characteristics of an authorized product.

### 9.2 CLASSIFICATION OF AN ADVERSE EVENT

#### 9.2.1 SEVERITY OF EVENT

All solicited AEs in the study will be graded for their severity and recorded in the eCRF as described in below table. All other AEs will be assessed by the study clinician using the National Institute of Allergy and Infectious Diseases (NIAID) Division of AIDS Table for grading the severity of Adult and Pediatric Adverse events [28].

**Table 5. Solicited systemic adverse reaction severity grading**

| Systemic (General) | Mild (Grade1) | Moderate (Grade2) | Severe (Grade3) | Potentially Life Threatening (Grade 4) |
|---|---|---|---|---|
| Fever* | 38.0 – 38.5°C | 38.6 – 39.2°C | 39.3 – 39.9°C | ≥ 40°C |
| Lethargy | No interference with routine activity | Some interference with routine activity | Significant; prevents daily routine activity | ER visit or hospitalization |
| Irritability | Require minimal or no treatment | Results in low level of inconvenience or concern | Interrupt daily activity and require drug therapy | ER visit or hospitalization |
| Nausea/ Vomiting | No interference with routine activity | Some interference with routine activity | Significant; prevents routine daily activity | ER visit or hospitalization |

| | | | | |
|---|---|---|---|---|
| Arthralgia | No interference with routine activity | Some interference with routine activity | Significant; prevents daily routine activity | ER visit or hospitalization |
| Diarrhea | No Interference with routine activity 1-2 episodes/24 hours | Some Interference with routine activity > 2 episodes/24 hours | Prevents daily activity requires outpatient IV hydration | ER visit or hospitalization for hypotensive shock |
| Drowsiness | No interference with routine activity | Some interference with routine activity | Significant; prevents daily routine activity | ER visit or hospitalization |
| Loss of appetite | Require minimal or no treatment | Results in low level of inconvenience or concern | Require drug therapy | ER visit or hospitalization |
| Chills | No interference with routine activity | Some interference with routine activity | Significant; prevents daily routine activity | ER visit or hospitalization |
| Headache | No interference with routine activity | Some interference with routine activity | Significant; prevents daily routine activity | ER visit or hospitalization |
| Fatigue | No interference with routine activity | Some interference with routine activity | Significant; prevents daily routine activity | ER visit or hospitalization |
| Myalgia | No interference with routine activity | Some interference with routine activity | Significant; prevents daily routine activity | ER visit or hospitalization |
| Persistent crying | Require minimal or no treatment | Results in low level of inconvenience or concern | Interrupt daily activity and require drug therapy | ER visit or hospitalization |

* Axillary temperature will be recorded.

**Table 6. Solicited local adverse reaction severity grading**

| Local Reaction to Injectable Product | Mild (Grade 1) | Moderate (Grade 2) | Severe (Grade 3) | Potentially Life Threatening |
|---|---|---|---|---|

| | | | | (Grade 4) |
|---|---|---|---|---|
| Pain /Tenderness | Does not interfere with routine activity | Interferes with routine activity or repeated use of non-narcotic pain reliever | Prevents routine daily activity or repeated use of narcotic pain reliever | Emergency room (ER) visit or hospitalization |
| Erythema/Redness (Adolescents & adults, age ≥12yrs) | Affected area 25 - < 50mm in diameter | Affected area 50-<100mm in diameter | Affected area ≥ 100 mm in diameter | Necrosis or exfoliative dermatitis |
| Erythema/Redness (Children, age 2<12 yrs) | < 25 mm in diameter | 25 – 50 mm in diameter | ≥ 50 mm in diameter | Necrosis or exfoliative dermatitis |
| Erythema/Redness (Children, age < 2 yrs) | < 10 mm in diameter | 10 < 25 mm in diameter | 25 < 50 mm in diameter | ≥ 50 mm in diameter |
| Swelling/Induration (Adolescents & adults, age ≥12yrs) | Affected area 25 - < 50mm in diameter | Affected area 50-<100mm in diameter | Affected area ≥ 100 mm in diameter | Necrosis or exfoliative dermatitis |
| Swelling/Induration (Children, age 2<12 yrs) | < 25 mm in diameter | 25 – 50 mm in diameter | ≥ 50 mm in diameter | Necrosis or exfoliative dermatitis |
| Swelling/Induration (Children, age < 2 yrs) | < 10 mm in diameter | 10 < 25 mm in diameter | 25 < 50 mm in diameter | ≥ 50 mm in diameter |

| | | | | |
|---|---|---|---|---|
| Pruritis associated with injection | Itching localized to injection site that is Relieved spontaneously or with < 48 hours treatment | Itching beyond the injection site that is not generalized OR Itching localized to injection site requiring ≥ 48 hours treatment | Generalized itching causing inability to perform usual social & functional activities | NA |

All unsolicited adverse events observed by investigator and/or reported by participants/parent(s)/LAR after discussing with the investigator will be recorded in the eCRFs with their severity grading and relatedness to the study vaccine.

All SAEs irrespective of their causal association will also be graded for their severity.

### 9.2.2 RELATIONSHIP TO INVESTIGATIONAL PRODUCT

For all collected AEs, the investigator who examines and evaluates the participant will determine the relationship of each AE with the investigational product based on plausible biologic mechanism, temporal relationship of occurrence after administration of the investigational product, identification of possible alternative etiologies including underlying disease, concurrent illness or concomitant medication, and his/her clinical judgment. The relationship of vaccination to adverse event (AE) will be determined based on the definitions below.

- **Definitely Related** – There is clear evidence to suggest a causal relationship, and other possible contributing factors can be ruled out. The clinical event, including an abnormal laboratory test result, occurs in a plausible time relationship to vaccine administration and cannot be explained by concurrent disease or other drugs or chemicals. The response to withdrawal of the Vaccine (dechallenge) should be clinically plausible.
- **Probably Related** – There is evidence to suggest a causal relationship, and the influence of other factors is unlikely. The clinical event, including an abnormal laboratory test result, occurs within a reasonable time after administration of vaccine, is unlikely to be attributed to concurrent disease or other drugs or chemicals, and follows a clinically reasonable response on withdrawal (dechallenge).

- **Possibly Related –** There is some evidence to suggest a causal relationship (e.g., the event occurred within a reasonable time after administration of vaccine). However, other factors may have contributed to the event (e.g., the participant clinical condition, other concomitant events). Although an AE may rate only as "possibly related" soon after discovery, it can be flagged as requiring more information and later be upgraded to "probably related" or "definitely related," as appropriate.
- **Unlikely to be related –** A clinical event, including an abnormal laboratory test result, whose temporal relationship to vaccine administration makes a causal relationship improbable (e.g., the event did not occur within a reasonable time after administration of vaccine) and in which other drugs or chemicals or underlying disease provides plausible explanations (e.g., the participant clinical condition, other concomitant treatments).
- **Not Related –** The AE is completely independent of vaccine administration, and/or evidence exists that the event is definitely related to another etiology. There must be an alternative, definitive etiology documented by the clinician.

The criteria for determining causality can be documented as follows: The initial four definitions can be considered as ADR and the last one i.e. "Not Related" can be considered as Non-ADR.

### 9.2.3 EXPECTEDNESS

The Study Medical Monitor in consultation with site PI will be responsible for determining whether an AE is expected or unexpected. An Adverse Reaction will be considered unexpected if the nature, severity, or frequency of the event is not consistent with the risk information previously described for the study agent.

## 9.3 TIME PERIOD AND FREQUENCY FOR EVENT ASSESSMENT AND FOLLOW-UP

All participants will be observed for immediate local and systemic reactions for 30 minutes after each vaccination. For 7 consecutive days (Days 0-6) after each dose of study vaccine, the participant/parent(s)/LAR will be asked to record solicited local and systemic symptoms in the diary card. The study staff will remind participant/parent(s)/LAR of the importance of properly filling the diary cards and to return the cards at the next scheduled study visit. If they did not fill up or lost their card, the parent(s) or legal guardian will be interviewed for recall of symptoms with trained study staff during clinic visit on Day 7 after vaccination.

The occurrence of an adverse event (AE) will come to the attention of study personnel during study visits and interviews of a study participant presenting for medical care, or upon review by a study monitor.

All AEs including local and systemic reactions not meeting the criteria for SAEs will be captured on the appropriate case report form (eCRF). Information to be collected includes event description, time of onset, symptoms and physical examination findings, clinician's assessment of severity, relationship to study product (as assessed by the SI), medications given and time of resolution/stabilization of the event. All AEs occurring while on study will be documented appropriately regardless of relationship.

Changes in the severity of an AE will be documented to allow an assessment of the duration of the event at each level of severity to be performed. AEs characterized as intermittent require documentation of onset and duration of each episode.

The investigator will record all reportable events with start dates occurring any time after informed consent is obtained until the last day of study participation. At each study visit, the investigator will inquire about the occurrence of AE/SAEs since the last visit. Events will be followed for outcome information until resolution or stabilization.

## 9.4 REPORTING PROCEDURES

### 9.4.1 ADVERSE EVENT RECORDING AND REPORTING

Adverse events, solicited AEs, and SAEs will be assessed at various study visits, documented in the source record, and recorded in the eCRF using accepted medical terms and/or the diagnosis that accurately characterize the event. When the diagnosis is known the AE term recorded in the eCRF will be the diagnosis rather than constellation of symptoms. The SI will assess all AEs for seriousness, relationship to investigational product, severity, and other possible causes.

The timeframe for the collection of adverse events (AEs) occurring from the first administration of investigational product through Visit 4 for all study groups and will be collected as well as recorded in the source document and eCRF. However SAEs will be collected and recorded throughout the study period i.e. starting from the first administration of investigational product through to the end of the trial.

When an AE has not resolved by the current visit it will be documented in the eCRF as ongoing. Documentation will include date of onset, detailed description of the event and relevant history and physical examination, severity, attribution of the AE, treatment given and date the AE improved or resolved. The medical monitor will review the AEs reported regularly and clarify with SI if there are queries. The data manager will review all AEs for consistency and provide summary of AEs to the medical monitor periodically. Non-clinically significant AEs still ongoing as the end of the study will be listed as continuing. SAEs continuing at the end of the study will be followed to resolution or stabilization. Details of AE reporting are included in the MOP.

The PI, SI, and site staff will exercise due diligence in ascertaining, accurately recording and promptly entering data on the eCRF for all AEs of all study participants. As data becomes available from the participant, the clinic and laboratories, adverse events should be recorded and entered by the site staff on regular basis. Site investigators will review, in a timely manner, the AE source data and determine the severity of the event and relation to the study agent. Site investigators will contact the study medical monitor for consultation of AEs as required.

### 9.4.2 SERIOUS ADVERSE EVENT REPORTING

The SI will complete a SAE Form within the following time frame:

- All SAEs will be recorded on the SAE Form and submitted by the site PI to the Overall study PI/Sponsor within 48 hours of initial receipt of the information (weekends and holidays are not included) and addressed to:

  Dr. Birkneh Tilahun Tadesse

  Study Medical Monitor

  International Vaccine Institute
  SNU Research Park, 1 Gwanak-ro, Gwanak-gu,
  Seoul, 08826 Republic of Korea,
  Phone: +82-2-881-1231
  Fax: +82-2-881-1228
  Mobile: +82-10-9804-1348
  Birkneh.Tadesse@ivi.int

- PFDA to be notified within 7 calendar days (for death and life threatening cases) and 15 calendar days [for those SAE which are fulfilling the criteria of Suspected Unexpected Serious Adverse Reactions (SUSAR)] by the sponsor/overall study PI/ Designee within the awareness of and the rest of the SAEs can be reported with the annual report.

All information (which may include special investigations and treatment received) will be recorded on the SAE Form and submitted to PFDA and site specific IRB. All SAEs will be followed until satisfactory resolution or until the site investigator deems the event to be chronic or to be stable. Other supporting documentation of the event may be requested by the sponsor and should be provided as soon as possible. SAE reporting to IVI IRB on regular basis is not mandatory (except those are of SUSAR category) and will be reported with the annual renewal report.

- The sponsor will be responsible for notifying the IVI IRB of Suspected Unexpected Serious Adverse Reactions (SUSAR) within 24 hours of initial receipt of the information (weekends and holidays are not included)

The SUSAR report will include the following information:

- It is mandatory to include an identifiable patient, an identifiable reporter, a suspect drug, and  an adverse event
- Protocol information: protocol number and date
- A detailed description of the event, incident, experience, or outcome
- An explanation of the basis for determining that the event, incident, experience, or outcome represents an unexpected problem

#### 9.4.3 SAFETY OVERSIGHT

An internal Safety Monitoring Committee (SMC) will be responsible to oversee the vaccine safety patterns during the course of the clinical trial. The SMC will be composed of individuals with appropriate expertise, including at least one pediatrician. The SMC will review blinded safety data on a regular basis according to the guidelines of the SMC charter. The SMC will send a summary of safety review findings to the site PIs and the Study Medical Monitor.

An independent Data Safety Monitoring Board (DSMB) will be constituted of experts from various fields of Medicine external to sponsor’s organization. The DSMB will oversee the study in terms of safety data as per DSMB charter. DSMB will have the authority to halt or terminate the study in case of any safety signals. DSMB chair will issue a recommendation letter after each meeting.

# 10 STUDY MONITORING

Study monitoring and auditing will be performed in accordance with the sponsor's procedures, GCP guidelines and any other applicable regulatory requirements.

Upon successful approval of the protocol and establishment of the Regulatory File, the clinical monitor will establish a clinical monitoring plan (CMP). To ensure that the investigator and the study staff understand and accept their defined responsibilities, the clinical monitor will maintain regular correspondence with the site and may be present during the course of the study to verify the acceptability of the facilities, compliance with the investigational plan and relevant regulations, and the maintenance of complete records.

Investigators and/or their study staff will be trained on the study protocol and all applicable study procedures prior to study initiation. Electronic CRFs supplied by the sponsor must be completed for each enrolled participant. The data entries as well as study related documents will be checked by the sponsor and/or trained delegates of the sponsor.

Study progress will be monitored by IVI study team or representative (e.g., a contract research organization) as frequently as necessary to ensure the rights and well-being of study participants are protected; to verify adequate, accurate and complete data collection; protocol compliance and to determine that the study is being conducted in conformance with applicable regulatory requirements. Arrangements for monitoring visits will be made in advance in accordance with the monitoring plan, except in case of emergency.

# 11 STATISTICAL CONSIDERATIONS

## 11.1 SAMPLE SIZE

A total of 1800 participants (1500 in Vi-DT test arm and 300 in control arm) aged 6 months to 45 years will be enrolled in this study. Participants will be randomized equally into first 2 groups of 750 participants each and third group with 300 participants within each age strata of 6 months to less than 2 years, 2 to less than 18 years and 18 to 45 years.

The sample size of two Vi-DT groups is decided based for the immunogenicity equivalence and safety data requirements. The immunogenicity subset in adult participants, N=250 per group,

will provide  94% power to show equivalence of geometric mean titres (GMT) of anti-Vi IgG at 4 weeks (28 days) after vaccination of Vi-DT (MD) and Vi-DT (SD) , with the equivalence margin of [0.67, 1.5] (WHO TRS 924). Coefficient of variation (CV) of immunogenicity titre is conservatively assumed as 2.0 based on IVI T001 and T002 studies at type 1 error rate of 0.05, and 10% drop out rate is also assumed.  This sample size of N=250 will provide 95% power, for equivalence tests of seroconversion rate between two formulations of Vi-DT with equivalence margin of [-10%, 10%]. In this calculation, 90% of sero-conversion rate in Vi-DT is assumed (based on IVI T001) with type 1 error rate of 0.05. The sample size of N=300 control (Meningococcal vaccine) is calculated to observe at least one events with 1% of the upper limit of 95% CI for any adverse event of incidence according to rule of three.

## 11.2 STATISTICAL ANALYSIS PLAN

The statistical analysis will focus on comparisons of immunogenicity of Vi-DT (MD; Group A) and Vi-DT (SD; Group B) at 4 weeks post vaccination. Safety of Vi-DT will be assessed by descriptively comparing incidence of common solicited, unsolicited and serious AE between Vi-DT and Control and any incidence of unexpected AE. A primary analysis will be performed after all participants complete week 4 visit post test/control vaccine dose in order to  initiate the test vaccine licensure process. Immunogenicity and safety data up to week 4 will be included in this analysis and this will be done in a way so that the study and study personnel remain blinded to the allocation of test/control vaccine until the end of the study. The final analysis will be performed when all participants complete week 24 visit. Immunogenicity and safety data up to week 24 will be included in the final analysis.
Additional statistical analysis details will be described in the statistical analysis plan (SAP) (which will be finalized prior to database lock) and any deviation(s) from the original SAP will be described and justified in the final study report.

## 11.3 STATISTICAL HYPOTHESES

The statistical hypothese for the primary objective is to demonstrate equivalence of two Vi-DT formulations (MD vs. SD) using GMT.

- Anti-Vi IgG GMT at 4 weeks (28 days) post Vi-DT (MD) is equivalent to GMT of Vi-DT (SD) in adults using equivalence margin of GMT ratio of [0.67, 1.5]

If the two-tailed 95% confidence interval of the ratio of GMT estimate of Vi-DT(MD) over GMT of Vi-DT(SD) is located within [0.67, 1.5], Vi-DT(MD) is equivalent to Vi-DT(SD) in terms of GMT of immunogenicity with two sided significance level of 0.05.

The statistical hypothese for the secondary objective is to demonstrate equivalence of two Vi-DT formulations (MD vs. SD) using seroconversion.

- Seroconversion rates of anti-Vi IgG ELISA antibody titres at 4 weeks (28 days) from baseline(D0) of Vi-DT(MD) is equivalent to seroconversion rate at 4 weeks of Vi- DT(SD) in adults using equivalence margin of 10%

If the two tailed 95% confidence interval of the estimate of difference of seroconversion rate between Vi-DT(MD) and Vi-DT(SD) at 4 weeks (Day 28) is located within [-10%, 10%], Vi-DT(MD) is equivalent to Vi-DT(SD) in terms of sero-conversion rate which is defined as 4 fold increase of immunogenicity from baseline with significance level of 0.05.

## 11.4 ANALYSIS DATASETS

The Full Analysis set (FAS) is a modified intention-to-treat (m-ITT) analysis set that will include all participants randomized in the study who received at least one dose of investigational vaccines. This data set will be used for demographic information and safety analysis.

The immunogenicity analysis set is a subset of FAS of those who is randomized, received at least one dose of investigational vaccines and have at least one post-baseline immunogenicity data available.

The per-protocol (PP) analysis set will be a subset of the immunogenicity analysis set who do not have protocol violations (defined as major deviation from the protocol compromising the scientific integrity of the study) with regards to the inclusion/exclusion criteria, are compliant with study procedures, completed all visits as scheduled and received the correct vaccinations.

The immunogenicity analysis set will be used for the primary analysis of the immunogenicity endpoints. A sensitivity analysis using the PP analysis sets will be conducted for the primary and secondary immunogenicity endpoints.

## 11.5 DESCRIPTION OF STATISTICAL METHODS

### 11.5.1 GENERAL APPROACH

This study is a randomized, observer-blinded phase III study in healthy participants with age 6 months to 45 years old at the time of vaccination of investigational vaccine to assess the safety and the equivalence of immunogenicity of multi-dose formulation compared to single-dose formulation of Vi-DT in an adult participants (18 -45 year age stratum).

Unless specified as in section 11.3, for equivalence test, the significance level is 5% with two sided test.

Analysis of covariance will be used to adjust for baseline titers, stratification and imbalances in baseline characteristics if necessary.

Missing immunogenicity data will not be imputed for the analysis. If missing data is more than 10%, the analysis of missing pattern will be assessed and a multiple imputation technique will be utilized as a sensitivity analysis.

### 11.5.2 BASELINE DESCRIPTIVE STATISTICS

Demographic characteristics and other baseline data of participants enrolled will be tabulated by vaccine group and overall. Continuous variables such as age, height and weight will be summarized by number of participants, mean, standard deviation, median, minimum and maximum. Categorical variables such as sex will be summarized by frequency and percentage in each vaccine. If a difference in baseline characteristics among groups is suspected, the statistical significance will be compared using ANOVA for continuous variables, and Chi-square test or Fisher's exact test for categorical variables.

### 11.5.3 SAFETY ANALYSIS

The following safety endpoints will be descriptively summarized by each formulation and overall and within each age stratum.

- Local and systemic solicited adverse events during the 7 days after each dose:

    - the solicited local reactions at the site of injection: pain, tenderness, erythema/redness, swelling/ induration, pruritus and
    - solicited Systemic reactions (adapted to each age group): fever, lethargy, irritability, nausea/vomiting, arthralgia, diarrhea, drowsiness, loss of appetite, chills, headache, fatigue, myalgia and persistent crying
- Unsolicited adverse events during 4 weeks (28 days) after vaccination
- Serious Adverse Events during the entire study period

Number of AEs and proportion of participants with safety endpoints after Vi-DT (MD), Vi-DT (SD), and Control vaccine will be summarized and the 95% confidence interval of the proportion will be calculated for Vi-DT (MD), Vi-DT (SD), and Control vaccine within each age stratum as well as overall strata.
Occurrence of any SAE during the study and AE that lead participant early drop out of the study will be listed.

### 11.5.4 ANALYSIS OF THE PRIMARY IMMUNOGENICITY ENDPOINT(S)

The primary immunogenicity endpoint will be measured as geometric mean titer (GMT) of anti-Vi IgG of Vi-DT (MD; Group A) and Vi-DT (SD; Group B) after 4 weeks of vaccination for equivalence comparison.

To assess the equivalence, the test between two different formulations on GMT of Vi-DT at 4 weeks will be performed with significance level of 0.05. The equivalence of anti-Vi GMT at 4 weeks post vaccination of Vi-DT between two different formulations will be analyzed using an analysis of covariance model with group and strata as covariates after log transformation. The equivalence of two formulations will be confirmed if the both limits of two-tailed 95% confidence interval of the ratio of GMT between two formulations of Vi-DT is within the equivalence margin of [0.67, 1.5].

### 11.5.5 ANALYSIS OF THE SECONDARY IMMUNOGENICITY ENDPOINT(S)

The secondary immunogenicity endpoint will be measured as seroconversion rate of Vi-DT (MD) and Vi-DT (SD) after 4 weeks of vaccination for equivalence comparison.

For assessment of seroconversion rate, the proportion of participants with at least 4-fold rise anti-Vi IgG ELISA antibody titer at  4 weeks as compared to prior to the investigational product dosing (Day 0) will be calculated. The equivalence of anti-Vi seroconversion at 4 weeks post vaccination of Vi-DT between two different formulations will be analyzed using the generalized linear model for binomial distribution with group and covariates. The equivalence of two formulations will be confirmed if both limits of two-tailed 95% confidence interval of the difference of seroconvergence between two formulations of Vi-DT is within the equivalence margin of [-10%, 10%].

#### 11.5.6 ADHERENCE AND RETENTION ANALYSES

Summaries of Participants Disposition will be based on full analysis set (FAS). A flow diagram of participant disposition (CONSORT flow diagram) will illustrate the progress of participants through the study duration from initial screening for eligibility to the completion of the primary outcome assessment. Number and percentage by vaccine group will be given for participants in the FAS, immunogenicity analysis set and PP analysis sets, and reasons for study discontinuation.

#### 11.5.7 PLANNED INTERIM ANALYSIS

A primary analysis will be performed after all participants complete week 4 visit post test/control vaccine dose. After that the final analysis will be performed when all participants complete week 24 visit. There will be two CSRs planned i.e. pCSR for the primary analysis and fCSR for final analysis.

#### 11.5.8 ADDITIONAL SUB-GROUP ANALYSIS

Potential difference in safety by ‘sex’ and ‘age strata’ and immunogenicity only in terms of ‘sex’ may be investigated.

#### 11.5.9 MULTIPLE COMPARISON/MULTIPLICITY

The primary comparison for equivalence will be tested with significant level of 0.05. No multiplicity adjustment for other comparisons.

# 12 SOURCE DOCUMENTS AND ACCESS TO SOURCE DOCUMENTS

Data recorded on the electronic Case Report Forms (eCRF) will be verified by checking the eCRF entries against source documents (i.e., all original records, laboratory reports, medical records, diary cards, memory aids, etc.) in order to ensure data completeness and accuracy as required by study protocol. Source documents will be stored at the clinical site in a secured place under lock and key. The investigator and/or site staff must make eCRFs and source documents of participants enrolled in this study available for inspection by IVI clinical team, clinical research associate (CRA) or its representative at the time of each monitoring visit.

At a minimum, source documentation must be available to substantiate participant identification, eligibility and participation, proper informed consent procedures, dates of visits, adherence to protocol procedures, adequate reporting and follow-up of adverse events, administration of concomitant medication, study vaccine receipt/dispensing/return records, study vaccine administration information, and date of completion and reason. Specific items required as source documents will be reviewed with the investigators before the study.

The source documents must also be available for inspection, verification and copying, as required by regulations, by officials of the regulatory health authorities (e.g., PFDA, others) and/or site IRBs and for possible audit by IVI Quality Management, Regulatory agency, notified body and collaboratorys/donors (e.g., Bill and Malinda Gates Foundation). The investigator and study site staff must comply with applicable privacy, data protection and medical confidentiality laws for use and disclosure of information related to the study and enrolled participants.

The participant must also allow access to  medical records. Each participant should be informed of this prior to the start of the study by administration of the inform consent process per ICH E6 (R2).

Each participant will have a complete source documentation of records including study log books, ICF, lab reports and test results for the entire study period. Appropriate source documents will be prepared by study staffs. These records must be available to the IVI and regulatory authorities upon request for review.

# 13 DATA HANDLING AND RECORD KEEPING

## 13.1 DATA COLLECTION AND MANAGEMENT RESPONSIBILITIES

Electronic Case Report Forms (eCRF) will be used for recording data for each participant enrolled in the study. The site investigators are responsible to ensure the accuracy, completeness, legibility and timeliness of the data captured in eCRF. Data captured in the eCRF derived from source documents and should remain consistent with those source documents. In case of discrepancies, data will be clarified and corrected. IVI will provide guidance to investigator on making corrections to the eCRF.
Study staff will extract all data collected in source documents and work books for computerization into the eCRF. Data will be entered into the eCRFs designed on Rave EDC. The entire data collection and handling will be monitored through the implementation of individual credentials to maintain appropriate database access and ensure database integrity. Edit checks will be programmed in the EDC to identify data entry errors during transcription, including range and consistency checks wherever applicable.

All sequential changes made will be captured in the audit trail in the EDC which will also provide error reports and summary reports for each activity. Data entry and cleaning will be conducted at the sites. Final data cleaning, data freezing and data analysis will be performed at the IVI. Unblinding of study vaccines will be carried out after database lock. All data will be stored in a secure data base maintained by Medidata Rave.

## 13.2 STUDY RECORDS RETENTION

The site Investigators (SI) will retain all study records that support eCRFs for this study (ie., ICFs, source documents, IP dispensing records)  required by sponsor and by the applicable regulations in a secure and safe facility. The SI will consult IVI representative before disposal of any study records, and will notify the sponsor of any change in the location, disposition, or custody of the study files. These documents should be retained for not less than 3 year after the approval of a marketing application or at least 2 years has been elapsed since formal discontinuation of clinical development of the investigational product. (ICH E6 (R2), 4.9.5). IVI

will inform the SI as to when these documents no longer need to be retained (ICH E6 (R2), 5.5.12).

If a PI retires, relocates or for other reasons withdraws from the responsibility of keeping the study records, custody must be transferred to a qualified person who will accept this responsibility. The Sponsor must be notified in writing of the name and address of the new custodian. The PI will be responsible for retaining sufficient information about each subject, i.e., name, address, telephone number, and subject identifier in the study, so that the sponsor and/or other regulatory authorities may have access to this information should the need arise.

### 13.3 PUBLICATION AND DATA SHARING POLICY

IVI assures that the key design elements of this protocol will be posted in a publicly accessible database such as Clinicaltrials.gov. All data collected during this study will be used to support this vaccine development plan until licensure and WHO prequalification. All individual data will stay strictly confidential. Analyzed data may be presented in scientific conferences, and published in peer-reviewed scientific journals. IVI reserves the right for overall study results publication. Any abstract, presentation and manuscript must be shared sufficiently in advance for proper review and approval as per IVI procedures. Anyone wishing to publish or present site-specific data obtained during and/or after completion of the study will conform to study site and IVI data sharing policies and then forward the publication and/or presentation for review and approval by IVI.

## 14 QUALITY ASSURANCE AND QUALITY CONTROL

Quality Assurance (QA) oversight will be required at all stages of the trial process per ICH E6 (R2) section 5.0 and/or local government GCP requirements.

Quality Control (QC) procedures will be implemented beginning with the data entry system and data QC checks that will be run on the database will be generated. Any missing data or data anomalies will be communicated to the site(s) for clarification/resolution.

During study conduct, the Sponsor or its designee (e.g., CRO) will conduct periodic monitoring visits (i.e., QC checks) to ensure that the protocol, Good Clinical Practice, local regulatory requirements and sponsor’s controlled documents (e.g., Standard Operating Procedure) are being followed. The monitors will review source documents to confirm that the data recorded on CRFs/eCRFs are accurate.

In addition to on-going QA oversight, selected investigator sites will be subjected to quality assurance audits performed by the sponsor or its designee, and/or by inspection by regulatory authorities and/or notified bodies.

The investigational sites will provide direct access to all study related sites, source data/documents, and reports for the purpose of monitoring and auditing by the sponsor; inspection by local and regulatory authorities and/or notified bodies.

The investigational sites will provide direct access to all study related sites, source data/documents, and reports for the purpose of monitoring and auditing by the sponsor, and inspection by local and regulatory authorities and/or notified bodies.

# 15 ETHICS/PROTECTION OF HUMAN PARTICIPANTS

## 15.1 REGULATORY AND ETHICAL COMPLIANCE

The investigators will ensure that this study is conducted in full conformity with the ICH E6 (R2) and E11 GCP Guidelines, Council for International Organizations of Medical Science (CIOMS), local country's ethical policy statement or the Declaration of Helsinki, whichever provides the most protection to human participants.

## 15.2 PARTICIPANT AND DATA CONFIDENTIALITY

Researchers shall adhere to the principles of transparency, legitimate purpose, and proportionality in the collection, retention, and processing of personal information (Philippines' Data Privacy Act of 2012).

Participant confidentiality is strictly held in trust by the participating investigators, their staff, and the sponsor and their agents. This confidentiality is extended to cover testing of biological samples in addition to the clinical information relating to participants. Therefore, the study protocol, documentation, data, and all other information generated will be held in strict confidentiality. No information concerning the study or the data will be released to any unauthorized third party without prior written approval of the sponsor.

The study monitor, other authorized representatives of the sponsor, representatives of the IRB

or pharmaceutical company supplying study product may inspect all documents and records required to be maintained by the investigator, including but not limited to, medical records (office, clinic, or hospital) and pharmacy records for the participants in this study. The clinical study site will permit access to such records.

The study participant's contact information will be securely stored at clinical site for internal use during the study. At the end of the study, all records will continue to be kept in a secure location for as long a period as dictated by local IRB and local regulations.

Individual participants and their research data will be identified by a unique study identification number. The study data entry and study management systems used by clinical site and by IVI/Zifo's Data Management will be secured and password-protected.

Researchers must respect participants' right to privacy. Unless required by law, the confidentiality of information shall at all times be observed. Records that link individuals to specific information shall not be released. No personal identifier will be used in any publication or communication used to support this research study. The participant's identification number will be used in the event it becomes necessary to identify data specific to a single participant.

## 15.3. RESEARCH USE OF STORED HUMAN SAMPLES

- Intended Use: Samples and data collected under this protocol may be used to study immune responses to the vaccines administered and for safety purpose if deemed necessary per medical judgement of the SI or special request from the sponsor or IRBs. No genetic testing will be performed
- Storage: Samples and data will be stored at respective sites and IVI using codes assigned by the investigators. Data will be kept in password-protected computers. Only investigators will have access to the stored samples and data
- Disposition at the completion of the study: All stored samples will be sent to IVI and be stored for 10 years after completion of CSR or according to subject's decision about preservation period
- Study participants who request destruction of samples will be notified of compliance with such request and all supporting details will be maintained for tracking

## 15.4 FUTURE USE OF STORED SPECIMENS

With the participant's approval (consent form) and as approved by PFDA, local sites and IVI IRBs, the identified biological samples will be stored at the immunology lab in IVI for future use. The immunology lab at IVI will be attributed a code that will allow linking the biological specimens with the phenotypic data from each participant, maintaining the masking of the identity of the participants.

During the conduct of the study, an individual participant can choose to withdraw consent to have biological specimens stored for future research.

The stored samples may be used for additional assessment of typhoid immunogenicity, study of possible immune correlates of protection, validation of assays, testing of new assays, and for safety purpose.

# 16 REFERENCES


1. SAGE Working Group on Typhoid Vaccines and the WHO Secretariat., *Background paper to SAGE on typhoid vaccine policy recommendations.* September 2017.
2. John, J., C.J. Van Aart, and N.C. Grassly, *The Burden of Typhoid and Paratyphoid in India: Systematic Review and Meta-analysis.* PLoS Negl Trop Dis, 2016. **10**(4): p. e0004616.
3. Buckle, G.C., C.L. Walker, and R.E. Black, *Typhoid fever and paratyphoid fever: Systematic review to estimate global morbidity and mortality for 2010.* J Glob Health, 2012. **2**(1): p. 010401.
4. Crump, J.A., S.P. Luby, and E.D. Mintz, *The global burden of typhoid fever.* Bull World Health Organ, 2004. **82**(5): p. 346-53.
5. Mogasale, V., et al., *Burden of typhoid fever in low-income and middle-income countries: a systematic, literature-based update with risk-factor adjustment.* Lancet Glob Health, 2014. **2**(10): p. e570-80.
6. Marks, F., et al., *Incidence of invasive salmonella disease in sub-Saharan Africa: a multicentre population-based surveillance study.* Lancet Glob Health, 2017. **5**(3): p. e310-e323.
7. McClelland, M., et al., *Complete genome sequence of Salmonella enterica serovar Typhimurium LT2.* Nature, 2001. **413**(6858): p. 852-6.
8. Bhan, M.K., R. Bahl, and S. Bhatnagar, *Typhoid and paratyphoid fever.* Lancet, 2005. **366**(9487): p. 749-62.
9. Hornick, R.B., et al., *Typhoid fever: pathogenesis and immunologic control. 2.* N Engl J Med, 1970. **283**(14): p. 739-46.
10. Hornick, R.B., et al., *Typhoid fever: pathogenesis and immunologic control.* N Engl J Med, 1970. **283**(13): p. 686-91.
11. World Health Organization. *Typhoid vaccines: WHO position paper- March 2018*. March 2018 [cited 2019 June 4]; Available from: *https://apps.who.int/iris/bitstream/handle/10665/272272/WER9313.pdf?ua=1*.(Assessed in Oct 2019)
12. DeRoeck, D., L. Jodar, and J. Clemens, *Putting typhoid vaccination on the global health agenda.* N Engl J Med, 2007. **357**(11): p. 1069-71.
13. Levine, M.M., et al., *Large-scale field trial of Ty21a live oral typhoid vaccine in enteric-coated capsule formulation.* Lancet, 1987. **1**(8541): p. 1049-52.
14. Klugman, K.P., et al., *Immunogenicity, efficacy and serological correlate of protection of Salmonella typhi Vi capsular polysaccharide vaccine three years after immunization.* Vaccine, 1996. **14**(5): p. 435-8.
15. Szu, S.C., et al., *Laboratory and preliminary clinical characterization of Vi capsular polysaccharide-protein conjugate vaccines.* Infect Immun, 1994. **62**(10): p. 4440-4.
16. Thiem, V.D., et al., *The Vi conjugate typhoid vaccine is safe, elicits protective levels of IgG anti-Vi, and is compatible with routine infant vaccines.* Clin Vaccine Immunol, 2011. **18**(5): p. 730-5.
17. Micoli, F., et al., *Vi-CRM 197 as a new conjugate vaccine against Salmonella Typhi.* Vaccine, 2011. **29**(4): p. 712-20.
18. van Damme, P., et al., *Safety, immunogenicity and dose ranging of a new Vi-CRM(1)(9)(7) conjugate vaccine against typhoid fever: randomized clinical testing in healthy adults.* PLoS One, 2011. **6**(9): p. e25398.

19. Mitra, M., et al., *Efficacy and safety of vi-tetanus toxoid conjugated typhoid vaccine (PedaTyph) in Indian children: School based cluster randomized study.* Hum Vaccin Immunother, 2016. **12**(4): p. 939-45.
20. Mohan, V.K., et al., *Safety and immunogenicity of a Vi polysaccharide-tetanus toxoid conjugate vaccine (Typbar-TCV) in healthy infants, children, and adults in typhoid endemic areas: a multicenter, 2-cohort, open-label, double-blind, randomized controlled phase 3 study.* Clin Infect Dis, 2015. **61**(3): p. 393-402.
21. Capeding, M.R., et al., *Safety and immunogenicity of a Vi-DT typhoid conjugate vaccine: Phase I trial in Healthy Filipino adults and children.* Vaccine, 2018. **36**(26): p. 3794-3801.
22. Cadoz, M., *Potential and limitations of polysaccharide vaccines in infancy.* Vaccine, 1998. **16**(14-15): p. 1391-5.
23. Kanungo, S., S. Dutta, and D. Sur, *Epidemiology of typhoid and paratyphoid fever in India.* J Infect Dev Ctries, 2008. **2**(6): p. 454-60.
24. Engels, E.A. and J. Lau, *Vaccines for preventing typhoid fever.* Cochrane Database Syst Rev, 2000(2): p. Cd001261.
25. Bhutta, Z.A., et al., *Immunogenicity and safety of the Vi-CRM197 conjugate vaccine against typhoid fever in adults, children, and infants in south and southeast Asia: results from two randomised, observer-blind, age de-escalation, phase 2 trials.* Lancet Infect Dis, 2014. **14**(2): p. 119-29.
26. Canh, D.G., et al., *Effect of dosage on immunogenicity of a Vi conjugate vaccine injected twice into 2- to 5-year-old Vietnamese children.* Infect Immun, 2004. **72**(11): p. 6586-8.
27. Clinical Trials Branch, Health Products Regulation Group, and Health Sciences Authority, *Clinical trials guidance, Labelling of therapeutic products and medical products used in clinical trials*. May 2017, Health Sciences Authority: Singapore.
28. U.S. Department of Health and Human Services, et al. *Division of AIDS (DAIDS) Table for Grading the Severity of Adult and Pediatric Adverse Events, Version 2.0.* . November 2014 [cited 2019 June 4]; Available from: *https://rsc.niaid.nih.gov/sites/default/files/daids-ae-grading-table-v2-nov2014.pdf*. (Assessed in Oct 2019)

# 17 APPENDICES

Appendix i. Statement of compliance

Appendix ii. Vi-DT Phase II Primary Clinical Study Report Summary

**Appendix-i**

## STATEMENT OF COMPLIANCE

1) The study will be conducted according to the protocol and in compliance with International Council for Harmonization (ICH) Good Clinical Practice (GCP), Belmont Principles, CIOMS guidelines, Declaration of Helsinki and other applicable regulations in the Republic of the Philippines (Food and Drug Administration of the Republic of the Philippines) and sponsor requirement. The Principal Investigators will ensure that no deviation from, or changes to the protocol will take place without prior agreement from the sponsor and documented approval from the Institutional Review Boards (IRBs), except where necessary to eliminate an immediate hazard(s) to the trial participants. The protocol, informed consent form(s), recruitment materials, and all participant materials will be submitted to the IRBs for review and approval. All identified study personnel will be trained to perform their roles and will carry out their responsibilities in accordance with ICH GCP guideline and clinic site SOPs. Roles and responsibilities of study staff are presented in the Manual of Procedures.

I agree to ensure that all staff members involved in the conduct of this study are informed about their obligations in meeting the above commitments.

| | |
|---|---|
| Principal Investigator: | **Dr. Michelle C. Ylade, MD, MSc**<br>Research Assistant Professor 1<br>University of the Philippines Manila-National Institutes of Health,<br>623 Pedro Gil St. Ermita, Manila 1000,<br>Philippines |
| | Print/Type Name |

Signed: ______________________ Date: ______________________

Signature

2) The study will be conducted according to the protocol and in compliance with International Council for Harmonization (ICH) Good Clinical Practice (GCP), Belmont Principles, CIOMS guidelines, Declaration of Helsinki and other applicable regulations in the Republic of the Philippines (Food and Drug Administration of the Republic of the Philippines) and sponsor requirement. The Principal Investigators will ensure that no deviation from, or changes to the protocol will take place without prior agreement from the sponsor and documented approval from the Institutional Review Boards (IRBs), except where necessary to eliminate an immediate hazard(s) to the trial participants. The protocol, informed consent form(s), recruitment materials, and all participant materials will be submitted to the IRBs for review and approval. All identified study personnel will be trained to perform their roles and will carry out their responsibilities in accordance with ICH GCP guideline and clinic site SOPs. Roles and responsibilities of study staff are presented in the Manual of Procedures.

I agree to ensure that all staff members involved in the conduct of this study are informed about their obligations in meeting the above commitments.

| | |
|---|---|
| Coordinating Investigator cum Principal Investigator: | **Dr. Josefina Cadorna Carlos, MD**<br>Professor (Pediatrics/Infectious Diseases)<br>University of the East-Ramon Magsaysay Memorial Medical Center Inc.<br>64 Aurora Blvd., Brgy. Imelda, Quezon City 1113<br>Philippines<br>____________________________<br>Print/Type Name |

Signed: ____________________ Date: ____________________

Signature

3) The study will be conducted according to the protocol and in compliance with International Council for Harmonization (ICH) Good Clinical Practice (GCP), Belmont Principles, CIOMS guidelines, Declaration of Helsinki and other applicable regulations in the Republic of the Philippines (Food and Drug Administration of the Republic of the Philippines) and sponsor requirement. The Principal Investigators will ensure that no deviation from, or changes to the protocol will take place without prior agreement from the sponsor and documented approval from the Institutional Review Boards (IRBs), except where necessary to eliminate an immediate hazard(s) to the trial participants. The protocol, informed consent form(s), recruitment materials, and all participant materials will be submitted to the IRBs for review and approval. All identified study personnel will be trained to perform their roles and will carry out their responsibilities in accordance with ICH GCP guideline and clinic site SOPs. Roles and responsibilities of study staff are presented in the Manual of Procedures.

I agree to ensure that all staff members involved in the conduct of this study are informed about their obligations in meeting the above commitments.

| | |
|---|---|
| Principal Investigator: | **Dr. Charissa Borja-Tabora, MD, FPPS, FPIDSP**<br>Medical Specialist III<br>ASIAN HOSPITAL AND MEDICAL CENTER<br>2205 Civic Dr, Alabang, Muntinlupa 1780, Metro Manila, Philippines |
| | ______________________________<br>Print/Type Name |

Signed: ____________________ Date: ____________________

Signature

4) The study will be conducted according to the protocol and in compliance with International Council for Harmonization (ICH) Good Clinical Practice (GCP), Belmont Principles, CIOMS guidelines, Declaration of Helsinki and other applicable regulations in the Republic of the Philippines (Food and Drug Administration of the Republic of the Philippines) and sponsor requirement. The Principal Investigators will ensure that no deviation from, or changes to the protocol will take place without prior agreement from the sponsor and documented approval from the Institutional Review Boards (IRBs), except where necessary to eliminate an immediate hazard(s) to the trial participants. The protocol, informed consent form(s), recruitment materials, and all participant materials will be submitted to the IRBs for review and approval. All identified study personnel will be trained to perform their roles and will carry out their responsibilities in accordance with ICH GCP guideline and clinic site SOPs. Roles and responsibilities of study staff are presented in the Manual of Procedures.

I agree to ensure that all staff members involved in the conduct of this study are informed about their obligations in meeting the above commitments.

| | |
|---|---|
| Principal Investigator: | **Dr. Edison Alberto, MD**<br>Research Consultant<br>Medical Research Unit<br>Tropical Disease Foundation, Inc.<br>Room 2002, Medical Plaza, Amorsolo corner Dela Rosa Street,<br>Legaspi Village, Makati City 1229, Metro Manila,<br>Philippines |
| | ______________________________<br>Print/Type Name |

Signed: ______________________ Date: ______________________

Signature

## Appendix-ii

## VI-DT PHASE II PRIMARY CLINICAL STUDY REPORT SUMMARY

***Immunogenicity end points are as follows***:

Study met primary the immunogenicity end point i.e. Vi-DT combined vaccine group is superior to placebo group by overall analysis and by age strata in terms of seroconversion rate (post 1st dose) at Week 4 and achieved both secondary immunogenicity endpoints such as Vi-DT two-dose vaccine group is superior to placebo group by overall analysis and by age strata in terms of seroconversion rate (post 2nd dose) at Week 28. The other immunogenicity end point is Vi-DT one-dose vaccine group at Week 4 post single dose is non-inferior to Vi-DT two-dose vaccine group at Week 4 post two-dose by overall analysis and by age strata. This analysis was done in terms of GMT at Week 4 in the single-dose group and at Week 28 in the two-dose group.

***Safety enpoints are as follows:***

As a part of immediate reaction one participant who received 1st dose of Vi-DT reported erythema/redness, fever within 30 min.

Solicited AEs: In post first dose, a higher percentage in Vi-DT combined group who experienced AEs compared to placebo group (25.88% vs. 19.30%) was observed. In post second dose, a lower percentage in Vi-DT two-dose group who experienced AEs compared to placebo (6.48% vs. 9.09%) was observed.

Unsolicited AEs: Within 4 weeks post post first dose, a lower percentage in Vi-DT combined group experienced AEs compared to Placebo (61.40% vs. 68.42%). Within 4 weeks post second dose, a higher percentage in Vi-DT two-dose group experienced AEs compared to placebo (30.56% vs. 27.27%).

Medically significant unsolicited AEs rates which happened beyond 4 weeks post each dose are lower in percentage in Vi-DT combined group compared to placebo group during the entire study period (22.81% vs. 38.60%).

In terms of SAE, comparable percentages of SAEs between Vi-DT combined group and placebo group within 4 weeks after either dose (0.88% vs. 1.75%) and during entire study period (4.39% vs. 5.26%) were reported.
