# Supplementary material for: A Phase 3, Multicenter, Randomized, Controlled Trial to Evaluate Immune Equivalence and Safety of Multidose and Single-dose Formulations of Vi-DT Typhoid Conjugate Vaccine in Healthy Filipino Individuals 6 Months to 45 Years of Age
Source: Lancet Reg Health West Pac. 2022 May 30;24:100484. doi: 10.1016/j.lanwpc.2022.100484 (PMC9160840; doi:10.1016/j.lanwpc.2022.100484)
Supplement: Supplementary file 2 [file mmc2.docx]

***Supplementary Material*:**

**A Phase 3, multicenter, randomized, controlled trial to evaluate immune equivalence and safety of multidose and single dose formulations of Vi-DT typhoid conjugate vaccine in healthy individuals 6 months to 45 years of age.**

**Contents:**

1. Supplementary Tables ---- Page 2
2. Statistical Analysis Plan ---Page 13

**SUPPLEMENTARY TABLES**

[Table S 1 *Exclusion Criteria from participation in the trial.* 3](#_Toc83848516)

[Table S 2 Formulation, Presentation and Batch numbers of Vaccines Administered 4](#_Toc83848517)

[Table S 3: Geometric mean titers of anti-Vi IgG using the Per Protocol Analysis Set in the adult age stratum. 5](#_Toc83848518)

[Table S 4 Seroconversion rates of anti-Vi IgG in the single dose, multidose and comparator groups using the Per Protocol Analysis Set in the adult age stratum. 6](#_Toc83848519)

[Table S 5: Summary of Adverse Events – Age Stratum 1: 18 to 45 years 7](#_Toc83848520)

[Table S 6: Summary of Adverse Events – Age Stratum 2: 2 to 18 years 9](#_Toc83848521)

[**Table S 7** Summary of Adverse Events – Age Stratum 3: 6 months to less than 2 years 11](#_Toc83848522)

Table S 1 *Exclusion Criteria from participation in the trial.*

| **Definite Exclusion Criteria** |
| --- |
| 1. Child with a congenital abnormality, 2. Participant who has already received meningococcal conjugate vaccine, 3. Subject concomitantly enrolled or scheduled to be enrolled in another trial, 4. Known history of immune function disorders including immunodeficiency diseases (known HIV infection or other immune function disorders), 5. Chronic use of systemic steroids (>2 mg/kg/day or >2   [Table S 1 *Exclusion Criteria from participation in the trial.* 3](#_Toc83848370)  [Table S 3 Formulation, Presentation and Batch numbers of Vaccines Administered 4](#_Toc83848371)  [Table S 4: Geometric mean titers of anti-Vi IgG using the Per Protocol Analysis Set in the adult age stratum. 5](#_Toc83848372)  [Table S 5 Seroconversion rates of anti-Vi IgG in the single dose, multidose and comparator groups using the Per Protocol Analysis Set in the adult age stratum. 6](#_Toc83848373)  [Table S 6: Summary of Adverse Events – Age Stratum 1: 18 to 45 years 7](#_Toc83848374)  [Table S 7: Summary of Adverse Events – Age Stratum 2: 2 to 18 years 9](#_Toc83848375)  [**Table S 8** Summary of Adverse Events – Age Stratum 3: 6 months to less than 2 years 11](#_Toc83848376)   1. 0 mg/day prednisone equivalent for periods exceeding 10 days), cytotoxic or other immunosuppressive drugs, 2. Receipt of blood or blood-derived products in the past 3 months, 3. Participant with a previously ascertained or suspected disease caused by S. Typhi (confirmed either clinically, serologically, or microbiologically), 4. Participant who has had household contact with/and or intimate exposure to an individual with laboratory-confirmed S. Typhi, 5. Individual who has previously received a typhoid vaccine, 6. Participant who has received other vaccines from 1 month prior to test vaccination or planned to receive any vaccine within 1 month (except a measles containing vaccine as per government vaccination campaign) 7. Known history or allergy to vaccines or other medications, 8. History of uncontrolled coagulopathy or blood disorders, 9. Any abnormality or chronic disease which in the opinion of the investigator might be detrimental for the safety of the subject and interfere with the assessment of the study objectives, 10. Any female participant who was lactating, pregnant* or planning for pregnancy during the study period, 11. Participants/parents/LAR planned to move from the study area before the end of study period, 12. As per Investigator’s medical judgement despite meeting all inclusion/exclusion criteria mentioned above.   * A UPT was necessary for all female participants of childbearing age from menarche. |
| **Temporary Contraindication:** |
| - Acute illness, in particular infectious disease, or fever (axillary temperature ≥37.5°C), within three days prior to enrollment and vaccination. These individuals could be rescreened upon resolution of condition. |

UPT – Urine Pregnancy Test

Table S 2 Formulation, Presentation and Batch numbers of Vaccines Administered

| Vaccine | Formulation | Presentation | Volume | Batch/ Lot number |
| --- | --- | --- | --- | --- |
| NBP618 MD Vi-DT candidate vaccine | Vi polysaccharide conjugated to diphtheria toxoid 25 μg  Di Sodium hydrogen phosphate 0.620mg  Sodium Dihydrogen Phosphate dihydrate 0.152 mg  Sodium chloride 4.25mg  Water for injection q.s.  Preservative: 2-phenoxy ethanol 5.0 mg | Clear, colorless liquid in 3mL multiple dose glass vial. (5 doses per each MD vial) | 0.5 mL per dose (total volume of vial 3 mL) | S121902 |
| NBP618 SD Vi-DT candidate vaccine | Vi polysaccharide conjugated to diphtheria toxoid 25 μg  Di Sodium hydrogen phosphate 0.620mg  Sodium Dihydrogen Phosphate dihydrate 0.152 mg  Sodium chloride 4.25mg  Water for injection q.s. | Clear colorless liquid presented in Type I glass vial | 0.5mL | CRSAV104 |
| Meningococcal conjugate vaccine (Nimenrix®) | Tetravalent meningococcal polysaccharide conjugated vaccine consisting of Neisseria meningitidis capsular polysaccharides A, C, W-135 and Y each coupled to a carrier protein | The diluent (sodium chloride and water for injections) is a sterile clear and colorless liquid supplied separately in a prefilled syringe/ ampoule. After reconstitution, vaccine appears as a clear and colorless solution. | 0.5mL | A90CA360A |

MD – Multidose; SD – Single dose

Table S 3: Geometric mean titers of anti-Vi IgG using the Per Protocol Analysis Set in the adult age stratum.

| **Time point** | **Vi-DT (MD)** | | **Vi-DT (SD)** | | **Control** | | **Vi-DT(MD) / Vi-DT(SD)** | **P-value** |
| --- | --- | --- | --- | --- | --- | --- | --- | --- |
|  | **N** | **GMT***  **(95% CI)** | **N** | **GMT***  **(95% CI)** | **N** | **GMT***  **(95% CI)** | **Ratio**  **(95% CI)†** |  |
| Day 0 | 246 | 2.15  (1.79, 2.59) | 252 | 1.77  (1.48, 2.11) | 103 | 2.16  (1.61, 2.91) | 1.22  (0.94, 1.57) | 0.1320[1]  0.2616[2]  0.9992[3]  0.3585[4] |
| Week 4 | 246 | 638.26  (544.11, 748.71) | 252 | 562.57  (478.80, 661.00) | 103 | 2.51  (1.77, 3.58) | 1.13  (0.90, 1.42) | 0.2736[1]  <0.0001[2]  <0.0001[3]  <0.0001[4] |

[Note] N: number of total participants; *Geometric Mean Titers (unit: IU/ml); CI: Confidence Interval

† The equivalence of a multidose (MD) formulation compared to a single dose (SD) was confirmed if both limits of two-tailed 95% confidence interval of the fold difference of GMT between two formulations of Vi-DT is within the equivalence margin of [0.67, 1.5].

[1] The P-value was derived from two sample t-test after log transformation between Vi-DT MD vs. SD.

[2] The P-value was derived from ANOVA among 3 groups (Vi-DT MD vs. SD vs. Control).

[3] The P-value was derived from Dunnett’s procedure that was followed to compare the Vi-DT MD vs. Control.

[4] The P-value was derived from Dunnett’s procedure that was followed to compare the Vi-DT SD vs. Control.

Table S 4 Seroconversion rates of anti-Vi IgG in the single dose, multidose and comparator groups using the Per Protocol Analysis Set in the adult age stratum.

| **Age Stratum 1: 18 to 45 years** | | | | | | | | |
| --- | --- | --- | --- | --- | --- | --- | --- | --- |
| **Time point** | **Vi-DT (MD)** | | **Vi-DT (SD)** | | **Control** | | **Vi-DT (MD) – Vi-DT (SD)** | **P-value** |
|  | **n/N** | **Seroconversion rate***  **(95% CI)** | **n/N** | **Seroconversion rate***  **(95% CI)** | **n/N** | **Seroconversion rate***  **(95% CI)** | **Difference (95% CI)^†^** |  |
| Week 4 | 242/246 | 98.37  (95.89, 99.37) | 249/252 | 98.81  (96.56, 99.59) | 6/103 | 5.83  (2.70, 12.13) | -0.44  (-4.43, 3.56) | 0.7217[1]  <0.0001[2]  <0.0001[3]  <0.0001[4] |

[Note] N: number of total participants; n: number of participants seroconverted.

* Proportion of participants who had at least 4-fold rise anti-Vi IgG ELISA antibody titers at 4 weeks after vaccination compared to baseline (Day 0; prior to vaccination).

† The equivalence of two formulations was confirmed if the both limits of two-tailed 95% confidence interval of the difference of seroconversion rate between two lots of Vi-DT was within the equivalence margin of [-10%, 10%].

[1] The P-value was derived from Fisher’s exact test between Vi-DT MD vs. SD.

[2] The P-value was derived from Chi-square test among 3 groups (Vi-DT MD vs. SD vs. Control).

[3] The P-value was derived from Chi-square test between Vi-DT MD vs. Control.

[4] The P-value was derived from Chi-square test between Vi-DT SD vs. Control.

Table S 5: Summary of Adverse Events – Age Stratum 1: 18 to 45 years

| **Entire study period** | **Vi-DT (MD)**  **(N=345)** | | **Vi-DT (SD)**  **(N=345)** | | **Control**  **(N=138)** | | **P-value** |
| --- | --- | --- | --- | --- | --- | --- | --- |
|  | **m** | **n (%)** | **m** | **n (%)** | **m** | **n (%)** |  |
| Immediate Reaction (for 30 minutes after vaccination) | 4 | 4 (1.16%) | 1 | 1 (0.29%) | 1 | 1 (0.72%) | 0.4101[2] |
| Severity: |  |  |  |  |  |  |  |
| Mild | 4 | 4 (1.16%) | 1 | 1 (0.29%) | 1 | 1 (0.72%) | 0.4101[2] |
| Moderate | 0 | 0 (0.00%) | 0 | 0 (0.00%) | 0 | 0 (0.00%) | NA |
| Severe | 0 | 0 (0.00%) | 0 | 0 (0.00%) | 0 | 0 (0.00%) | NA |
| Potentially life threatening | 0 | 0 (0.00%) | 0 | 0 (0.00%) | 0 | 0 (0.00%) | NA |
| Relatedness: |  |  |  |  |  |  |  |
| Definitely Related | 3 | 3 (0.87%) | 1 | 1 (0.29%) | 1 | 1 (0.72%) | 0.5971[2] |
| Probably Related | 1 | 1 (0.29%) | 0 | 0 (0.00%) | 0 | 0 (0.00%) | 1.0000[2] |
| Possibly related | 0 | 0 (0.00%) | 0 | 0 (0.00%) | 0 | 0 (0.00%) | NA |
| Unlikely related | 0 | 0 (0.00%) | 0 | 0 (0.00%) | 0 | 0 (0.00%) | NA |
| Not Related | 0 | 0 (0.00%) | 0 | 0 (0.00%) | 0 | 0 (0.00%) | NA |
| Solicited AE (During 7 days after vaccination) | 214 | 81 (23.48%) | 201 | 78 (22.61%) | 117 | 38 (27.54%) | 0.5086[1] |
| Severity: |  |  |  |  |  |  |  |
| Mild | 159 | 76 (22.03%) | 169 | 74 (21.45%) | 98 | 35 (25.36%) | 0.6365[1] |
| Moderate | 50 | 22 (6.38%) | 29 | 13 (3.77%) | 18 | 6 (4.35%) | 0.2695[1] |
| Severe | 5 | 3 (0.87%) | 3 | 2 (0.58%) | 1 | 1 (0.72%) | 1.0000[2] |
| Potentially life threatening | 0 | 0 (0.00%) | 0 | 0 (0.00%) | 0 | 0 (0.00%) | NA |
| Relatedness: |  |  |  |  |  |  |  |
| Definitely Related | 85 | 46 (13.33%) | 110 | 46 (13.33%) | 40 | 17 (12.32%) | 0.9495[1] |
| Probably Related | 86 | 33 (9.57%) | 62 | 31 (8.99%) | 43 | 12 (8.70%) | 0.9437[1] |
| Possibly related | 30 | 14 (4.06%) | 26 | 12 (3.48%) | 29 | 16 (11.59%) | 0.0006[1] |
| Unlikely related | 7 | 3 (0.87%) | 1 | 1 (0.29%) | 1 | 1 (0.72%) | 0.5971[2] |
| Not Related | 6 | 3 (0.87%) | 2 | 2 (0.58%) | 4 | 2 (1.45%) | 0.5403[2] |
| Solicited AEs related to vaccine: | 208 | 78 (22.61%) | 199 | 76 (22.03%) | NA | NA | 0.8549[1] |
| Severity: |  |  |  |  |  |  |  |
| Mild | 156 | 74 (21.45%) | 167 | 72 (20.87%) | NA | NA | 0.8521[1] |
| Moderate | 47 | 21 (6.09%) | 29 | 13 (3.77%) | NA | NA | 0.1594[1] |
| Severe | 5 | 3 (0.87%) | 3 | 2 (0.58%) | NA | NA | 1.0000[2] |
| Potentially life threatening | 0 | 0 (0.00%) | 0 | 0 (0.00%) | NA | NA | NA |
| Unsolicited AE (During 4 weeks (28 days) after vaccination) | 61 | 51 (14.78%) | 43 | 36 (10.43%) | 17 | 14 (10.14%) | 0.1575[1] |
| Severity: |  |  |  |  |  |  |  |
| Mild | 57 | 48 (13.91%) | 42 | 35 (10.14%) | 15 | 13 (9.42%) | 0.2067[1] |
| Moderate | 4 | 4 (1.16%) | 1 | 1 (0.29%) | 2 | 2 (1.45%) | 0.3019[2] |
| Severe | 0 | 0 (0.00%) | 0 | 0 (0.00%) | 0 | 0 (0.00%) | NA |
| Potentially life threatening | 0 | 0 (0.00%) | 0 | 0 (0.00%) | 0 | 0 (0.00%) | NA |
| Relatedness: |  |  |  |  |  |  |  |
| Definitely Related | 0 | 0 (0.00%) | 0 | 0 (0.00%) | 0 | 0 (0.00%) | NA |
| Probably Related | 2 | 2 (0.58%) | 1 | 1 (0.29%) | 0 | 0 (0.00%) | 1.0000[2] |
| Possibly related | 0 | 0 (0.00%) | 0 | 0 (0.00%) | 0 | 0 (0.00%) | NA |
| Unlikely related | 5 | 5 (1.45%) | 3 | 3 (0.87%) | 3 | 2 (1.45%) | 0.7593[2] |
| Not Related | 54 | 45 (13.04%) | 39 | 34 (9.86%) | 14 | 12 (8.70%) | 0.2613[1] |
| Unsolicited AEs related to vaccine: | 7 | 7 (2.03%) | 4 | 4 (1.16%) | NA | NA | 0.3619[1] |
| Severity: |  |  |  |  |  |  |  |
| Mild | 6 | 6 (1.74%) | 4 | 4 (1.16%) | NA | NA | 0.5241[1] |
| Moderate | 1 | 1 (0.29%) | 0 | 0 (0.00%) | NA | NA | 1.0000[2] |
| Severe | 0 | 0 (0.00%) | 0 | 0 (0.00%) | NA | NA | NA |
| Potentially life threatening | 0 | 0 (0.00%) | 0 | 0 (0.00%) | NA | NA | NA |
| SAE | 1 | 1 (0.29%) | 1 | 1 (0.29%) | NA | NA | 1.0000[2] |

[Note] N: number of total participants; n: number of participants who reported events; m: number of events; %: percentages (100*n/N). NA: Not Applicable.

The p-value was derived from [1] Chi-square test or [2] Fisher’s exact test compared among Vi-DT (MD), Vi-DT (SD), and Control.

Table S 6: Summary of Adverse Events – Age Stratum 2: 2 to 18 years

| **Entire study period** | **Vi-DT (MD)**  **(N=250)** | | **Vi-DT (SD)**  **(N=250)** | | **Control**  **(N=100)** | | **P-value** |
| --- | --- | --- | --- | --- | --- | --- | --- |
|  | **m** | **n (%)** | **m** | **n (%)** | **m** | **n (%)** |  |
| Immediate Reaction (for 30 minutes after vaccination) | 5 | 5 (2.00%) | 6 | 6 (2.40%) | 1 | 1 (1.00%) | 0.6997[1] |
| Severity: |  |  |  |  |  |  |  |
| Mild | 5 | 5 (2.00%) | 6 | 6 (2.40%) | 1 | 1 (1.00%) | 0.6997[1] |
| Moderate | 0 | 0 (0.00%) | 0 | 0 (0.00%) | 0 | 0 (0.00%) | NA |
| Severe | 0 | 0 (0.00%) | 0 | 0 (0.00%) | 0 | 0 (0.00%) | NA |
| Potentially life threatening | 0 | 0 (0.00%) | 0 | 0 (0.00%) | 0 | 0 (0.00%) | NA |
| Relatedness: |  |  |  |  |  |  |  |
| Definitely Related | 4 | 4 (1.60%) | 5 | 5 (2.00%) | 0 | 0 (0.00%) | 0.5948[2] |
| Probably Related | 1 | 1 (0.40%) | 1 | 1 (0.40%) | 1 | 1 (1.00%) | 0.5655[2] |
| Possibly related | 0 | 0 (0.00%) | 0 | 0 (0.00%) | 0 | 0 (0.00%) | NA |
| Unlikely related | 0 | 0 (0.00%) | 0 | 0 (0.00%) | 0 | 0 (0.00%) | NA |
| Not Related | 0 | 0 (0.00%) | 0 | 0 (0.00%) | 0 | 0 (0.00%) | NA |
| Solicited AE (During 7 days after vaccination) | 164 | 49 (19.60%) | 128 | 45 (18.00%) | 70 | 27 (27.00%) | 0.1589[1] |
| Severity: |  |  |  |  |  |  |  |
| Mild | 148 | 47 (18.80%) | 110 | 43 (17.20%) | 60 | 24 (24.00%) | 0.3401[1] |
| Moderate | 16 | 9 (3.60%) | 17 | 8 (3.20%) | 10 | 7 (7.00%) | 0.2388[1] |
| Severe | 0 | 0 (0.00%) | 1 | 1 (0.40%) | 0 | 0 (0.00%) | 1.0000[2] |
| Potentially life threatening | 0 | 0 (0.00%) | 0 | 0 (0.00%) | 0 | 0 (0.00%) | NA |
| Relatedness: |  |  |  |  |  |  |  |
| Definitely Related | 95 | 29 (11.60%) | 80 | 25 (10.00%) | 49 | 18 (18.00%) | 0.1111[1] |
| Probably Related | 43 | 23 (9.20%) | 28 | 18 (7.20%) | 16 | 7 (7.00%) | 0.6563[1] |
| Possibly related | 25 | 7 (2.80%) | 18 | 9 (3.60%) | 3 | 2 (2.00%) | 0.7092[1] |
| Unlikely related | 1 | 1 (0.40%) | 1 | 1 (0.40%) | 2 | 1 (1.00%) | 0.5655[2] |
| Not Related | 0 | 0 (0.00%) | 1 | 1 (0.40%) | 0 | 0 (0.00%) | 1.0000[2] |
| Solicited AEs related to Vaccine | 164 | 49 (19.60%) | 127 | 45 (18.00%) | NA | NA | 0.6471[1] |
| Severity: |  |  |  |  |  |  |  |
| Mild | 148 | 47 (18.80%) | 109 | 43 (17.20%) | NA | NA | 0.6415[1] |
| Moderate | 16 | 9 (3.60%) | 17 | 8 (3.20%) | NA | NA | 0.8051[1] |
| Severe | 0 | 0 (0.00%) | 1 | 1 (0.40%) | NA | NA | 1.0000[2] |
| Potentially life threatening | 0 | 0 (0.00%) | 0 | 0 (0.00%) | NA | NA | NA |
| Unsolicited AE (During 4 weeks (28 days) after vaccination) | 26 | 26 (10.40%) | 40 | 28 (11.20%) | 17 | 13 (13.00%) | 0.7838[1] |
| Severity: |  |  |  |  |  |  |  |
| Mild | 24 | 24 (9.60%) | 37 | 27 (10.80%) | 16 | 13 (13.00%) | 0.6458[1] |
| Moderate | 2 | 2 (0.80%) | 3 | 3 (1.20%) | 0 | 0 (0.00%) | 0.8480[2] |
| Severe | 0 | 0 (0.00%) | 0 | 0 (0.00%) | 1 | 1 (1.00%) | 0.1667[2] |
| Potentially life threatening | 0 | 0 (0.00%) | 0 | 0 (0.00%) | 0 | 0 (0.00%) | NA |
| Relatedness: |  |  |  |  |  |  |  |
| Definitely Related | 0 | 0 (0.00%) | 0 | 0 (0.00%) | 1 | 1 (1.00%) | 0.1667[2] |
| Probably Related | 0 | 0 (0.00%) | 1 | 1 (0.40%) | 0 | 0 (0.00%) | 1.0000[2] |
| Possibly related | 1 | 1 (0.40%) | 0 | 0 (0.00%) | 0 | 0 (0.00%) | 1.0000[2] |
| Unlikely related | 0 | 0 (0.00%) | 8 | 6 (2.40%) | 4 | 3 (3.00%) | 0.0152[2] |
| Not Related | 25 | 25 (10.00%) | 31 | 22 (8.80%) | 12 | 10 (10.00%) | 0.8850[1] |
| Unsolicited AEs related to vaccine | 1 | 1 (0.40%) | 9 | 7 (2.80%) | NA | NA | 0.0681[2] |
| Severity: |  |  |  |  |  |  |  |
| Mild | 1 | 1 (0.40%) | 6 | 5 (2.00%) | NA | NA | 0.2159[2] |
| Moderate | 0 | 0 (0.00%) | 3 | 3 (1.20%) | NA | NA | 0.2485[2] |
| Severe | 0 | 0 (0.00%) | 0 | 0 (0.00%) | NA | NA | NA |
| Potentially life threatening | 0 | 0 (0.00%) | 0 | 0 (0.00%) | NA | NA | NA |
| SAE | 0 | 0 (0.00%) | 0 | 0 (0.00%) | 0 | 0 (0.00%) | NA |

[Note] N: number of total participants; n: number of participants who reported events; m: number of events; %: percentages (100*n/N). NA: Not Applicable.

The p-value was derived from [1] Chi-square test or [2] Fisher’s exact test compared among Vi-DT (MD), Vi-DT (SD), and Control.

**Table S 7** Summary of Adverse Events – Age Stratum 3: 6 months to less than 2 years

| **Entire study period** | **Vi-DT (MD)**  **(N=155)** | | **Vi-DT (SD)**  **(N=155)** | | **Control**  **(N=62)** | | **P-value** |
| --- | --- | --- | --- | --- | --- | --- | --- |
|  | **m** | **n (%)** | **m** | **n (%)** | **m** | **n (%)** |  |
| Immediate Reaction (for 30 minutes after vaccination) | 5 | 4 (2.58%) | 0 | 0 (0.00%) | 1 | 1 (1.61%) | 0.1481[2] |
| Severity: |  |  |  |  |  |  |  |
| Mild | 4 | 3 (1.94%) | 0 | 0 (0.00%) | 0 | 0 (0.00%) | 0.2164[2] |
| Moderate | 0 | 0 (0.00%) | 0 | 0 (0.00%) | 1 | 1 (1.61%) | 0.1667[2] |
| Severe | 1 | 1 (0.65%) | 0 | 0 (0.00%) | 0 | 0 (0.00%) | 1.0000[2] |
| Potentially life threatening | 0 | 0 (0.00%) | 0 | 0 (0.00%) | 0 | 0 (0.00%) | NA |
| Relatedness: |  |  |  |  |  |  |  |
| Definitely Related | 5 | 4 (2.58%) | 0 | 0 (0.00%) | 1 | 1 (1.61%) | 0.1481[2] |
| Probably Related | 0 | 0 (0.00%) | 0 | 0 (0.00%) | 0 | 0 (0.00%) | NA |
| Possibly related | 0 | 0 (0.00%) | 0 | 0 (0.00%) | 0 | 0 (0.00%) | NA |
| Unlikely related | 0 | 0 (0.00%) | 0 | 0 (0.00%) | 0 | 0 (0.00%) | NA |
| Not Related | 0 | 0 (0.00%) | 0 | 0 (0.00%) | 0 | 0 (0.00%) | NA |
| Solicited AE (During 7 days after vaccination) | 35 | 21 (13.55%) | 38 | 21 (13.55%) | 25 | 10 (16.13%) | 0.8667[1] |
| Severity: |  |  |  |  |  |  |  |
| Mild | 23 | 16 (10.32%) | 32 | 18 (11.61%) | 18 | 9 (14.52%) | 0.6830[1] |
| Moderate | 11 | 6 (3.87%) | 5 | 4 (2.58%) | 6 | 3 (4.84%) | 0.6767[1] |
| Severe | 1 | 1 (0.65%) | 1 | 1 (0.65%) | 1 | 1 (1.61%) | 0.5653[2] |
| Potentially life threatening | 0 | 0 (0.00%) | 0 | 0 (0.00%) | 0 | 0 (0.00%) | NA |
| Relatedness: |  |  |  |  |  |  |  |
| Definitely Related | 19 | 14 (9.03%) | 27 | 15 (9.68%) | 16 | 9 (14.52%) | 0.4640[1] |
| Probably Related | 9 | 7 (4.52%) | 8 | 5 (3.23%) | 7 | 2 (3.23%) | 0.8124[1] |
| Possibly related | 7 | 3 (1.94%) | 3 | 2 (1.29%) | 2 | 1 (1.61%) | 1.0000[2] |
| Unlikely related | 0 | 0 (0.00%) | 0 | 0 (0.00%) | 0 | 0 (0.00%) | NA |
| Not Related | 0 | 0 (0.00%) | 0 | 0 (0.00%) | 0 | 0 (0.00%) | NA |
| Solicited AEs related to vaccine | 35 | 21 (13.55%) | 38 | 21 (13.55%) | NA | NA | 1.0000[1] |
| Severity: |  |  |  |  |  |  |  |
| Mild | 23 | 16 (10.32%) | 32 | 18 (11.61%) | NA | NA | 0.7162[1] |
| Moderate | 11 | 6 (3.87%) | 5 | 4 (2.58%) | NA | NA | 0.5203[1] |
| Severe | 1 | 1 (0.65%) | 1 | 1 (0.65%) | NA | NA | 1.0000[2] |
| Potentially life threatening | 0 | 0 (0.00%) | 0 | 0 (0.00%) | NA | NA | NA |
| Unsolicited AE (During 4 weeks (28 days) after vaccination) | 32 | 28 (18.06%) | 36 | 33 (21.29%) | 16 | 14 (22.58%) | 0.6799[1] |
| Severity: |  |  |  |  |  |  |  |
| Mild | 31 | 27 (17.42%) | 34 | 31 (20.00%) | 16 | 14 (22.58%) | 0.6614[1] |
| Moderate | 1 | 1 (0.65%) | 1 | 1 (0.65%) | 0 | 0 (0.00%) | 1.0000[2] |
| Severe | 0 | 0 (0.00%) | 1 | 1 (0.65%) | 0 | 0 (0.00%) | 1.0000[2] |
| Potentially life threatening | 0 | 0 (0.00%) | 0 | 0 (0.00%) | 0 | 0 (0.00%) | NA |
| Relatedness: |  |  |  |  |  |  |  |
| Definitely Related | 0 | 0 (0.00%) | 0 | 0 (0.00%) | 0 | 0 (0.00%) | NA |
| Probably Related | 0 | 0 (0.00%) | 0 | 0 (0.00%) | 0 | 0 (0.00%) | NA |
| Possibly related | 0 | 0 (0.00%) | 0 | 0 (0.00%) | 0 | 0 (0.00%) | NA |
| Unlikely related | 2 | 2 (1.29%) | 0 | 0 (0.00%) | 1 | 1 (1.61%) | 0.3903[2] |
| Not Related | 30 | 26 (16.77%) | 36 | 33 (21.29%) | 15 | 13 (20.97%) | 0.5665[1] |
| Unsolicited AEs related to vaccine | 2 | 2 (1.29%) | 0 | 0 (0.00%) | NA | NA | 0.4984[2] |
| Severity: |  |  |  |  |  |  |  |
| Mild | 1 | 1 (0.65%) | 0 | 0 (0.00%) | NA | NA | 1.0000[2] |
| Moderate | 1 | 1 (0.65%) | 0 | 0 (0.00%) | NA | NA | 1.0000[2] |
| Severe | 0 | 0 (0.00%) | 0 | 0 (0.00%) | NA | NA | NA |
| Potentially life threatening | 0 | 0 (0.00%) | 0 | 0 (0.00%) | NA | NA | NA |
| SAE | 2 | 2 (1.29%) | 1 | 1 (0.65%) | 0 | 0 (0.00%) | 1.0000[2] |

[Note] N: number of total participants; n: number of participants who reported events; m: number of events; %: percentages (100*n/N). NA: Not Applicable.

The p-value was derived from [1] Chi-square test or [2] Fisher’s exact test compared among Vi-DT (MD), Vi-DT (SD), and Control.

**STATISTICAL ANALYSIS PLAN**

**A phase III, multicenter, observer blind, randomized, controlled study to evaluate immune equivalence of multi-dose formulation against single-dose formulation of Vi-DT Typhoid conjugate vaccine and safety in healthy Filipino participants aged 6 months to 45 years.**

**TABLE OF CONTENTS**

[1. INTRODUCTION 17](#_Toc59493119)

[2. OBJECTIVES 17](#_Toc59493120)

[2.1 Primary objectives 17](#_Toc59493121)

[2.2 Secondary objectives 17](#_Toc59493122)

[3. STUDY OVERVIEW 17](#_Toc59493123)

[3.1 Study Design 17](#_Toc59493124)

[3.2 Sample Size 17](#_Toc59493125)

[4. STUDY ENDPOINTS 18](#_Toc59493126)

[4.1 Primary Endpoints 18](#_Toc59493127)

[4.2 Secondary Endpoints 19](#_Toc59493128)

[5. HYPOTHESES AND/OR ESTIMATION 19](#_Toc59493129)

[6. DEFINITIONS 20](#_Toc59493130)

[7. ANALYSIS SUBSETS 22](#_Toc59493131)

[7.1 Full Analysis Set 22](#_Toc59493132)

[7.2 Immunogenicity set 22](#_Toc59493133)

[7.3 Per-protocol (PP) set 22](#_Toc59493134)

[7.4 Safety Analysis Set 22](#_Toc59493135)

[8. DATA SCREENING AND ACCEPTANCE 22](#_Toc59493136)

[8.1 Data Handling and Electronic Transfer of Data 22](#_Toc59493137)

[8.2 Handling of Missing and Incomplete Data 23](#_Toc59493138)

[8.3 Distributional Characteristics 23](#_Toc59493139)

[9. STATISTICAL METHODS OF ANALYSIS 23](#_Toc59493140)

[9.1 General Principles 23](#_Toc59493141)

[9.2 Primary Analysis 24](#_Toc59493142)

[9.3 Final Analysis 24](#_Toc59493143)

[9.4 Subject Accountability 24](#_Toc59493144)

[9.5 Important Protocol Deviations 24](#_Toc59493145)

[9.6 Demographic Characteristics 25](#_Toc59493146)

[9.7 Medical History 25](#_Toc59493147)

[9.8 Prior/Concomitant Medications 25](#_Toc59493148)

[9.9 Safety Analyses 25](#_Toc59493149)

[9.9.1 Immediate Reactions 26](#_Toc59493150)

[9.9.2 Solicited Adverse Events 26](#_Toc59493151)

[9.9.3 Unsolicited Adverse Events 26](#_Toc59493152)

[9.9.4 Serious Adverse Events 26](#_Toc59493153)

[9.9.5 Vital Signs 27](#_Toc59493154)

[9.9.6 Physical Examination 27](#_Toc59493155)

[9.10 Analysis of Immunogenicity Endpoints 27](#_Toc59493156)

[9.10.1 Primary Immunogenicity Endpoint 27](#_Toc59493157)

[9.10.2 Secondary Immunogenicity Endpoint 27](#_Toc59493158)

[10. CHANGES FROM PROTOCOL-SPECIFIED ANALYSES 28](#_Toc59493159)

[11. LIST OF PLANNED TABLES, FIGURES AND LISTINGS [TFLs] 28](#_Toc59493160)

[12. APPENDICES 31](#_Toc59493161)

**LIST of abbreviation**

| AE | Adverse Event |
| --- | --- |
| ANOVA | Analysis of Variance |
| ATC | Anatomical Therapeutic Chemical |
| CI | Confidence Interval |
| CV | Coefficient of Variation |
| eCRF | Electronic Case Report Form |
| EDC | Electronic Data Capture |
| ELISA | Enzyme Linked Immunosorbent Assay |
| FAS | Full Analysis Set |
| GMT | Geometric Mean Titer |
| IgG | Immunoglobulin G |
| IP | Investigational Product |
| IPD | Important Protocol Deviation |
| IVI | International Vaccine Institute |
| MD | Multi Dose |
| M-ITT | Modified Intention-To-Treat |
| µg | Microgram |
| N | Number |
| PD | Protocol Deviation |
| PE | Phenoxy ethanol |
| PP | Per Protocol |
| PT | Preferred Term |
| SAE | Serious Adverse Event |
| SAF | Safety Set |
| SAP | Statistical Analysis Plan |
| SD | Single Dose |
| SOC | System Organ Class |
| SOP | Standard Operating Procedure |
| Vi-DT | Diphtheria Toxoid Conjugated Vi-Polysaccharide Vaccine |
| Vi-TT | Tetanus Toxoid Conjugated Vi-Polysaccharide Vaccine |
| WHO TRS | World Health Organization Technical Report Series |

**INTRODUCTION**

The purpose of this statistical analysis plan (SAP) is to provide details of the statistical analyses that have been outlined within the protocol for “*A phase III, multicenter, observer blind, randomized, controlled study to evaluate immune equivalence of multi-dose formulation against single-dose formulation of Vi-DT Typhoid conjugate vaccine and safety in healthy Filipino participants aged 6 months to 45 years*” Study (Protocol Number IVI T004) Version 5.0 dated 12JUN2020. The scope of this plan includes the primary analysis and the final analysis that are planned and will be executed by the Biostatistics and Data Management department.

**OBJECTIVES**

**Primary objectives**

- Demonstrate the immune equivalence as measured by anti-Vi IgG GMT of multi dose formulation against single dose formulation of Vi-DT (18-45-year age stratum), 4 weeks after single dose

**Secondary objectives**

- Demonstrate the immune equivalence as measured by seroconversion rates of anti-Vi IgG ELISA antibody titers of multi dose formulation against single dose formulation of Vi-DT vaccine (18-45 years age stratum) 4 weeks after single dose
- Describe safety profile in all age strata combined (age 6 months - 45 years old) and in each age stratum, 4 weeks after single dose of MD/SD formulation/control (Meningococcal conjugate Vaccine)

**STUDY OVERVIEW**

**Study Design**

This is a multicenter, randomized, observer-blinded, controlled, immune equivalence study of a multi-dose (MD) formulation with 2PE preservative of SK bioscience Vi-DT compared to single dose (SD) formulation without preservative of SK bioscience Vi-DT in 6 months - 45 years old participants including safety population.

**Sample Size**

A total of 1800 participants (1500 in Vi-DT test arm and 300 in control arm) aged 6 months to 45 years will be enrolled in this study. Participants will be randomized equally into first 2 groups of 750 participants each and third group with 300 participants within each age strata of 6 months to less than 2 years, 2 to less than 18 years and 18 to 45 years.

The sample size of the two Vi-DT groups is decided based for the immunogenicity equivalence and safety data requirements. The immunogenicity subset in adult participants, N=250 per group, will provide 94% power to show equivalence of geometric mean titres (GMT) of anti-Vi IgG at 4 weeks (28 days) after vaccination of Vi-DT (MD) and Vi-DT (SD), with the equivalence margin of [0.67, 1.5] (WHO TRS 924). Coefficient of variation (CV) of immunogenicity titre is conservatively assumed as 2.0 based on IVI T001 and T002 studies at type 1 error rate of 0.05, and 10% drop out rate is also assumed. This sample size of N=250 will provide 95% power, for equivalence tests of seroconversion rate between two formulations of Vi-DT with equivalence margin of [-10%, 10%]. In this calculation, 90% of seroconversion rate in Vi-DT is assumed (based on IVI T001) with type 1 error rate of 0.05. The sample size of N=300 control (Meningococcal vaccine) is calculated to observe at least one events with 1% of the upper limit of 95% CI for any adverse event of incidence according to rule of three.

| Group of Vaccinees and age strata | **Age Strata** | **N*** | **D0** |
| --- | --- | --- | --- |
| **Group A**  750 participants  (6 mo - 45 yrs) | 18-45 yrs | 250 | (25 µg 0.5 mL)  (Vi-DT)  Multi dose formulation |
|  | 2- <18 yrs | 250 |  |
|  | 6 mo-<2 yrs | 250 |  |
| **Group B**  750 participants  (6 mo - 45 yrs) | 18-45 yrs | 250 | (25 µg 0.5 mL)  (Vi-DT)  Single dose formulation |
|  | 2- <18 yrs | 250 |  |
|  | 6 mo-<2 yrs | 250 |  |
| **Group C**  300 participants  (6 mo - 45 yrs) | 18-45 yrs | 100 | Control  (Meningococcal Conjugate vaccine) |
|  | 2- <18 yrs | 100 |  |
|  | 6 mo-<2 yrs | 100 |  |

* Age Strata wise number of enrolled subjects may be adjusted during the actual enrolment.

**STUDY ENDPOINTS**

**Primary Endpoints**

- Geometric Mean Titers (GMT) of anti-Vi IgG at 4 weeks (28 days) after vaccination of Vi-DT(MD) vs Vi-DT (SD) [18-45 years age stratum]

**Secondary Endpoints**

- Seroconversion rates (defined as a 4-fold increase of serum anti-Vi IgG antibody titer from baseline) of anti-Vi IgG ELISA antibody titers at 4 weeks (28 days) after vaccination with Vi-DT (MD) / Vi-DT (SD) compared to baseline (D0) [18-45 years age stratum]
- Comparison of safety of Vi-DT compared to control: Vi-DT (MD) and Vi-DT (SD) will be compared separately to control vaccine and will be compared by age strata at 4 weeks after vaccination.
  - Local and systemic solicited adverse events during the 7 days after vaccination
    - Solicited local reactions at the site of injection: pain, tenderness, erythema/redness, swelling/ induration, pruritus.
    - Solicited systemic reactions (adapted to each age group): fever, lethargy, irritability, nausea/vomiting, arthralgia, diarrhoea, drowsiness, loss of appetite, chills, headache*, fatigue*, myalgia^*^, and persistent crying^†^.

^*^ Headache, fatigue and myalgia are applicable for 2 to 45 years

^†^ Persistent crying is applicable for 6 months to 2 years

- - Unsolicited adverse events during 4 weeks (28 days) after vaccination.
  - Serious Adverse Events during the entire study period.

**HYPOTHESES AND/OR ESTIMATION**

The statistical analysis will focus on comparison of immunogenicity of Vi-DT (MD; Group A) and Vi-DT (SD; Group B) at 4 weeks post vaccination. The statistical hypothesis for the primary objective is to demonstrate equivalence of two Vi-DT formulations (MD vs. SD) using GMT.

- Anti-Vi IgG GMT at 4 weeks (28 days) post Vi-DT (MD) is equivalent to GMT of Vi-DT (SD) in adults using equivalence margin of GMT ratio of [0.67, 1.5].

If the two-tailed 95% confidence interval of the ratio of GMT estimate of Vi-DT (MD) over GMT of Vi-DT (SD) is located within [0.67, 1.5], Vi-DT (MD) is equivalent to Vi-DT (SD) in terms of GMT of immunogenicity with two-sided significance level of 0.05.

The statistical hypothesis for the secondary objective is to demonstrate equivalence of two Vi-DT formulations (MD vs. SD) using seroconversion.

- Seroconversion rates of anti-Vi IgG ELISA antibody titers at 4 weeks (28 days) from baseline (D0) of Vi-DT (MD) is equivalent to seroconversion rate at 4 weeks of Vi-DT (SD) in adults using equivalence margin of 10%

If the two tailed 95% confidence interval of the estimate of difference of seroconversion rate between Vi-DT (MD) and Vi-DT (SD) at 4 weeks (Day 28) is located within [-10%, 10%], Vi-DT (MD) is equivalent to Vi-DT (SD) in terms of seroconversion rate which is defined as 4-fold increase of immunogenicity from baseline with significance level of 0.05.

**DEFINITIONS**

- **Seroconversion**: Seroconversion is defined as a 4-fold increase of serum anti-Vi IgG antibody titer from baseline measured by anti-Vi IgG ELISA
- **Seroconversion rate**: Proportion of participants who had seroconversion
- **Randomization and/or study enrollment day**: Study enrollment day is Day 0 for participants who satisfied eligibility criteria and were enrolled. A randomization number was assigned and the 1st dose vaccination was given to the enrolled participant. Participant ID is ‘Site code’-‘Age strata’, ‘Randomization number’.
- **Derivation of study days**: calculated as (Date of visit – Date of enrollment).
- **Age**: Age is calculated to Date of Enrollment – Date of Birth and described year, month, days.
- **Day 0**: Day 0 is the time of the 1st dose vaccination received after confirmed eligibility, enrollment and assigned Participant ID (Visit 2).
- **Study baseline**: For immunogenicity, baseline values are from the collected blood at Enrollment/Vaccination Visit (Visit 2, that is, Day 0) before the administration of IP.

For vital signs and anthropometrics, baseline values such as weight, height, heart rate, respiratory rate and body temperature (axillary) are collected at Screening Visit (Visit 1, Day -7 to 0) and at Enrollment/Vaccination Visit (Visit 2, Day 0) before the administration of IP. The baseline values will be the same if screening and enrollment will be performed at Day 0. The values collected at Enrollment/Vaccination Visit before the administration of IP will be the baseline values if screening and enrolment will not be performed at the same day.

For physical examinations, baseline values are examined at Screening Visit (Visit 1, Day -7 to 0) and at Enrollment/Vaccination Visit (Visit 2, Day 0) before the administration of IP. The baseline values will be the same if screening and enrollment will be performed at Day 0. The values at examined at Enrollment/Vaccination Visit before the administration of IP will be baseline values if screening and enrollment are not be performed at the same day.

- **End of study**: A participant is considered to have completed the study if he or she has completed all procedures of the study including the last visit or the last scheduled procedure.
- **Total duration of the study**: The expected total duration of the study spans from screening of first participant until last scheduled visit of the last enrolled participant.
- **Adverse Event (AE):** Any untoward medical occurrence which follows immunization, and which does not necessarily have a causal relationship with the administration of the vaccine.
- **Adverse Drug Reactions (ADR):** All noxious and unintended responses to a medicinal product related to any dose should be considered adverse reactions (AR). The phrase “responses to a medicinal product” means that a causal relationship between a medicinal product and an AE is exist with at least a reasonable possibility.
- **Immediate reactogenicity**: Immediate reactions observed in the first 30 minutes after vaccination**Solicited AEs**: Solicited AEs include immediate reactions, solicited systemic and local events. Non-consecutive events occurring during 7 days after vaccination will be counted as separate events. For severity of consecutive AE, the highest severity will be considered as severity. (i.e., mild, severe consecutive AE will be considered as one severe AE). If immediate reaction is ongoing and solicited AE of same event is observed in safety follow-up visit, it will be considered as the same event.

**- Local reactions (at the site of injection)**: Pain, tenderness, erythema/redness, swelling/induration, pruritus at the injection site during 7 days after vaccination.

**- Systemic reactions (adapted by age strata)**: Fever, lethargy, irritability, nausea/vomiting, arthralgia, diarrhea, drowsiness, loss of appetite, chills, headache, fatigue, myalgia, and persistent crying during 7 days after vaccination. Among the various systemic reactions, headache, fatigue, myalgia will be captured only for 2 to 45 years subjects while persistent crying will be captured only for 6 months to 2 years subjects. The rest of the systemic reactions will be captured in all age strata. This is also clearly mentioned in the diary cards.

- **Unsolicited AEs**: All other adverse events (those do not fall under the categories of solicited Adverse Reactions) that are identified by site staff, the site investigator and the Safety Medical Monitors during study period. They are recorded during the 4 weeks after vaccination.
- **Serious Adverse Event (SAE)**: SAE is defined as an AE meeting one of the following conditions
- Results in Death
- Life-threatening event
- Requires in-patient hospitalization >24 hours or prolongation of existing hospitalization
- Results in persistent or significant incapacity or substantial disruption of the ability to conduct normal life functions
- Congenital anomaly/birth defect
- Important medical events that may not result in death, be life-threatening, or require hospitalization may be considered serious when, based upon appropriate medical judgment, they may jeopardize the participant and may require medical or surgical intervention to prevent one of the outcomes listed in this definition

**ANALYSIS SUBSETS**

**Full Analysis Set**

The Full Analysis Set (FAS) is a modified intention-to-treat (m-ITT) analysis set that will include all participants randomized in the study who received at least one dose of investigational vaccines. This data set will be used for demographic information.

**Immunogenicity set**

The immunogenicity analysis set is a subset of FAS of those who are randomized, received at least one dose of investigational vaccines and have at least one post-baseline immunogenicity data available. The immunogenicity analysis set will be used for the primary analysis of the immunogenicity endpoints. Due to city lock down those who could not provide blood sample on Week 4 visit (4 weeks post vaccination) are not included in the immunogenicity set.

**Per-protocol (PP) set**

The per-protocol (PP) analysis set (PPS) will be a subset of the immunogenicity analysis set who do not have protocol violations (defined as major deviation from the protocol compromising the scientific integrity of the study) with regards to the inclusion/exclusion criteria, are compliant with study procedures, completed all visits as scheduled and received the correct vaccinations. A sensitivity analysis using the PP analysis sets will be conducted for the primary and secondary immunogenicity endpoints.

**Safety Analysis Set**

Safety analysis set (SAF) will include those who are randomized and received at least one dose of investigational vaccines.

**DATA SCREENING AND ACCEPTANCE**

**Data Handling and Electronic Transfer of Data**

All study data will be entered by site staffs and stored on cloud server using Medidata Rave electronic data capture (EDC) system. Immunogenicity data tested by the IVI laboratory will be transferred to Study Statistician at IVI after clinical data base lock. Study site staffs will transcribe all data collected in source documents for recording into the eCRF. The EDC system incorporates identification of the data entry errors, range and consistency checks during data entry. After all clinical data have been collected and cleaned through various quality control routine procedures, the database will be finalized. For IVI T004 study, there will be 2 database locks (DBLs) during the study period; the data until Visit 4 (Week 4; 4 weeks post vaccination), until Visit 5 (Week 24; 24 weeks post vaccination). **First** database lock will include all clinical data until Visit 4 and the immunogenicity data for baseline and 4 weeks post vaccination. **Second or Final** database lock will include all clinical data and all immunogenicity data up to 24 weeks post vaccination. Unblinding of study vaccines will be carried out after final database lock is completed.

**Handling of Missing and Incomplete Data**

The missing immunogenicity data will not be imputed. The analysis of missing pattern will be assessed, and a multiple imputation technique will be utilized as a sensitivity analysis if the missing rate of immunogenicity data is higher than 10%, which is the assumed dropout rate in the sample size.

**Distributional Characteristics**

Anti-Vi IgG ELISA antibody titers will be logarithmically transformed prior to statistical analyses to better approximate normality.

**STATISTICAL METHODS OF ANALYSIS**

**General Principles**

This study is a randomized, observer-blinded phase III study in healthy participants with age 6 months to 45 years old at the time of vaccination of investigational vaccine to assess the safety and the equivalence of immunogenicity of multi-dose formulation compared to single-dose formulation of Vi-DT in an adult participant (18-45 year age stratum).

Equivalence test of immunogenicity of two formulations will be performed as the analysis of the primary and secondary objectives. Each primary and secondary comparison for equivalence will be tested with significant level of 0.05. No multiplicity adjustment for other comparisons.

Analysis of covariance will be used to adjust for stratification and imbalances in baseline characteristics if necessary.

All reported p-values greater than or equal to 0.0001 will be rounded to four decimal point and p-values less than 0.0001 will be displayed as ‘<0.0001’. The mean and standard deviation, and percentages and 95% CI will be rounded to two decimal points.

The following additional statistical tests by variable type will be performed and the p-value derived by the statistical test will be provided.

| Variables Type | Items | Comparison |
| --- | --- | --- |
| Categorical Variable | Gender, Medical History, Prior/Concomitant Medication,  Adverse Event | Chi-square test,  Fisher’s Exact Test |
|  | Seroconversion rate comparison | Chi-square test,  Fisher’s Exact Test |
| Continuous Variable | Age,  Vital Signs | ANOVA test, Kruskal Wallis test |
|  | GMT comparison | Two sample t-test, ANOVA test among 3 groups and Dunnett’s post hoc procedure |

**Primary Analysis**

A primary analysis will be performed after all participants complete week 4 visit post-test/control vaccine dose in order to initiate the test vaccine licensure process. The immunogenicity information after vaccination and safety information within 4 weeks after vaccination will be cleaned, locked, summarized and reported. The primary analysis will be performed by an independent study statistician who will not be participated in the rest of clinical trial activities. The study and study personnel will remain blinded to individual participants’ allocation of test/comparator vaccine until the end of the study. For the primary analysis, a significant level of 0.05 using two-sided will be used for the equivalence testing of GMT and seroconversion rate of two formulations with no multiplicity adjustment. The results of primary analysis will be distributed in the aggregated way to restricted team members who would be independent of day-to-day activities or decision making for the study. The list of individuals who would have access to the primary analysis result will be documented in data integrity document. Laboratory staff performing remaining lab analysis should be prohibited from sharing the primary analysis results.

**Final Analysis**

The final analysis will be performed when all participants complete their scheduled last study visit (week 24 visit). Immunogenicity and safety data up to week 24 will be included in the final analysis.

**Subject Accountability**

Summaries of participant disposition will be based on all participants who provide informed consent/assent in the study. A flow diagram of participant disposition (CONSORT flow diagram) will illustrate the progress of participants throughout study duration from initial screening for eligibility to the completion of the study. Number and percentage (%) by vaccine groups and reasons for study discontinuation will be given for participants in the Immunogenicity and PP analysis sets.

**Important Protocol Deviations**

Major Protocol Deviations (PDs) categories are defined by the study team before the first participant visit and updated during the PD reviews throughout the study prior to database lock. With PDs categorized as Major PD, study team defines Important Protocol Deviation (IPD) for defining PP analysis set These definitions of IPD categories, sub-category codes and descriptions will be used during the study. The final IPD list is used to produce the Summary of IPDs table and the List of Subjects with IPDs.

- Violation of inclusion and exclusion criteria
- Vaccination with wrong vaccine as defined in the protocol
- Visit outside window for the immunogenicity assessment after discussion with the study medical monitor
- Missed samples for immunogenicity

**Demographic Characteristics**

Demographic characteristics and other baseline data of enrolled participants will be tabulated by vaccine group and for overall and by age strata based on the FAS. Continuous variables such as age will be summarized by number of participants, mean, standard deviation, median, minimum, and maximum. Categorical variables such as sex will be summarized by frequency and percentage in each vaccine group for overall and by age strata. If a difference in demographic characteristics among groups is suspected, the statistical significance will be compared using one-way ANOVA or Kruskal-Wallis test for continuous variables, and Chi-square test or Fisher’s exact test for categorical variables.

**Medical History**

Medical history will be summarized by SOC and PT based on the FAS. Summaries will show number and percentage of participants by vaccine group and age strata. A listing of medical history will also be provided. Medical coding of the medical histories using MedDRA version 22.1 will be added in the final analysis.

**Prior/Concomitant Medications**

The number and percentage of participants taking concomitant medication will be summarized in the FAS. The summaries will show ATC level 1 and ATC level 2. Number and percentage of participants taking prior medication will be summarized in a similar fashion to concomitant medication. A listing of prior/concomitant medication will also be provided. Coding of the concomitant medications using ATC will be added in the final analysis.

**Safety Analyses**

Safety will be descriptively summarized, including immediate reactions, solicited AEs, unsolicited AEs, SAEs, vital signs, and physical examination for the SAF. The number and proportion of participant who experienced event will be presented and the 95% confidence interval of the proportion will be calculated for vaccine groups within age strata as well as overall ages. The confidence interval of proportion in case of rate event will be calculated using Wilson’s score method. The Clopper-Pearson interval as exact method will be calculated in case of relatively small event rate.

These assessments and descriptions will not be used as decision-making criteria but will contribute to a more detailed knowledge of the safety information by the Vi-DT vaccine.

The table for summary of adverse events by severity and relatedness will be presented with number of events and number of participants with percentage. Summaries of AE will be also provided by vaccine group for overall and by age strata.

**Immediate Reactions**

The MedDRA version 22.1 will be used to code immediate reactions to a system organ class and a preferred term. Only COVID-19 terms will be coded using MedDRA version 23.1. Number and proportion of participants with immediate reactogenicity (immediate reactions within 30 minutes after vaccination) and the 95% confidence interval of the proportion will be presented for each vaccine group, and the number of immediate reactions will be summarized for severity and vaccine relatedness by vaccine group for overall and by age strata.

**Solicited Adverse Events**

The MedDRA version 22.1 will be used to code solicited adverse events to a system organ class and a preferred term. Only COVID-19 terms will be coded using MedDRA version 23.1. Number and proportion of participants with solicited adverse events 7 days post-vaccination (Day 0 to 7 or Week 0 to Week 1) and the 95% confidence interval of the proportion will be presented for each vaccine group, and the number of solicited adverse events will be summarized for severity and vaccine relatedness of the adverse events by vaccine group for overall and by age strata.

**Unsolicited Adverse Events**

The MedDRA version 22.1 will be used to code unsolicited adverse events to a system organ class and a preferred term. Only COVID-19 terms will be coded using MedDRA version 23.1. Number and proportion of participants with unsolicited adverse events from the investigational product dosing through to 4 weeks after vaccination, and the 95% confidence interval of the proportion will be presented for each vaccine group, and the number of unsolicited adverse events will be summarized for severity and vaccine relatedness of the adverse events by vaccine group for overall and by age strata.

**Serious Adverse Events**

The MedDRA version 22.1 will be used to code serious adverse events to a system organ class and a preferred term. Only COVID-19 terms will be coded using MedDRA version 23.1. The number of events, number and proportion of participants who had experienced SAEs up to week 24 will be presented by vaccine group for overall and by age strata. A listing of SAEs will be provided.

**Vital Signs**

A box plot and summary statistics such as mean, standard deviation, median, minimum, and maximum for vital signs/anthropometrics consisting of weight, height/length, heart rate, respiratory rate and body temperature changes over time will be provided. It will be provided for overall ages and by age strata.

**Physical Examination**

The shift tables of changes from baseline will be provided. Changes will be defined as the worst case during the trial, i.e., if general appearance changes to ‘not clinically significant’ and ‘normal’ at visit 3 and 4, respectively, the change is recorded as ‘clinically significant’. The shift table will include the number and percentage of changes by vaccine group for overall and by age strata.

**Analysis of Immunogenicity Endpoints**

**Primary Immunogenicity Endpoint**

The primary immunogenicity endpoint will be measured as geometric mean titer (GMT) of anti-Vi IgG of Vi-DT (MD; Group A) and Vi-DT (SD; Group B) after 4 weeks of vaccination for equivalence comparison. The GMT and associated 95% confidence interval will be provided for each vaccine group. To assess the equivalence, the test between two different formulations on GMT of Vi-DT at 4 weeks will be performed with significance level of 0.05. The equivalence of anti-Vi GMT at 4 weeks post vaccination of Vi-DT between two different formulations will be analyzed using an analysis of covariance model with vaccine group and covariate after log transformation. In case of no covariates identified, the testing will be performed using two sample t-test. The equivalence of two formulations will be confirmed if the both limits of two-tailed 95% confidence interval of the ratio of GMT between two formulations of Vi-DT is within the equivalence margin of [0.67, 1.5].

**Secondary Immunogenicity Endpoint**

The secondary immunogenicity endpoint will be measured as seroconversion rate of Vi-DT (MD) and Vi-DT (SD) after 4 weeks of vaccination for equivalence comparison.

For assessment of seroconversion rate, the proportion of participants with at least 4-fold rise anti-Vi IgG ELISA antibody titer at 4 weeks as compared to prior to the investigational product dosing (Day 0) will be calculated. The seroconversion rate and associated 95% confidence interval will be provided for each vaccine group. The equivalence of anti-Vi seroconversion at 4 weeks post vaccination of Vi-DT between two different formulations will be analyzed using the generalized linear model for binomial distribution with vaccine group and strata. The equivalence of two formulations will be confirmed if both limits of two-tailed 95% confidence interval of the difference of seroconvergence between two formulations of Vi-DT is within the equivalence margin of [-10%, 10%].

**CHANGES FROM PROTOCOL-SPECIFIED ANALYSES**

According to the protocol Version 5.0 dated 12JUN2020, the Visit window for Visit 4 for immunogenicity assessment is -3/+3 days. However, after discussion including Immunology Laboratory Scientist and Study Medical Monitor, the team agreed the Visit 4 with -3/+14 days visit window is not a major protocol deviation.

**LIST OF PLANNED TABLES, FIGURES AND LISTINGS [TFLs]**

The planned tables, figures, and listings will be provided. Templates for all planned tables are in a separate document titled IVI T004 SAP v1.0 TFLs. Followings are the contents of TFL with proper section numbers according to clinical study report template.

**10. Study Population**

*10.1 Disposition of Study Participants (Consort Flow chart)*

Figure 10.1.1. Flow diagram of Participant Disposition (CONSORT flow diagram)

*10.2 Protocol Deviation*

Table 10.2.1. Summary of Important Protocol Deviations

Listing 10.2.1. List of Important Protocol Deviations

**11. Immunogenicity Evaluation**

*11.1 Data Sets Analyzed*

Table 11.1.1. Analysis Sets

*11.2 Demographic Data of Participants and Other Baseline Information*

Table 11.2.1. Demographic information

Table 11.2.2. Summary proportion of participants with Medical history

Table 11.2.3. Distribution of Medical history

Table 11.2.4. Summary proportion of participants with Prior/concomitant medication

Table 11.2.5. Distribution of Prior/concomitant medication

*11.4 Immunogenicity Results*

*11.4.1 Analysis of immunogenicity*

Table 11.4.1.1GMT of Anti-Vi IgG – Immunogenicity set

Figure 11.4.1.1 GMT of Anti-Vi IgG – Immunogenicity set

Table 11.4.1.2. GMT of Anti-Vi IgG – PP set

Figure 11.4.1.2. GMT of Anti-Vi IgG – PP set

Table 11.4.1.3. Seroconversion rates of Anti-Vi IgG – Immunogenicity set

Figure 11.4.1.3. Seroconversion rates of Anti-Vi IgG – Immunogenicity set

Table 11.4.1.4. Seroconversion rates of Anti-Vi IgG – PP set

Figure 11.4.1.4. Seroconversion rates of Anti-Vi IgG – PP set

**12. Safety Evaluation**

*12.2 Adverse Events*

Table 12.2.1. Summary of Adverse Events

Table 12.2.2. Summary of proportion of subject with immediate reactions

Table 12.2.3. Distribution of immediate reactions

Table 12.2.4. Summary of proportion of subject with solicited AEs

Table 12.2.5. Distribution of solicited AEs

Table 12.2.6. Summary of proportion of subject with unsolicited AEs within 4 weeks after vaccination

Table 12.2.7. Distribution of unsolicited AEs 4 weeks after vaccination

Table 12.2.8. Distribution of Serious AEs

*12.3 Deaths, Other Serious Adverse Events, and Other Significant Adverse Events*

Listing 12.3.1. Deaths

*12.5 Vital Signs, Physical Findings and Other Observations Related to Safety*

Table 12.5.1. Weight

Figure 12.5.1. Weight

Table 12.5.2. Height

Figure 12.5.2. Height

Table 12.5.3. Heart Rate

Figure 12.5.3. Heart Rate

Table 12.5.4. Respiratory rate

Figure 12.5.4. Respiratory rate

Table 12.5.5. Body Temperature

Figure 12.5.5. Body Temperature

Table 12.5.6. Systolic Blood Pressure

Figure 12.5.6. Systolic Blood Pressure

Table 12.5.7. Diastolic Blood Pressure

Figure 12.5.7. Diastolic Blood Pressure

Table 12.5.8. Physical Examinations (Shift table from baseline)

**14. TFL referred to but not included in the text of CSR**

**16. APPENDICIES**

*16.1. Study Information*

Listing 16.1.7 Randomisation scheme and codes

*16.2. Participants Data Listings*

*16.2.1. Discontinued participants*

Listing 16.2.1. Discontinued Participants

*16.2.2. Protocol deviations*

Listing 16.2.2. Protocol Deviations

*16.2.3. Subjects excluded from the analysis sets*

Listing 16.2.3 Participants excluded analysis set for immunogenicity set or PP set analysis from FAS

*16.2.4. Demographic data*

Listing 16.2.4 Demographic data

*16.2.5* Compliance and/or drug concentration data (if available)

Listing 16.2.5 Compliance and/or drug concentration data (Not Applicable)

*16.2.6 Individual immunogenicity response data*

Listing 16.2.4. Individual Immunogenicity Response data

*16.2.7. Adverse event listings*

Listing 16.2.7.1. Line listings of Immediate Reactions

Listing 16.2.7.2.. Line listings of Solicited Adverse Events

Listing 16.2.7.3. Line listings of Unsolicited Adverse Events within 4 weeks post vaccination

Listing 16.2.7.4. Line listings of Serious Adverse Events

*16.4. Individual Participants Data Listings*

Listing 16.4.1. Line listings of Deaths

Listing 16.4.2. Line listings of Physical Examinations for the participant who experienced abnormality during the study period

Listing 16.4.3 Line listings of Medical History

Listing 16.4.4. Line listings of Prior/Concomitant medications

Listing 16.4.5. Participant listing of vaccine assignment

**APPENDICES**

**Appendix E.** Reference Values/Toxicity Grades

**Reference Values**

**[Solicited adverse reaction severity grading]**

Please refer to the below table (protocol 9.2.1)

| **Systemic** (**General)** | **Mild (Grade1)** | **Moderate (Grade2)** | **Severe (Grade3)** | **Potentially Life Threatening**  **(Grade 4)** |
| --- | --- | --- | --- | --- |
| Fever* | 38.0 – 38.5°C | 38.6 – 39.2°C | 39.3 – 39.9°C | > 40°C |
| Lethargy | Changes causing no or minimal interference with usual social and functional activities | Mild lethargy or somnolence causing greater than minimal interference with usual social and functional activities | Confusion, memory impairment, lethargy or somnolence causing inability to perform usual social and functional activities | Delirium OR obtunded OR Coma |
| Lethargy | No interference with routine activity | Some interference with routine activity | Significant; prevents daily routine activity | ER visit or hospitalization |
| Irritability | Require minimal or no treatment | Results in low level of inconvenience or concern | Interrupt daily activity and require drug therapy | ER visit or hospitalization |
| Nausea/ Vomiting | No interference with routine activity | Some interference with routine activity | Significant; prevents routine daily activity | ER visit or hospitalization |
| Arthralgia | No interference with routine activity | Some interference with routine activity | Significant; prevents daily routine activity | ER visit or hospitalization |
| Diarrhea | No Interference with routine activity 1-2 episodes/24 hours | Some Interference with routine activity > 2 episodes/24 hours | Prevents daily activity requires outpatient IV hydration | ER visit or hospitalization for hypotensive shock |
| Drowsiness | No interference with routine activity | Some interference with routine activity | Significant; prevents daily routine activity | ER visit or hospitalization |
| Loss of appetite | Require minimal or no treatment | Results in low level of inconvenience or concern | Require drug therapy | ER visit or hospitalization |
| Chills | No interference with routine activity | Some interference with routine activity | Significant; prevents daily routine activity | ER visit or hospitalization |
| Headache | No interference with routine activity | Some interference with routine activity | Significant; prevents daily routine activity | ER visit or hospitalization |
| Fatigue | No interference with routine activity | Some interference with routine activity | Significant; prevents daily routine activity | ER visit or hospitalization |
| Myalgia | No interference with routine activity | Some interference with routine activity | Significant; prevents daily routine activity | ER visit or hospitalization |
| Persistent crying | Require minimal or no treatment | Results in low level of inconvenience or concern | Interrupt daily activity and require drug therapy | ER visit or hospitalization |

| **Local Reaction to Injectable Product** | **Mild**  **(Grade 1)** | **Moderate**  **(Grade 2)** | **Severe**  **(Grade 3)** | **Potentially Life Threatening**  **(Grade 4)** |
| --- | --- | --- | --- | --- |
| Pain /Tenderness | Does not interfere with routine activity | Interferes with routine activity or repeated use of non-narcotic pain reliever | Prevents routine daily activity or repeated use of narcotic pain reliever | Emergency room (ER) visit or hospitalization |
| Erythema/Redness  (Adolescents & adults, age ≥12yrs)  Erythema/Redness  (Children, age 2<12 yrs)  Erythema/Redness  (Children, age < 2 yrs) | Affected area  25 - < 50mm in diameter  < 25 mm in diameter  < 10 mm in diameter | Affected area  50-<100mm in diameter  25 – 50 mm in diameter  10 < 25 mm in diameter | Affected area  ≥ 100 mm in diameter  ≥ 50 mm in diameter  25 < 50 mm in diameter | Necrosis or exfoliative dermatitis  Necrosis or exfoliative dermatitis  ≥ 50 mm in diameter |
| Swelling/Induration  (Adolescents & adults, age ≥12yrs)  Swelling/Induration  (Children, age 2<12 yrs)  Swelling/Induration  (Children, age < 2 yrs) | Affected area  25 - < 50mm in diameter  < 25 mm in diameter  < 10 mm in diameter | Affected area  50-<100mm in diameter  25 – 50 mm in diameter  10 < 25 mm in diameter | Affected area  ≥ 100 mm in diameter  ≥ 50 mm in diameter  25 < 50 mm in diameter | Necrosis or exfoliative dermatitis  Necrosis or exfoliative dermatitis  ≥ 50 mm in diameter |
| Pruritis associated  with injection | Itching localized to  injection site that is  Relieved  spontaneously or  with < 48 hours  treatment | Itching beyond the  injection site that is  not  generalized OR  Itching localized to  injection site  requiring ≥ 48  hours treatment | Generalized  itching  causing inability  to  perform usual  social &  functional  activities | NA |
